# Angular Regioselectivity in the Reactions of 2-Thioxopyrimidin-4-ones and Hydrazonoyl Chlorides: Synthesis of Novel Stereoisomeric Octahydro[1,2,4]triazolo[4,3-*a*]quinazolin-5-ones

**DOI:** 10.3390/molecules25235673

**Published:** 2020-12-01

**Authors:** Awad I. Said, Márta Palkó, Matti Haukka, Ferenc Fülöp

**Affiliations:** 1 Institute of Pharmaceutical Chemistry, University of Szeged, Eötvös u. 6, H-6720 Szeged, Hungary; awadsaid@aun.edu.eg (A.I.S.); fulop@pharm.u-szeged.hu (F.F.); 2 Chemistry Department, Faculty of Science, Assiut University, Assiut 71516, Egypt; 3 Interdisciplinary Excellence Center, Institute of Pharmaceutical Chemistry, University of Szeged, H-6720 Szeged, Hungary; 4 Department of Chemistry, University of Jyväskulä, FIN-40014 Jyväskulä, Finland; matti.o.haukka@jyu.fi

**Keywords:** regioselective reactions, hydrazonoyl chlorides, 2-thioxopyrimidin-4-ones, [1,2,4]triazolo[4,3-*a*]quinazolin-5-ones

## Abstract

The regioselective synthesis of *cis* and *trans* stereoisomers of variously functionalized octahydro[1,2,4]triazolo[4,3-*a*]quinazolin-5-ones was performed. The 2-thioxopyrimidin-4-ones used in the synthesis reacted with hydrazonoyl chlorides in a regioselective manner to produce the angular regioisomers [1,2,4]triazolo[4,3-*a*]quinazolin-5-ones rather than the linear isomers [1,2,4]triazolo[4,3-*a*]quinazolin-5-ones. The synthesis process took place with electronic control. The angular regiochemistry of the products was confirmed by X-ray experiments and two-dimensional NMR studies.

## 1. Introduction

The [1,2,4]triazolo[4,3-*a*]pyrimidinone scaffold has been known to exhibit a wide range of pharmacological activities such as antitumor, anti-inflammatory, antimicrobial, and antifungal activity, as well as macrophage activation [1,2,3,4,5,6,7,8,9].

A reaction between hydrazonoyl chlorides decorated with different functionalities [10,11,12] and 2-thioxopyrimidin-4-ones is an efficient strategy for incorporating the [1,2,4]triazolo moiety into [1,2,4]triazolo[4,3-*a*]pyrimidinones [13,14].

Recently, we reported that 2-thioxopyrimidin-4-one constructed on the norbornene skeleton gave an angular regioisomer ([1,2,4]triazolo[4,3-*a*]pyrimidin-7(1*H*)-one), functionalized with various hydrazonoyl chlorides, as the sole product of the reaction [15]. This was in contrast to findings observed previously, where [1,2,4]triazolo[4,3-*a*]pyrimidin-5(1*H*)-one, the linear regioisomer, was the sole product of the reaction [16,17,18,19,20,21].

Herein, we report the extension of our research for the regioselective synthesis of novel *cis*- and *trans*-octahydro[1,2,4]triazolo[4,3-*a*]quinazolin-5-ones **4a**–**g** and **5a**–**g** via the reaction of cyclohexane-fused *cis*- or *trans*-2-thioxopyrimidin-4-ones **1** and **2** with hydrazonoyl chlorides **3a**–**g**, taking place under electronic control. Moreover, X-ray and two-dimensional NMR studies were used to prove the stereochemistry of the products.

## 2. Results and Discussion

**Scheme 1:**
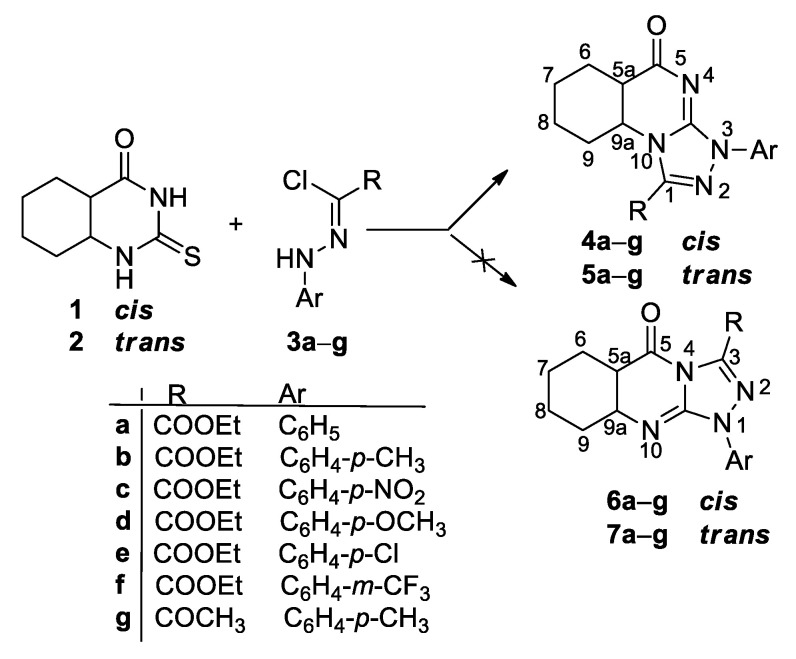
Synthesis of [1,2,4]triazolo[4,3-*a*]quinazolin-5(3*H*)-one **4a**–**g** and **5a**–**g**.

**Scheme 2:**
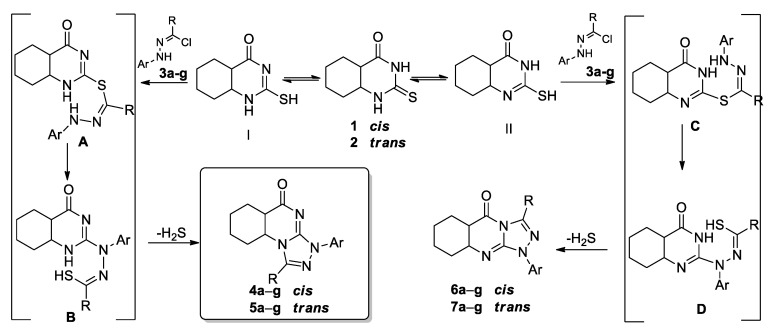
Proposed reaction pathways to form angular and linear regioisomers.

Cyclohexane-fused *cis*- and *trans*-2-thioxopyrimidin-4-one **1** and **2** were prepared according to previously described procedures [22]. The thioxopyrimidinone derivatives **1** or **2** thus prepared were reacted with the hydrazonoyl chlorides **3a**–**g** bearing varied functionalities in dioxane in the presence of triethylamine as a base under reflux conditions (Scheme 1). According to the reaction mechanism depicted in Scheme 2, the angular regioisomers [1,2,4]triazolo[4,3-*a*]quinazolin-5(3*H*)-one 4a–g and 5a–g and linear regioisomers [1,2,4]triazolo[4,3-*a*]quinazolin-5(3*H*)-one **6a**–**g** and **7a**–**g** were expected to be formed. The outcome of the reactions depends on the involvement of the tautomeric structures **I** or **II** of the cyclohexane-fused 2-thioxopyrimidin-4-ones **1** and **2**. The reactions proceeded through S-alkylation [17,18,19,20,21] to give S-alkylated products **A** followed by Smiles rearrangement [23], affording intermediates **B**, which cyclized in situ under the employed reaction conditions via the elimination of hydrogen sulfide gas to give the desired products **4a**–**g** and **5a**–**g** [20]. As evidenced by TLC and NMR spectroscopy, the transformations took place in a regioselective manner, producing the corresponding angular regioisomers as the sole products.

**Figure 1:**
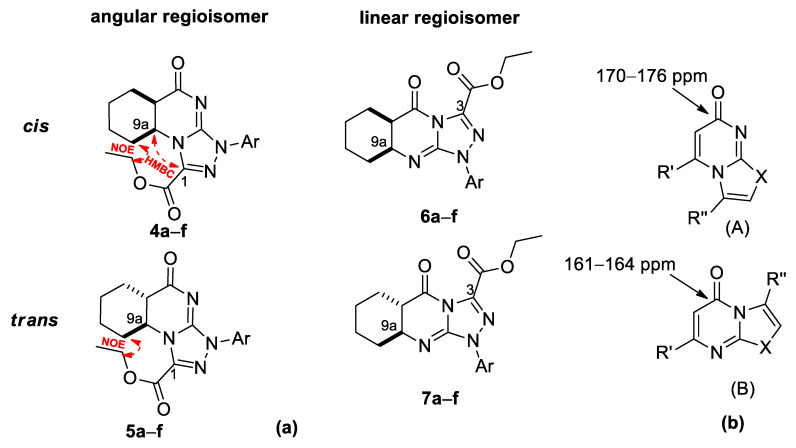
(**a**) Heteronuclear multiple bond correlation (HMBC) and neighboring Overhauser effect (NOE) mutual correlations in angular regioisomers, and the lack of a similar correlation in their linear counterparts. (**b**) ^13^C-NMR data used for assigning the stereochemistry of the products.

**Figure 2:**
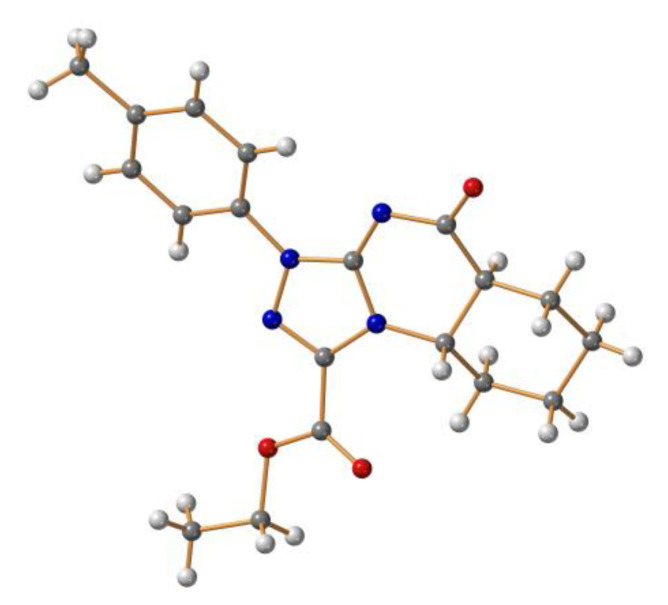
TELP image of **5b** at 50% probability level.

The steric structure of the angular regioisomers was evidenced with information acquired through various instrumental techniques, namely, ^1^H-NMR, ^13^C-NMR, and two-dimensional NMR including NOESY (neighboring Overhauser effect spectroscopy correlation), HMBC (heteronuclear multiple bond correlation), and X-ray crystallographic analysis. The ^1^H-NMR spectra of the products formed by the hydrazonoyl chloride ethyl esters **3a**–**f** show a more multiplicated signal pattern corresponding to the CH_2_ moiety of the ester functional group (Supplementary Materials), which suggests the steric proximity of the ester group and the cyclohexane skeleton. Moreover, the NOESY spectra exhibit a mutual correlation between the hydrogens of CH_2_ and cyclohexane. In addition, the HMBC spectra show a mutual correlation between H-9a and C-1, which are separated by three bonds in the angular regioisomers. However, this correlation cannot exist in the linear regioisomers, because the C-3 and H-9a atoms are separated by five bonds (Figure 1a). Last but not least, the ^13^C-NMR spectra reveal the signal of the carbonyl carbon of the pyrimidinone ring residue at nearly 176 ppm. These chemical shift values are similar to those of annelated pyrimidinones of type **A** rather than those of type **B** (Figure 1b) [24]. Finally, the X-ray crystallographic analysis of **5b** provided conclusive evidence for the angular regiochemistry of the products (Figure 2).

On the basis of the above evidence, the angular structures **4a**–**g** and **5a**–**g** were assigned for the products, and, consequently, the linear structures **6a**–**g** and **7a**–**g** could be rejected.

The regioselectivity of these reactions delivering the angular regioisomers was ascribed to electronic factors rather than steric factors. That is, since the tautomeric form **I** is electronically and energetically predominant, the reaction proceeds through tautomeric form **I** and leads to the formation of the angular regioisomer (Scheme 2).

## 3. Materials and Methods

### 3.1. General Methods

NMR analyses were performed at 500.20 MHz for ^1^H-NMR and at 125.62 MHz for ^13^C-NMR in CDCl_3_ at room temperature, using a Bruker AV NEO Ascend 500 spectrometer (Bruker Biospin, Karlsruhe, Germany) with a Double Resonance Broad Band Probe (BBO). Tetramethylsilane (TMS) was used as an internal standard. The reactions were monitored by thin-layer chromatography (TLC) using aluminum sheets coated with silica gel (POLYGRAM^®^SIL G/UV254, Merck, Kenilworth, NJ, USA). The TLC plates were visualized under UV light. The melting points were measured using a Hinotek-X4 micro melting point apparatus (Hinotek, Ningbo, China).

The cyclohexane-fused *cis*- and *trans*-2-thioxopyrimidin-4-ones **1** and **2** were prepared from the corresponding amino esters according to reported procedures [25,26,27]. The hydrazonoyl chlorides **2a**–**h** were synthesized according to procedures reported previously [27,28].

X-ray diffraction data were collected on a Rigaku Oxford Diffraction Supernova diffractometer using Cu Kα radiation, measured at a temperature of 120 K using a crystal of **5b** immersed in cryo-oil and mounted in a loop. The *CrysAlisPro* [29] software package was used for cell refinement and data reduction. An analytical absorption correction (*CrysAlisPro*) was applied to the intensities before structure solution. The structure was solved by an intrinsic phasing method (*SHELXT* [30,31]). Structural refinement was carried out using the *SHELXL* [30] software with the *SHELXLE* [31] graphical user interface. Hydrogen atoms were positioned geometrically and constrained to ride on their parent atoms, with C–H = 0.95–1.00 Å and U_iso_ = 1.2–1.5·U_eq_ (parent atom). The crystallographic details are summarized in Table S1.

### 3.2. Synthesis of Cis- and Trans-[1,2,4]triazolo[4,3-a]quinazolin-5(3H)-one ***4a**–**g*** and ***5a**–**g***

A mixture of 0.5 mmol of cyclohexane-fused 2-thioxopyrimidin-4-one **1** or **2** and 0.5 mmol of hydrazonoyl chloride (**3a**–**g**) in dioxane (10 mL) was treated at reflux temperature in the presence of 100 µL of triethylamine (TEA) for 5–7 h. The reactions were monitored by TLC (*n*-hexane/EtOAC = 1:1 as the eluent) until completion. After solvent evaporation under reduced pressure, the residue was dissolved in CHCl_3_ (20 mL), followed by extraction with water (3 × 10 mL). The CHCl_3_ solution was dried on Na_2_SO_4_, the solvent was evaporated, and the residue was purified by column chromatography using *n*-hexane/EtOAC = 1:1 as the eluent.

(5aR*,9aS*)-Ethyl 5-oxo-3-phenyl-3,5,5a,6,7,8,9,9a-octahydro-[1,2,4]triazolo[4,3-a]quinazoline-1-carboxylate (**4a**): 69%, m.p. 223–225 °C ^1^H NMR (500 MHz, CDCl_3_) δ = 8.09 (d, *J* = 7.7, 2H), 7.45 (t, *J* = 8.0, 2H), 7.33 (t, *J* = 7.4, 1H), 5.08–4.98 (m, 1H, H-4a), 4.58–4.45 (m, 2H, *CH_2_*CH_3_), 2.92 (d, *J* = 4.2, 1H), 2.68 (d, *J* = 12.5, 1H), 2.03 (d, *J* = 9.5, 1H), 1.86 (d, *J* = 10.9, 1H), 1.47 (t, *J* = 7.1, 3H, CH_2_*CH_3_*), 1.51–1.41 (m, 5H). ^13^C NMR (126 MHz, CDCl_3_) δ = 176.0(C=O), 156.3 (C=O), 153.2(C), 136.85(C), 136.3(C), 129.1(CH), 127.8(CH), 121.8(CH), 63.3(OCH_2_), 55.4(CH), 55.2(CH), 38.2(CH_2_), 28.8(CH_2_), 24.7(CH_2_), 24.6(CH_2_), 21.22(CH), 14.29, 14.1(CH_3_).

(5aR*,9aS*)-Ethyl 5-oxo-3-(p-tolyl)-3,5,5a,6,7,8,9,9a-octahydro-[1,2,4]triazolo[4,3-a]quinazoline-1-carboxylate (**4b**): 62%, m.p. 263–264 °C. ^1^H NMR (500 MHz, CDCl_3_) δ = 7.94 (d, *J* = 8.5, 2H), 7.24 (d, *J* = 8.3, 2H), 5.10–4.95 (m, 1H, H-4a), 4.52 (pd, *J* = 7.6, 3.6, 1H, *CH_2_*CH_3_), 2.91 (d, *J* = 5.5, 1H, H-8a), 2.68 (d, *J* = 12.2, 1H), 2.37 (s, 3H, *p*-tolyl), 2.03 (d, *J* = 12.1, 1H), 1.86 (d, *J* = 11.1, 1H), 1.47 (t, *J* = 7.1, 3H, CH_2_*CH_3_*). 1.62–1.4 (m, 4H). ^13^C NMR (126 MHz, CDCl_3_) δ = 176.1(C=O), 156.4(C=O), 153.1(C), 137.9(C), 136.6(C), 133.9(C), 129.7(CH), 121.7(CH), 63.3(OCH_2_), 55.3(CH), 38.1(CH), 28.8(CH_2_), 24.7(CH_2_), 24.6(CH_2_), 21.3 (CH_2_), 21.11(CH_3_, *p*-tolyl), 14.13(CH_2_*CH_3_*).

(5aR*,9aS*)-Ethyl 5-oxo-3-(4-nitrophenyl)-3,5,5a,6,7,8,9,9a-octahydro-[1,2,4]triazolo[4,3-a]quinazoline-1-carboxylate (**4c**): 61%, m.p. 262–265 °C. ^1^H NMR (500 MHz, CDCl_3_) δ = 8.51 (d, *J* = 9.3, 2H), 8.33 (d, *J* = 12.2, 2H), 5.07 (ddd, *J* = 11.3, 6.5, 4.4, 1H), 4.60–4.49 (m, 2H, *CH_2_*CH_3_), 2.95 (d, *J* = 6.0, 1H), 2.68 (d, *J* = 8.0, 1H), 2.05 (d, *J* = 12.5, 1H), 1.88 (d, *J* = 10.2, 1H), 1.50 (t, *J* = 7.1, 3H, CH_2_*CH_3_*), 1.63–1.43 (m, 5H). ^13^C NMR (126 MHz, CDCl_3_) δ = 176.0(C=O), 156.0(C=O), 155.6(C), 146.0(C), 141.4(C), 137.6(C), 124.8(CH), 121.2(CH), 77.3(O*CH_2_*), 77.0(CH), 76.8(CH), 63.7(CH_2_), 55.5(CH), 38.2(CH), 28.8(CH_2_), 24.6(CH_2_), 24.5(CH_2_), 21.1(CH_2_), 14.11(CH_3_).

(5aR*,9aS*)-Ethyl 5-oxo-3-(4-methoxyphenyl)-3,5,5a,6,7,8,9,9a-octahydro-[1,2,4]triazolo[4,3-a]quinazoline-1-carboxylate (**4d**): 69%, m.p. 215–216 °C. ^1^H NMR (500 MHz, CDCl_3_) δ 7.94 (d, *J* = 9.1 Hz, 2H), 6.96 (d, *J* = 9.1 Hz, 2H), 5.15–4.92 (m, 1H), 4.67–4.39 (m, 2H, *CH_2_*CH_3_), 3.83 (s, 3H), 2.93 (d, *J* = 5.7 Hz, 1H), 2.68 (d, *J* = 12.2 Hz, 1H), 2.03 (d, *J* = 12.5 Hz, 1H), 1.86 (d, *J* = 12.5 Hz, 2H), 1.47 (t, *J* = 7.1 Hz, 3H, CH_2_*CH_3_*). 1.62–1.4 (m, 4H). ^13^C NMR (126 MHz, CDCl_3_) δ 175.9(C=O), 159.1(C=O), 156.3(C), 152.9(C), 136.6(C), 129.2(C), 123.7(CH), 114.3(CH), 63.3(OCH_2_), 55.6(OCH_3_), 55.4(CH), 38.2(CH), 28.8(CH_2_), 24.7(CH_2_), 24.62, 2(CH_2_).25, 1(CH_2_).14.1(CH_3_).

(5aR*,9aS*)-Ethyl 5-oxo-3-(4-chlorophenyl)-3,5,5a,6,7,8,9,9a-octahydro-[1,2,4]triazolo[4,3-a]quinazoline-1-carboxylate (**4e**): 68%, m.p. 239–241 °C. ^1^H NMR (500 MHz, CDCl_3_) δ = 8.12 (d, *J* = 9.0, 2H), 7.42 (d, *J* = 9.1, 2H), 5.04 (ddd, *J* = 11.3, 6.5, 4.4, 1H), 4.62–4.45 (m, 2H,CH_2_CH_3_), 2.92 (d, *J* = 6.0, 1H), 2.68 (d, *J* = 7.5, 1H), 2.03 (d, *J* = 12.4, 1H), 1.86 (d, *J* = 10.3, 1H), 1.48 (t, *J* = 7.1, 3H, CH_2_CH_3_), 1.69–1.41 (m, 5H). ^13^C NMR (126 MHz, CDCl_3_) δ = 176.0(C=O), 156.2(C=O), 153.1(C), 136.9(C), 134.9(C), 133.4(C), 129.3(CH), 122.7(CH), 63.5(OCH_2_), 55.4(CH), 38.1(CH), 28.8(CH_2_), 24.6(CH_2_), 24.6(CH_2_), 21.2(CH_2_), 14.1(CH_3_).

(5aR*,9aS*)-Ethyl 5-oxo-3-(4-(trifluoromethyl)phenyl)-3,5,5a,6,7,8,9,9a-octahydro-[1,2,4]triazolo[4,3-a]quinazoline-1-carboxylate (**4f**): 62%, m.p. 202–206 °C. ^1^H NMR (500 MHz, CDCl_3_) δ = 8.56 (dd, *J* = 8.3, 3.0, 1H), 8.27 (s, 1H), 7.62–7.58 (m, 2H), 5.20–4.89 (m, 1H), 4.66–4.39 (m, 2H, CH_2_CH_3_), 2.94 (d, *J* = 3.8, 1H), 2.68 (d, *J* = 9.9, 1H), 2.05 (dd, *J* = 8.7, 3.7, 1H), 1.87 (d, *J* = 10.2, 1H), 1.74 (s, 1H), 1.50 (t, *J* = 7.1, 3H, CH_2_CH_3_), 1.61–1.41 (m, 4H). ^13^C NMR (126 MHz, CDCl_3_) δ = 176.02 (C=O), 156.16(C=O), 153.38(C), 137.2(C), 136.84(C), 131.7(q, *J* = 38 Hz, CCF_3_), 129.9(CH), 124.3(q, *J* = 3.5 Hz, CHCCF_3_), 123.5(q, *J* = 273 Hz, CF_3_), 118.2(q, *J* = 4 Hz, CHCCF_3_), 63.6(OCH_2_), 55.4(CH), 38.2(CH), 28.8(CH_2_), 24.6(CH_2_), 24.6(CH_2_), 21.2(CH_2_), 14.1(CH_3_).

(5aR*,9aS*)-1-Acetyl-3-(p-tolyl)-5a,6,7,8,9,9a-hexahydro[1,2,4]triazolo[4,3-a]quinazoline-5(3H)-one (**4g**): 66%**,** m.p. 196–198 °C. ^1^H NMR (500 MHz, CDCl_3_) δ = 7.96 (d, *J* = 8.5, 2H), 7.27 (d, *J* = 7.0, 2H), 5.13–5.00 (m, 1H), 2.92 (d, *J* = 2.3, 1H), 2.69 (s, 3H, CO*CH_3_*), 2.66 (d, *J* = 7.7, 1H), 2.39 (s, 3H, CH_3_, *p*-tolyl), 1.98 (d, *J* = 12.3, 1H), 183 (br, 2H), 1.62–1.45 (m, 4H). ^13^C NMR (126 MHz, CDCl_3_) δ 188.1(C=O), 176.0(C=O), 153.6(C), 141.4(C), 138.1(C), 133.9(C), 129.8(CH), 121.6(CH), 55.0(CH), 38.2(CH_2_), 28.6(CO*C*H_3_), 26.5, 24.6(CH_2_), 24.5(CH_2_), 21.3(CH_2_), 21.1(CH_3_, *p*-tolyl).

(5aR*,9aR*)-Ethyl 5-oxo-3-phenyl-3,5,5a,6,7,8,9,9a-octahydro-[1,2,4]triazolo[4,3-a]quinazoline-1-carboxylate (**5a**): 65%, m.p. 203–206 °C. ^1^H NMR (500 MHz, CDCl_3_) δ 8.04 (d, *J* = 7.6 Hz, 2H), 7.48–7.41 (m, 2H), 7.33 (t, *J* = 7.4 Hz, 1H), 4.58–4.44 (m, 2H, *CH_2_*CH_3_), 4.08–3.97 (m, 1H), 2.82 (d, *J* = 7.5 Hz, 1H), 2.50 (d, *J* = 13.0 Hz, 1H), 2.30–2.21 (m, 2H), 1.94 (t, *J* = 9.2 Hz, 1H), 1.46 (t, *J* = 7.1 Hz, 3H, CH_2_*CH_3_*), 1.54–1.35 (m. 4H). ^13^C NMR (126 MHz, CDCl_3_) δ 176.7(C=O), 157.5(C=O), 153.3(C), 138.9(C), 136.2(C), 129.1(CH), 127.7(CH), 121.7(CH), 63.8(OCH_2_), 58.2(CH), 43.4(CH), 31.2(CH_2_), 25.4(CH_2_), 25.0(CH_2_), 24.2(CH_2_), 14.0. (CH_3_).

(5aR*,9aR*)-Ethyl 5-oxo-3-(p-tolyl)-3,5,5a,6,7,8,9,9a-octahydro-[1,2,4]triazolo[4,3-a]quinazoline-1-carboxylate (**5b**): 67%, m.p. 213–214 °C ^1^H NMR (500 MHz, CDCl_3_) δ 7.89 (d, *J* = 8.5 Hz, 2H), 7.24 (d, *J* = 8.2 Hz, 2H), 4.71–4.35 (m, 2H, *CH_2_*CH_3_), 4.17–3.87 (m, 1H), 2.82 (d, *J* = 7.4 Hz, 1H), 2.50 (d, *J* = 10.5 Hz, 1H), 2.37 (s, 3H), 2.32–2.19 (m, 1H), 1.93 (t, *J* = 7.3 Hz, 2H), 1.46 (t, *J* = 7.1 Hz, 3H, CH_2_*CH_3_*). 1.47–1.132 (m, 4H). ^13^C NMR (126 MHz, CDCl_3_) δ 176.7(C=O), 157.5(C=O), 153.2(C), 138.7(C), 137.8(C), 133.8(C), 129.7(CH), 121.7(CH), 63.7(CH_2_), 58.2(CH), 43.4(CH), 31.2(CH_2_), 25.4(CH_2_), 25.1(CH_2_), 24.2(CH_2_), 21.1(CH_3_, *p*-tolyl), 14.0(CH_3_).

(5aR*,9aR*)-Ethyl 5-oxo-3-(4-nitrophenyl)-3,5,5a,6,7,8,9,9a-octahydro-[1,2,4]triazolo[4,3-a]quinazoline-1-carboxylate (**5c**): 71%, m.p. 255–258 °C. ^1^H NMR (500 MHz, CDCl_3_) δ 8.47–8.42 (m, 2H), 8.34–8.27 (m, 2H), 4.54 (qq, *J* = 10.8, 7.2 Hz, 2H, *CH_2_*CH_3_), 4.14–3.97 (m, 1H), 2.90–2.70 (m, 1H), 2.50 (dd, *J* = 17.3, 6.6 Hz, 1H), 2.33–2.20 (m, 1H), 1.96 (dd, *J* = 11.5, 6.0 Hz, 2H), 1.49 (t, *J* = 7.2 Hz, 3H, CH_2_*CH_3_*), 1.56–1.34 (m, 4H). ^13^C NMR (126 MHz, CDCl_3_) δ 176.5(C=O), 157.2(C=O), 153.5(C), 145.9(C), 141.3(C), 139.7(C), 124.8(CH), 121.0(CH), 64.1(OCH_2_), 58.2(CH), 43.3(CH), 31.1(CH_2_), 25.3(CH_2_), 24.9(CH_2_), 24.1(CH_2_), 14.0(CH_3_).

(5aR*,9aR*)-Ethyl 5-oxo-3-(4-methoxyphenyl)-3,5,5a,6,7,8,9,9a-octahydro-[1,2,4]triazolo[4,3-a]quinazoline-1-carboxylate (**5d**): 69%, m.p. 204–206 °C ^1^H NMR (500 MHz, CDCl_3_) δ 7.92–7.85 (m, 2H), 6.99–6.91 (m, 2H), 4.58–4.41 (m, 2H, *CH_2_*CH_3_), 4.06–3.98 (m, 1H), 3.83 (s, 3H), 2.83 (dt, *J* = 15.4, 7.6 Hz, 1H), 2.51 (d, *J* = 12.8 Hz, 1H), 2.28–2.20 (m, 1H), 1.93 (t, *J* = 7.9 Hz, 2H), 1.46 (t, *J* = 7.1 Hz, 3H, CH_2_*CH_3_*), 1.53–1.25 (m,4H). ^13^C NMR (126 MHz, CDCl_3_) δ 176.7(C=O), 159.0(C=O), 157.5(C), 153.1(C), 138.7(C), 129.3(C), 123.6(CH), 114.3(CH), 63.7(OCH_2_), 58.3(CH), 55.6(CH), 43.4(OCH_3_), 31.2(CH_2_), 25.4(CH_2_), 25.1(CH_2_), 24.2(CH_2_), 14.0(CH_3_).

(5aR*,9aR*)-Ethyl 5-oxo-3-(4-chlorophenyl)-3,5,5a,6,7,8,9,9a-octahydro-[1,2,4]triazolo[4,3-a]quinazoline-1-carboxylate (**5e**): 68%, m.p. 240–245 °C. ^1^H NMR (500 MHz, CDCl_3_) δ 8.07 (d, *J* = 8.9 Hz, 2H), 7.41 (d, *J* = 8.9 Hz, 2H), 4.51 (qq, *J* = 10.8, 7.1 Hz, 2H, *CH_2_*CH_3_), 4.06–3.95 (m, 1H), 2.79 (d, *J* = 7.7 Hz, 1H), 2.49 (d, *J* = 12.5 Hz, 1H), 2.25 (t, *J* = 12.2 Hz, 1H), 1.94 (t, *J* = 8.7 Hz, 2H), 1.46 (t, *J* = 7.1 Hz, 3H, CH_2_*CH_3_*), 1.53–1.26 (m, 4H). ^13^C NMR (126 MHz, CDCl_3_) δ 176.4(C=O), 157.4(C=O), 153.2(C), 139.0(C), 134.9(C), 133.2(C), 129.2(CH), 122.6(CH), 63.8(OCH_2_), 58.3(CH), 43.4(CH), 31.2(CH_2_), 25.4(CH_2_), 25.0(CH_2_), 24.2(CH_2_), 14.0(CH_3_).

(5aR*,9aR*)-Ethyl 5-oxo-3-(4-(trifluoromethyl)phenyl)-3,5,5a,6,7,8,9,9a-octahydro-[1,2,4]triazolo[4,3-a]quinazoline-1-carboxylate (**5f**): 70%, m.p. 173–175 °C. ^1^H NMR (500 MHz, CDCl_3_) δ 8.60–8.42 (m, 1H), 8.22 (d, *J* = 0.6 Hz, 1H), 7.66–7.50 (m, 2H), 4.60–4.36 (m, 2H, CH_2_CH_3_), 4.13–3.93 (m, 1H). 2.80 (d, *J* = 8.0 Hz, 1H), 2.50 (d, *J* = 13.0 Hz, 1H), 2.34–2.19 (m, 1H), 1.95 (t, *J* = 8.4 Hz, 2H), 1.48 (t, *J* = 7.2 Hz, 3H, CH_2_CH_3_), 1.55–1.23 (m, 4H). ^13^C NMR (126 MHz, CDCl_3_) δ 176.6(C=O), 157.3(C=O), 153.4(C), 139.3(C), 136.8(C), 131.7 (q, *J* = 33 Hz, C-CF_3_), 129.9, 124.8, 124.1 (q, *J* = 3.6 Hz, CHCCF_3_), 123.4 (q, *J* = 271 Hz, CF_3_), 118.1 (q, *J* = 3.7 Hz), 64.0(OCH_2_), 58.2(CH), 43.4(CH), 31.2(CH_2_), 25.3(CH_2_), 25.0(CH_2_), 24.2(CH_2_), 14.0(CH_3_).

(5aR*,9aR*)-1-Acetyl-3-(p-tolyl)-5a,6,7,8,9,9a-hexahydro[1,2,4]triazolo[4,3-a]quinazoline-5(3H)-one (**5g**): 67%, m.p. 158–162 °C ^1^H NMR (500 MHz, CDCl_3_) δ 7.91 (d, *J* = 8.5 Hz, 2H), 7.26 (d, *J* = 8.2 Hz, 2H), 4.06–3.98 (m, 1H), 2.92 (d, *J* = 8.5 Hz, 1H), 2.71 (s, 3H), 2.49 (d, *J* = 13.2 Hz, 1H), 2.39 (s, 3H), 2.25 (t, *J* = 13.5 Hz, 1H), 1.92 (d, *J* = 11.0 Hz, 2H), 1.49 (dd, *J* = 24.4, 14.6 Hz, 1H), 1.42–1.22 (m, 3H). ^13^C NMR (126 MHz, CDCl_3_) δ 187.9(C=O), 176.8(C=O), 153.7(C), 144.2(C), 138.0(C), 133.8(C), 129.7(CH), 121.6(CH), 58.5(CO*C*H_3_), 43.6(CH), 31.9(CH_2_), 27.6(CH), 25.5(CH_2_), 25.2(CH_2_), 24.3(CH_2_), 21.1(CH_3_, *p*-tolyl).

## 4. Conclusions

Herein, we report the unexpected regioselectivity of the reaction between 2-thioxopyrimidin-4-ones with hydrazonoyl chlorides to produce the angular regioisomers [1,2,4]triazolo[4,3-*a*]quinazolin-5-ones, rather than the linear isomers [1,2,4]triazolo[4,3-*a*]quinazolin-5-ones. The transformations are controlled by electronic factors of 2-thioxopyrimidin-4-one. This phenomenon was exploited in the synthesis of the novel stereoisomeric octahydro[1,2,4]triazolo[4,3-*a*]quinazolin-5-ones **4a**–**g** and **5a**–**g** starting from *cis* or *trans* cyclohexane-fused 2-thioxopyrimidin-4-one **1** or **2**, respectively. The stereochemistry of the products was assigned on the basis of one- and two-dimensional NMR spectra and by X-ray measurements providing conclusive evidence.

## Supplementary Materials

NMR spectra of all the synthesized compounds and crystallographic data for **5b** are available online.
