# Supplementary material for: Angular Regioselectivity in the Reactions of 2-Thioxopyrimidin-4-ones and Hydrazonoyl Chlorides: Synthesis of Novel Stereoisomeric Octahydro[1,2,4]triazolo[4,3-a]quinazolin-5-ones
Source: Molecules. 2020 Dec 1;25(23):5673. doi: 10.3390/molecules25235673 (PMC7730367; doi:10.3390/molecules25235673)
Supplement: Supplementary file 1 [file molecules-25-05673-s001.pdf]

Angular Regioselectivity in the reactions of 2-Thioxopyrimidin-4-ones and Hydrazonoyl Chlorides: Synthesis of Novel Steroisomeric Octahydro[1,2,4]triazolo[4,3-*a*]quinazolin-5-ones

Awad I. Said,[1,3] Márta Palkó*,[1,2] Matti Haukka[4] and Ferenc Fülöp[1,2,]

[1] Institute of Pharmaceutical Chemistry, University of Szeged, Eötvös u. 6, H-6720 Szeged, Hungary,

[2] Institute of Pharmaceutical Chemistry, University of Szeged, Interdisciplinary excellence center, H-6720 Szeged, Hungary.

E-mail: palko.marta@szte.hu (M.P.); fulop@pharm.u-szeged.hu (F.F.)

[3] Chemistry Department, Faculty of Science, Assiut University, Assiut, Egypt 71516

E-mail: awadsaid@aun.edu.eg

[4] Department of Chemistry, University of Jyväskulä, FIN-40014, Jyväskulä, Finland

E-mail: matti.o.haukka@jyu.fi

* Correspondence: E-mail: fulop@pharm.u-szeged.hu; Tel.: +36 62 545564; Fax: +36 62 545705

Table of Contents

(5aR,9aS*)-Ethyl 5-oxo-3-phenyl-3,5,5a,6,7,8,9,9a-octahydro-[1,2,4]triazolo[4,3-a]quinazoline-1-carboxylate* **(4a)** **2**

(5aR,9aS*)-Ethyl 5-oxo-3-(p-tolyl)-3,5,5a,6,7,8,9,9a-octahydro-[1,2,4]triazolo[4,3-a]quinazoline-1-carboxylate* **(4b)** **6**

(5aR,9aS*)-Ethyl 5-oxo-3-(4-nitrophenyl)-3,5,5a,6,7,8,9,9a-octahydro-[1,2,4]triazolo[4,3-a]quinazoline-1-carboxylate* **(4c)** **10**

(5aR,9aS*)-Ethyl 5-oxo-3-(4-methoxyphenyl)-3,5,5a,6,7,8,9,9a-octahydro-[1,2,4]triazolo[4,3-a]quinazoline-1-carboxylate* **(4d)** **13**

(5aR,9aS*)-Ethyl 5-oxo-3-(4-chlorophenyl)-3,5,5a,6,7,8,9,9a-octahydro-[1,2,4]triazolo[4,3-a]quinazoline-1-carboxylate* **(4e)** **16**

(5aR,9aS*)-Ethyl 5-oxo-3-(4-(trifluoromethyl)phenyl)-3,5,5a,6,7,8,9, 9a-octahydro-[1,2,4]triazolo[4,3-a]quinazoline-1-carboxylate* **(4f)** **20**

(5aR,9aS*)-1-Acetyl-3-(p-tolyl)-5a,6,7,8,9,9a-hexahydro-[1,2,4]triazolo[4,3-a]quinazoline-5(3H)-one* **(4g)** **22**

(5aR,9aR*)-Ethyl 5-oxo 3-phenyl-3,5,5a,6,7,8,9, 9a-octahydro-[1,2,4]triazolo[4,3-a]quinazoline-1-carboxylate* **(5a)** **23**

(5aR,9aR*)-Ethyl 5-oxo 3-(p-tolyl)-3,5,5a,6,7,8,9,9a-octahydro-[1,2,4]triazolo[4,3-a]quinazoline-1-carboxylate* **(5b)** **26**

(5aR,9aR*)-Ethyl 5-oxo -3-(4-nitrophenyl)-3,5,5a,6,7,8,9,9a-octahydro-[1,2,4]triazolo[4,3-a]quinazoline-1-carboxylate* **(5c)** **27**

(5aR,9aR*)-Ethyl 5-oxo-3-(4-methoxyphenyl)-3,5,5a,6,7,8,9,9a-octahydro-[1,2,4]triazolo[4,3-a]quinazoline-1-carboxylate* **(5d)** **30**

(5aR,9aR*)-Ethyl 5-oxo-3-(4-chlorophenyl)-3,5,5a,6,7,8,9,9a-octahydro-[1,2,4]triazolo[4,3-a]quinazoline-1- carboxylate* **(5e)** **33**

(5aR,9aR*)-Ethyl 5-oxo-3-(4-(trifluoromethyl)phenyl)-3,5,5a,6,7,8,9,9a-octahydro-[1,2,4]triazolo[4,3-a]quinazoline-1-carboxylate* **(5f)** **36**

(5aR,9aR*)-1-Acetyl-3-(p-tolyl)-5a,6,7,8,9,9a-hexahydro[1,2,4]triazolo[4,3-a]quinazoline-5(3H)-one* **(5g)** **39**

Crystallographic details of compound **5b** **40**

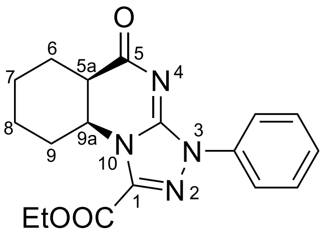

(5a*R,9a*S**)-Ethyl 5-oxo-3-phenyl-3,5,5a,6,7,8,9,9a-octahydro-[1,2,4]triazolo[4,3-*a*] quinazoline-1-carboxylate (4a)**

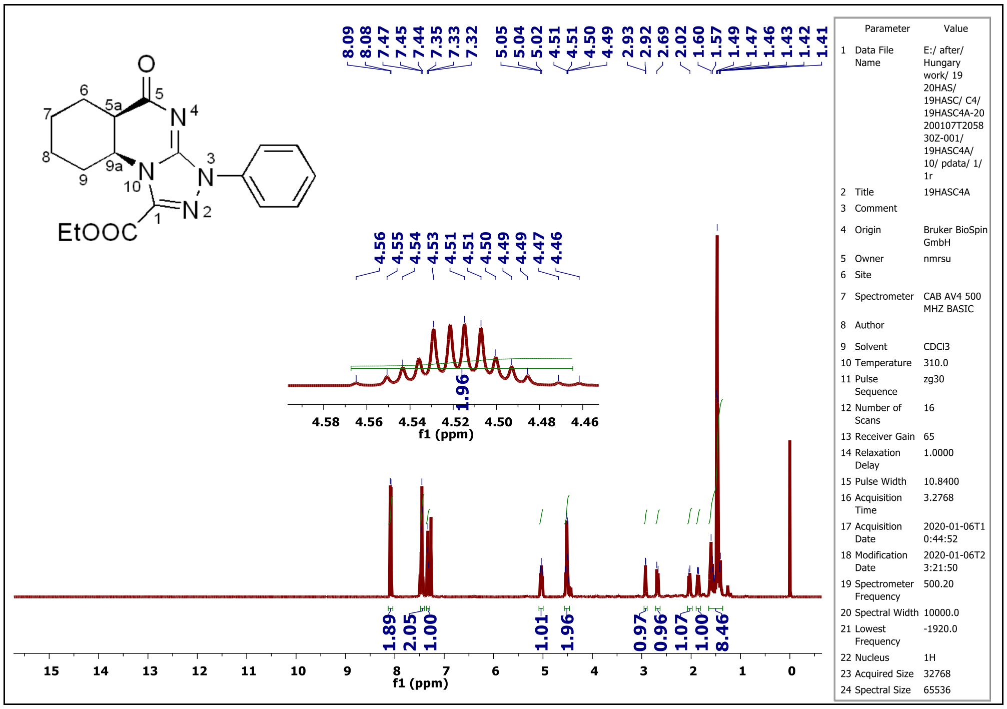

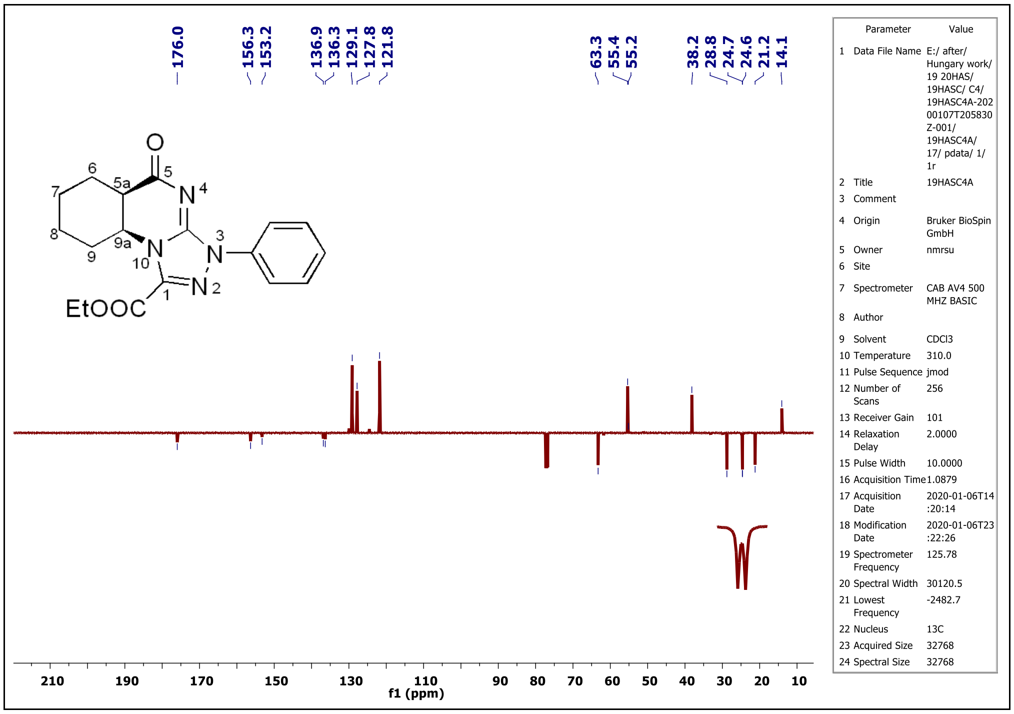

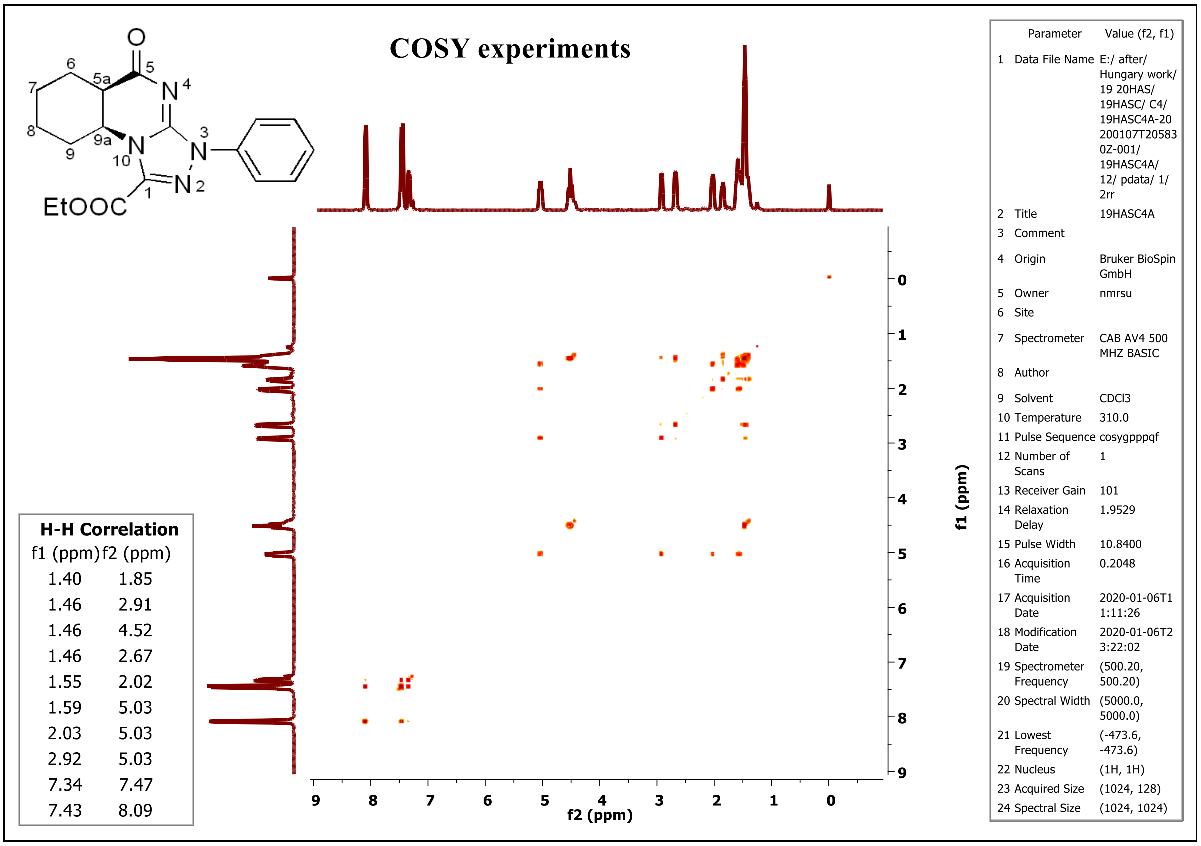

H-H Correlation	
f1 (ppm)	f2 (ppm)
1.40	1.85
1.46	2.91
1.46	4.52
1.46	2.67
1.55	2.02
1.59	5.03
2.03	5.03
2.92	5.03
7.34	7.47
7.43	8.09

	Parameter	Value (f2, f1)
1	Data File Name	E:/ after/ Hungary work/ 19 20HAS/ 19HASC/ C4/ 19HASC4A-20200107T205830Z-001/ 19HASC4A/ 12/ pdata/ 1/ 2rr
2	Title	19HASC4A
3	Comment	
4	Origin	Bruker BioSpin GmbH
5	Owner	nmrsu
6	Site	
7	Spectrometer	CAB AV4 500 MHZ BASIC
8	Author	
9	Solvent	CDCl3
10	Temperature	310.0
11	Pulse Sequence	cosygpppqf
12	Number of Scans	1
13	Receiver Gain	101
14	Relaxation Delay	1.9529
15	Pulse Width	10.8400
16	Acquisition Time	0.2048
17	Acquisition Date	2020-01-06T11:11:26
18	Modification Date	2020-01-06T23:22:02
19	Spectrometer Frequency	(500.20, 500.20)
20	Spectral Width	(5000.0, 5000.0)
21	Lowest Frequency	(-473.6, -473.6)
22	Nucleus	(1H, 1H)
23	Acquired Size	(1024, 128)
24	Spectral Size	(1024, 1024)

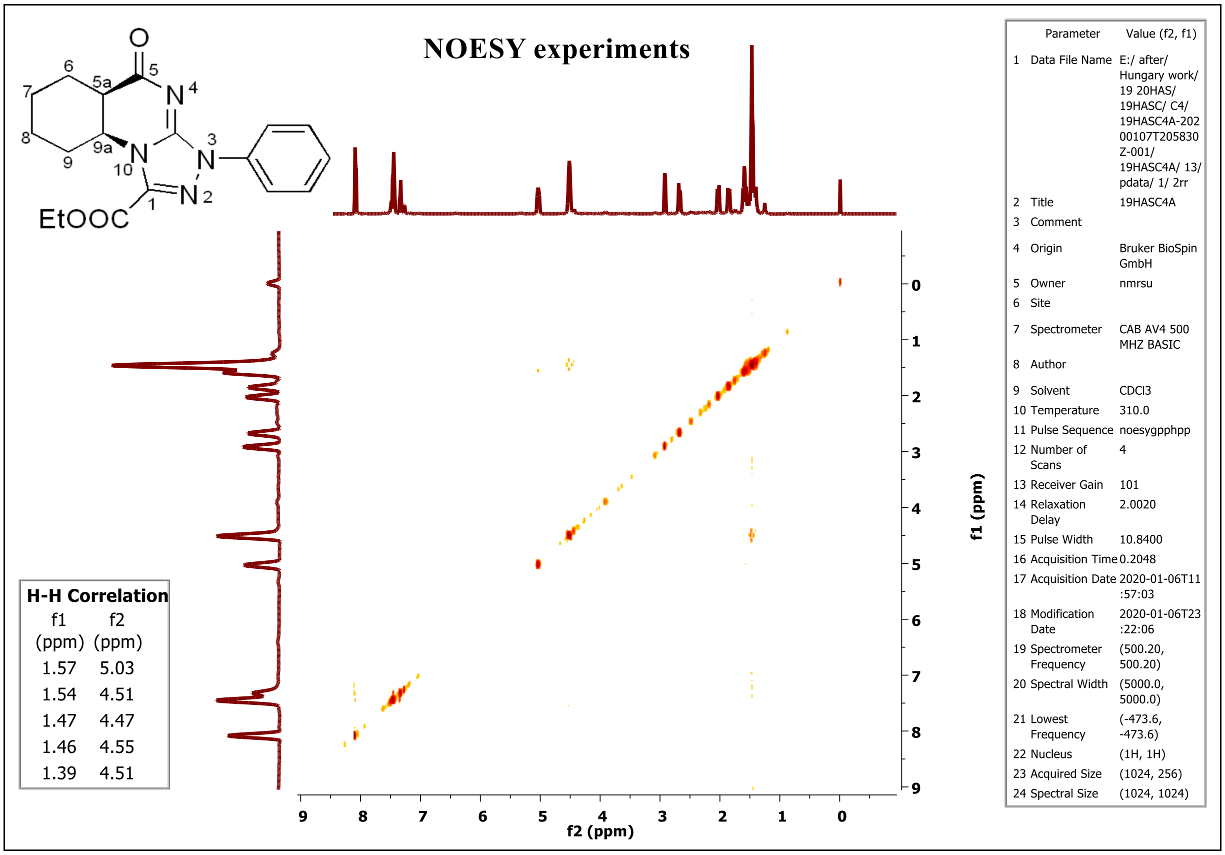

H-H Correlation	
f1 (ppm)	f2 (ppm)
1.57	5.03
1.54	4.51
1.47	4.47
1.46	4.55
1.39	4.51

	Parameter	Value (f2, f1)
1	Data File Name	E:/ after/ Hungary work/ 19 20HAS/ 19HASC/ C4/ 19HASC4A-20200107T205830Z-001/ 19HASC4A/ 13/ pdata/ 1/ 2rr
2	Title	19HASC4A
3	Comment	
4	Origin	Bruker BioSpin GmbH
5	Owner	nmrsu
6	Site	
7	Spectrometer	CAB AV4 500 MHZ BASIC
8	Author	
9	Solvent	CDCl3
10	Temperature	310.0
11	Pulse Sequence	noesygpphpp
12	Number of Scans	4
13	Receiver Gain	101
14	Relaxation Delay	2.0020
15	Pulse Width	10.8400
16	Acquisition Time	0.2048
17	Acquisition Date	2020-01-06T11:57:03
18	Modification Date	2020-01-06T23:22:06
19	Spectrometer Frequency	(500.20, 500.20)
20	Spectral Width	(5000.0, 5000.0)
21	Lowest Frequency	(-473.6, -473.6)
22	Nucleus	(1H, 1H)
23	Acquired Size	(1024, 256)
24	Spectral Size	(1024, 1024)

TOCSY experiments

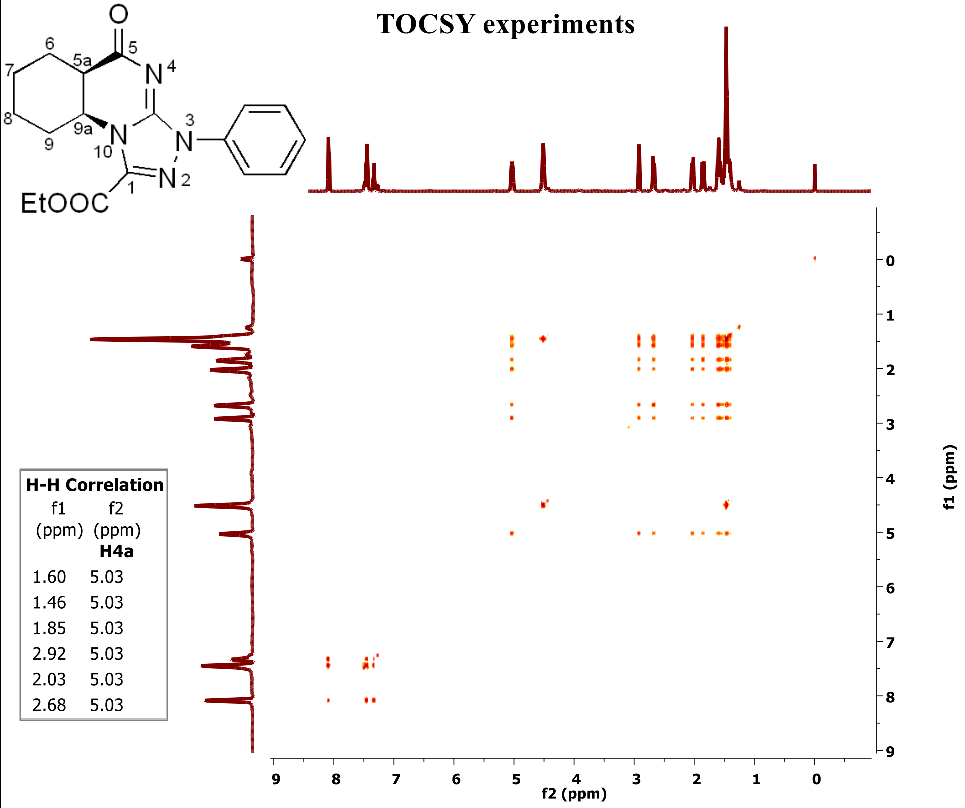

H-H Correlation

f1 (ppm)	f2 (ppm)
	**H4a**
1.60	5.03
1.46	5.03
1.85	5.03
2.92	5.03
2.03	5.03
2.68	5.03

	Parameter	Value (f2, f1)
1	Data File Name	E:/ after/ Hungary work/ 19 20HAS/ 19HASC/ C4/ 19HASC4A-202 00107T205830 Z-001/ 19HASC4A/ 14/ pdata/ 1/ 2rr
2	Title	19HASC4A
3	Comment	
4	Origin	Bruker BioSpin GmbH
5	Owner	nmrsu
6	Site	
7	Spectrometer	CAB AV4 500 MHZ BASIC
8	Author	
9	Solvent	CDCl3
10	Temperature	310.0
11	Pulse Sequence	mlevphpp
12	Number of Scans	8
13	Receiver Gain	101
14	Relaxation Delay	2.0020
15	Pulse Width	10.8400
16	Acquisition Time	0.2048
17	Acquisition Date	2020-01-06T13:17:47
18	Modification Date	2020-01-06T23:22:12
19	Spectrometer Frequency	(500.20, 500.20)
20	Spectral Width	(5000.0, 5000.0)
21	Lowest Frequency	(-473.6, -473.6)
22	Nucleus	(1H, 1H)
23	Acquired Size	(1024, 256)
24	Spectral Size	(1024, 1024)

HSQC experiments

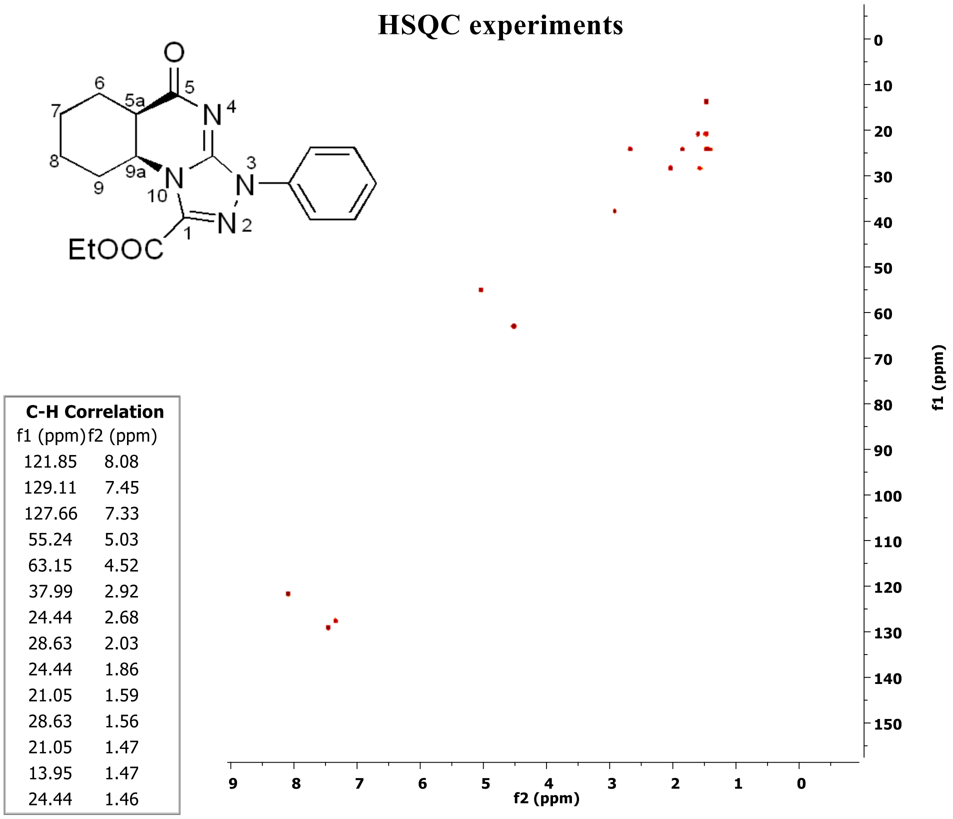

C-H Correlation

f1 (ppm)	f2 (ppm)
121.85	8.08
129.11	7.45
127.66	7.33
55.24	5.03
63.15	4.52
37.99	2.92
24.44	2.68
28.63	2.03
24.44	1.86
21.05	1.59
28.63	1.56
21.05	1.47
13.95	1.47
24.44	1.46

	Parameter	Value (f2, f1)
1	Data File Name	E:/ after/ Hungary work/ 19 20HAS/ 19HASC/ C4/ 19HASC4A-202 00107T205830 Z-001/ 19HASC4A/ 15/ pdata/ 1/ 2rr
2	Title	19HASC4A
3	Comment	
4	Origin	Bruker BioSpin GmbH
5	Owner	nmrsu
6	Site	
7	Spectrometer	CAB AV4 500 MHZ BASIC
8	Author	
9	Solvent	CDCl3
10	Temperature	310.0
11	Pulse Sequence	hsqcetgp
12	Number of Scans	2
13	Receiver Gain	101
14	Relaxation Delay	1.5000
15	Pulse Width	10.8400
16	Acquisition Time	0.1024
17	Acquisition Date	2020-01-06T13:34:09
18	Modification Date	2020-01-06T23:22:16
19	Spectrometer Frequency	(500.20, 125.78)
20	Spectral Width	(5000.0, 20752.3)
21	Lowest Frequency	(-473.6, -943.0)
22	Nucleus	(1H, 13C)
23	Acquired Size	(512, 256)
24	Spectral Size	(1024, 1024)

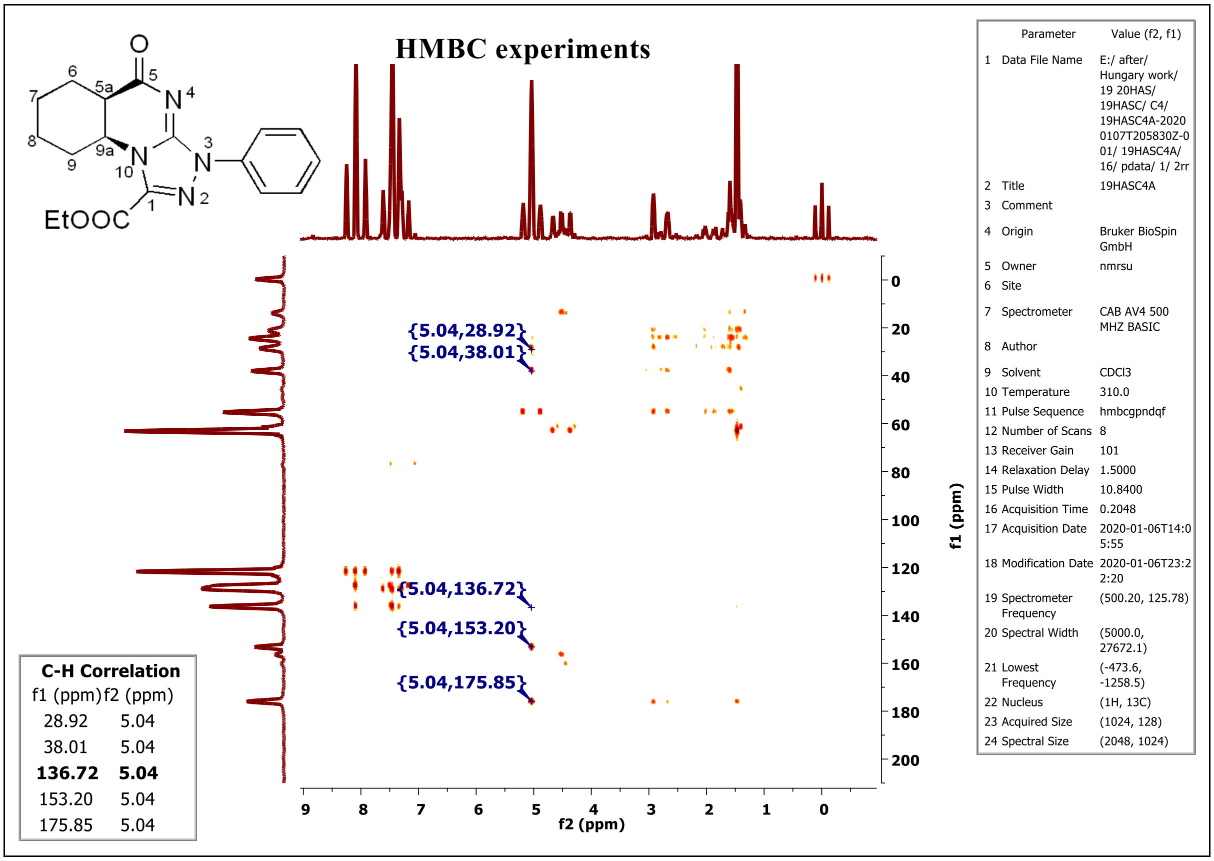
HMBC experiments
EtOOC
{5.04,28.92}
{5.04,38.01}
{5.04,136.72}
{5.04,153.20}
{5.04,175.85}
f2 (ppm)
f1 (ppm)
C-H Correlation
f1 (ppm) f2 (ppm)
28.92 5.04
38.01 5.04
136.72 5.04
153.20 5.04
175.85 5.04
Parameter Value (f2, f1)
1 Data File Name E:/ after/ Hungary work/ 19 20HAS/ 19HASC/ C4/ 19HASC4A-2020 0107T205830Z-0 01/ 19HASC4A/ 16/ pdata/ 1/ 2rr
2 Title 19HASC4A
3 Comment
4 Origin Bruker BioSpin GmbH
5 Owner nmrsu
6 Site
7 Spectrometer CAB AV4 500 MHZ BASIC
8 Author
9 Solvent CDCl3
10 Temperature 310.0
11 Pulse Sequence hmbcgpndqf
12 Number of Scans 8
13 Receiver Gain 101
14 Relaxation Delay 1.5000
15 Pulse Width 10.8400
16 Acquisition Time 0.2048
17 Acquisition Date 2020-01-06T14:05:55
18 Modification Date 2020-01-06T23:22:20
19 Spectrometer Frequency (500.20, 125.78)
20 Spectral Width (5000.0, 27672.1)
21 Lowest Frequency (-473.6, -1258.5)
22 Nucleus (1H, 13C)
23 Acquired Size (1024, 128)
24 Spectral Size (2048, 1024)

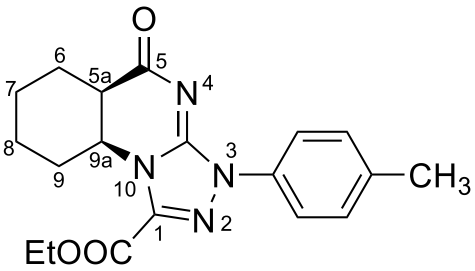

(5a*R,9a*S**)-Ethyl 5-oxo-3-(*p*-tolyl)-3,5,5a,6,7,8,9,9a-octahydro-[1,2,4]triazolo[4,3-*a*]quinazoline-1-carboxylate (4b)**

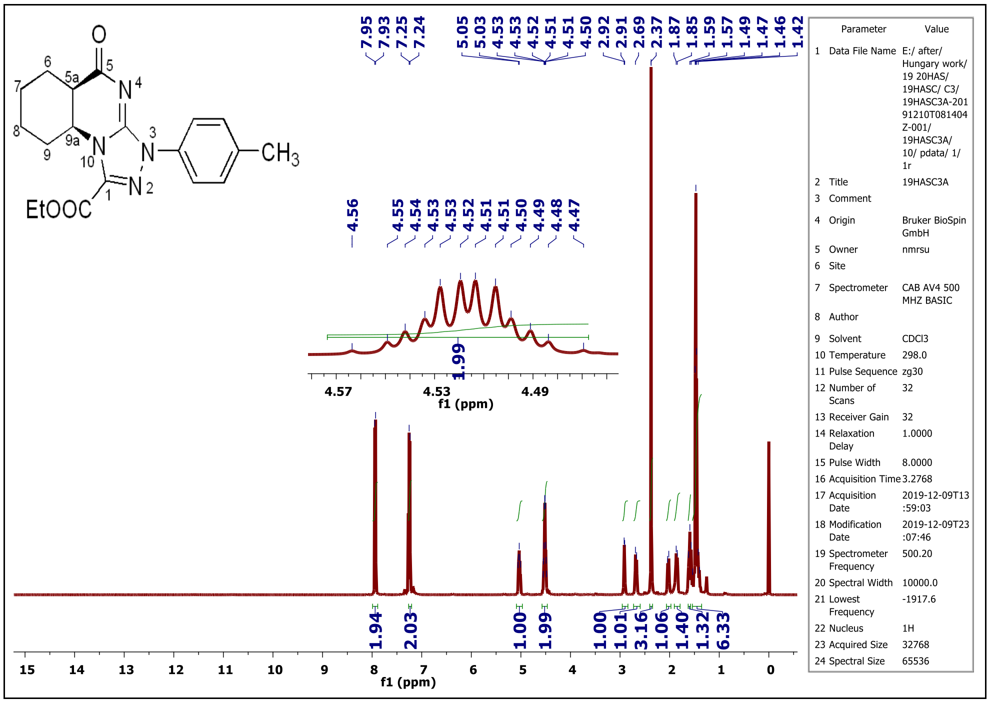

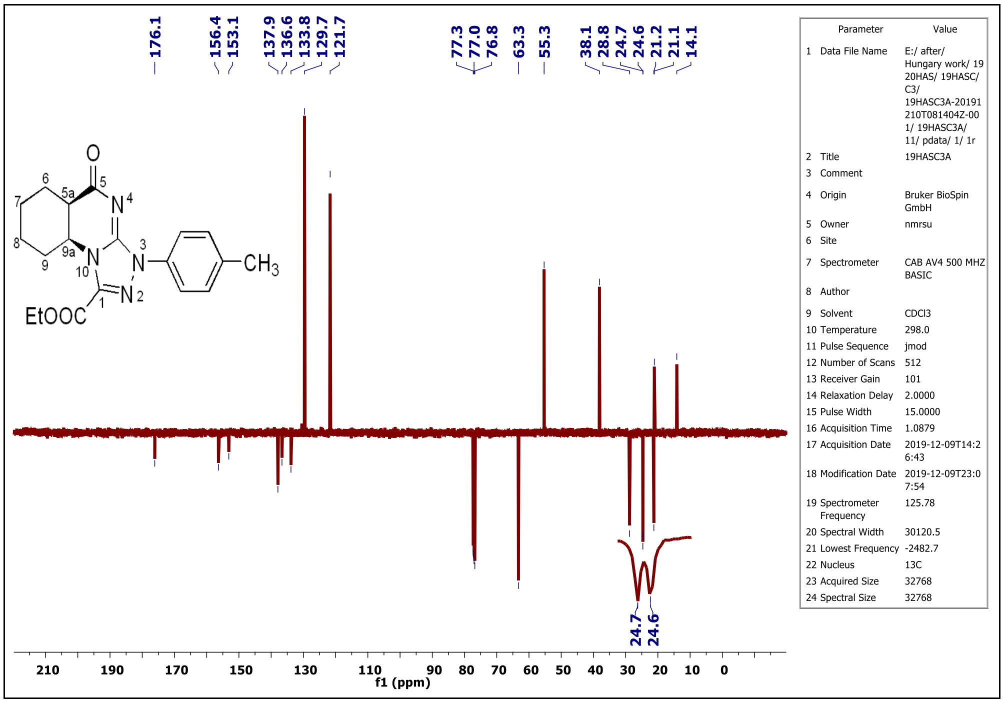

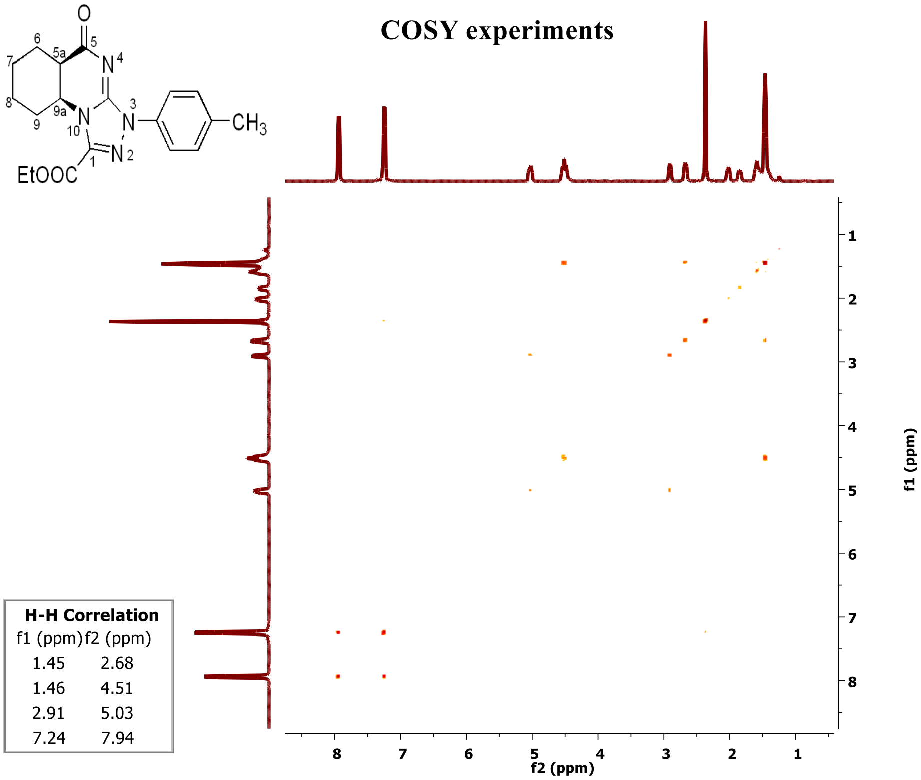

H-H Correlation

f1 (ppm)	f2 (ppm)
1.45	2.68
1.46	4.51
2.91	5.03
7.24	7.94

	Parameter	Value (f2, f1)
1	Data File Name	E:/ after/ Hungary work/ 19 20HAS/ 19HASC/ C3/ 19HASC3A/ 19HASC3A-20 191210T08140 4Z-001/ 19HASC3A/ 12/ pdata/ 1/ 2rr
2	Title	19HASC3A
3	Comment	
4	Origin	Bruker BioSpin GmbH
5	Owner	nmrsu
6	Site	
7	Spectrometer	CAB AV4 500 MHZ BASIC
8	Author	
9	Solvent	CDCl3
10	Temperature	298.1
11	Pulse Sequence	cosygpppqf
12	Number of Scans	1
13	Receiver Gain	64
14	Relaxation Delay	1.9140
15	Pulse Width	8.0000
16	Acquisition Time	0.2458
17	Acquisition Date	2019-12-09T14:32:26
18	Modification Date	2019-12-09T23:08:00
19	Spectrometer Frequency	(500.20, 500.20)
20	Spectral Width	(4166.7, 4166.7)
21	Lowest Frequency	(210.5, 210.5)
22	Nucleus	(1H, 1H)
23	Acquired Size	(1024, 128)
24	Spectral Size	(1024, 1024)

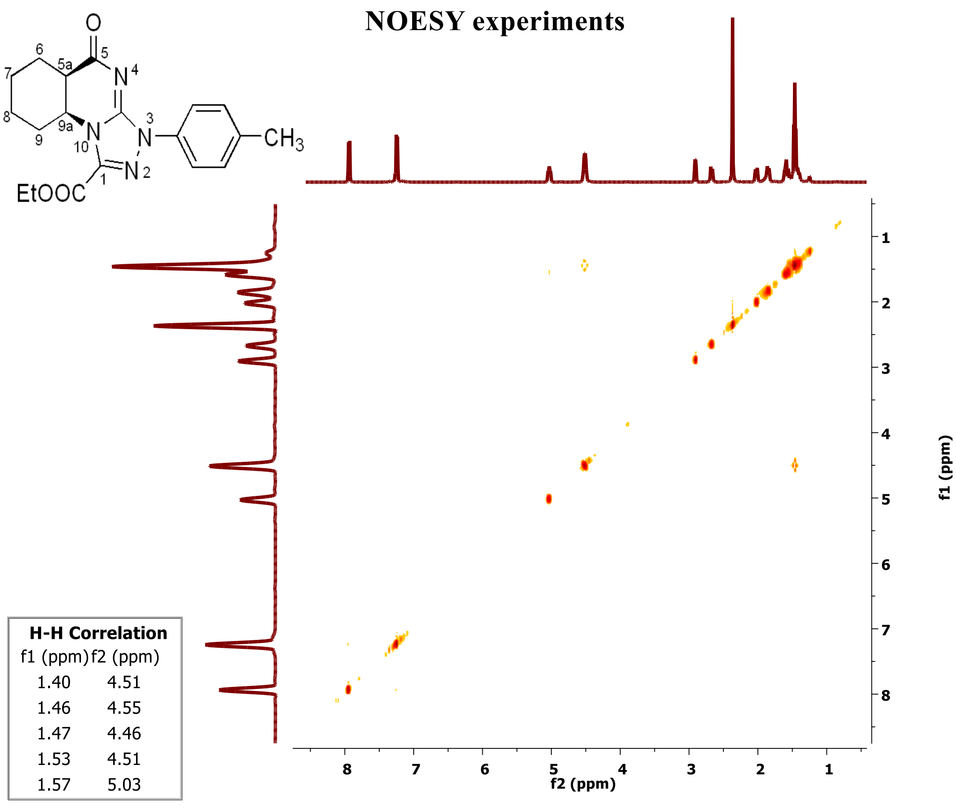

H-H Correlation

f1 (ppm)	f2 (ppm)
1.40	4.51
1.46	4.55
1.47	4.46
1.53	4.51
1.57	5.03

	Parameter	Value (f2, f1)
1	Data File Name	E:/ after/ Hungary work/ 19 20HAS/ 19HASC/ C3/ 19HASC3A-201 91210T081404 Z-001/ 19HASC3A/ 13/ pdata/ 1/ 2rr
2	Title	19HASC3A
3	Comment	
4	Origin	Bruker BioSpin GmbH
5	Owner	nmrsu
6	Site	
7	Spectrometer	CAB AV4 500 MHZ BASIC
8	Author	
9	Solvent	CDCl3
10	Temperature	298.0
11	Pulse Sequence	noesygpphpp
12	Number of Scans	4
13	Receiver Gain	64
14	Relaxation Delay	1.9631
15	Pulse Width	8.0000
16	Acquisition Time	0.2458
17	Acquisition Date	2019-12-09T15:17:59
18	Modification Date	2019-12-09T23:08:08
19	Spectrometer Frequency	(500.20, 500.20)
20	Spectral Width	(4166.7, 4166.7)
21	Lowest Frequency	(210.5, 210.5)
22	Nucleus	(1H, 1H)
23	Acquired Size	(1024, 256)
24	Spectral Size	(1024, 1024)

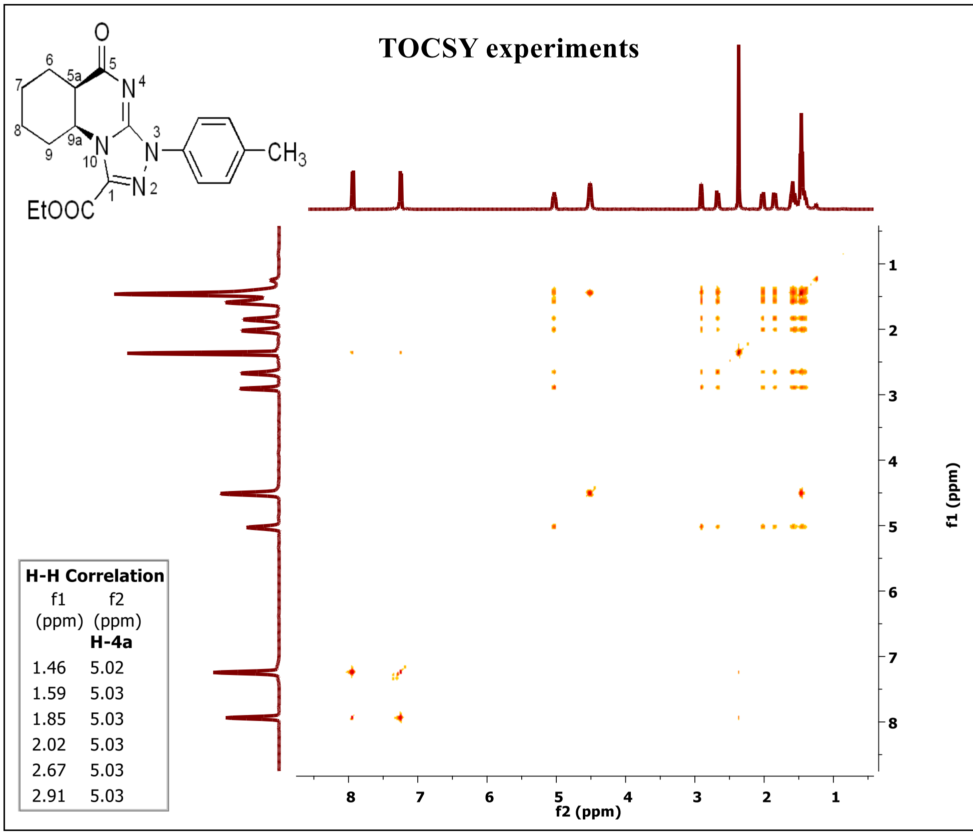

H-H Correlation	
f1 (ppm)	f2 (ppm)
	H-4a
1.46	5.02
1.59	5.03
1.85	5.03
2.02	5.03
2.67	5.03
2.91	5.03

	Parameter	Value (f2, f1)
1	Data File Name	E:/ after/ Hungary work/ 19 20HAS/ 19HASC/ C3/ 19HASC3A-20191210T081404Z-001/ 19HASC3A/ 14/ pdata/ 1/ 2rr
2	Title	19HASC3A
3	Comment	
4	Origin	Bruker BioSpin GmbH
5	Owner	nmrsu
6	Site	
7	Spectrometer	CAB AV4 500 MHZ BASIC
8	Author	
9	Solvent	CDCl3
10	Temperature	298.0
11	Pulse Sequence	mlevphpp
12	Number of Scans	8
13	Receiver Gain	64
14	Relaxation Delay	1.9631
15	Pulse Width	8.0000
16	Acquisition Time	0.2458
17	Acquisition Date	2019-12-09T16:38:50
18	Modification Date	2019-12-09T23:08:16
19	Spectrometer Frequency	(500.20, 500.20)
20	Spectral Width	(4166.7, 4166.7)
21	Lowest Frequency	(210.5, 210.5)
22	Nucleus	(1H, 1H)
23	Acquired Size	(1024, 256)
24	Spectral Size	(1024, 1024)

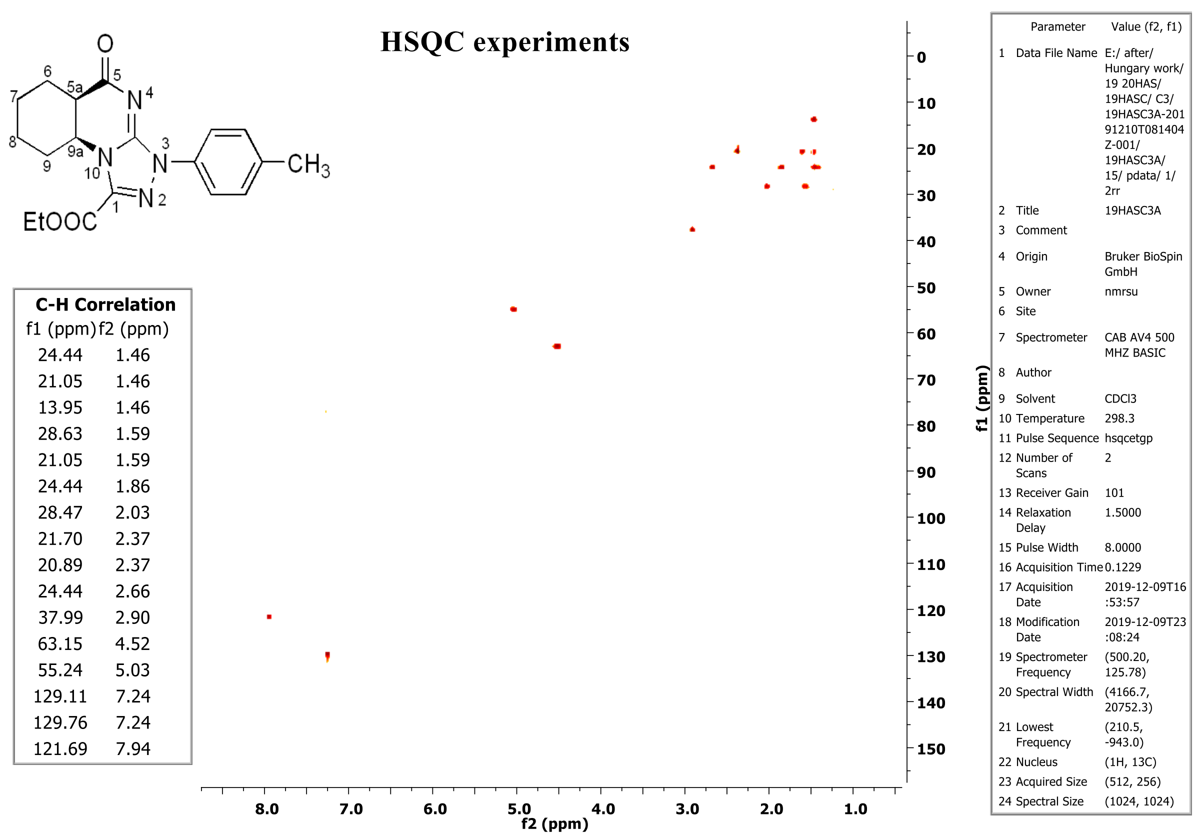

C-H Correlation	
f1 (ppm)	f2 (ppm)
24.44	1.46
21.05	1.46
13.95	1.46
28.63	1.59
21.05	1.59
24.44	1.86
28.47	2.03
21.70	2.37
20.89	2.37
24.44	2.66
37.99	2.90
63.15	4.52
55.24	5.03
129.11	7.24
129.76	7.24
121.69	7.94

	Parameter	Value (f2, f1)
1	Data File Name	E:/ after/ Hungary work/ 19 20HAS/ 19HASC/ C3/ 19HASC3A-20191210T081404Z-001/ 19HASC3A/ 15/ pdata/ 1/ 2rr
2	Title	19HASC3A
3	Comment	
4	Origin	Bruker BioSpin GmbH
5	Owner	nmrsu
6	Site	
7	Spectrometer	CAB AV4 500 MHZ BASIC
8	Author	
9	Solvent	CDCl3
10	Temperature	298.3
11	Pulse Sequence	hsqcetgp
12	Number of Scans	2
13	Receiver Gain	101
14	Relaxation Delay	1.5000
15	Pulse Width	8.0000
16	Acquisition Time	0.1229
17	Acquisition Date	2019-12-09T16:53:57
18	Modification Date	2019-12-09T23:08:24
19	Spectrometer Frequency	(500.20, 125.78)
20	Spectral Width	(4166.7, 20752.3)
21	Lowest Frequency	(210.5, -943.0)
22	Nucleus	(1H, 13C)
23	Acquired Size	(512, 256)
24	Spectral Size	(1024, 1024)

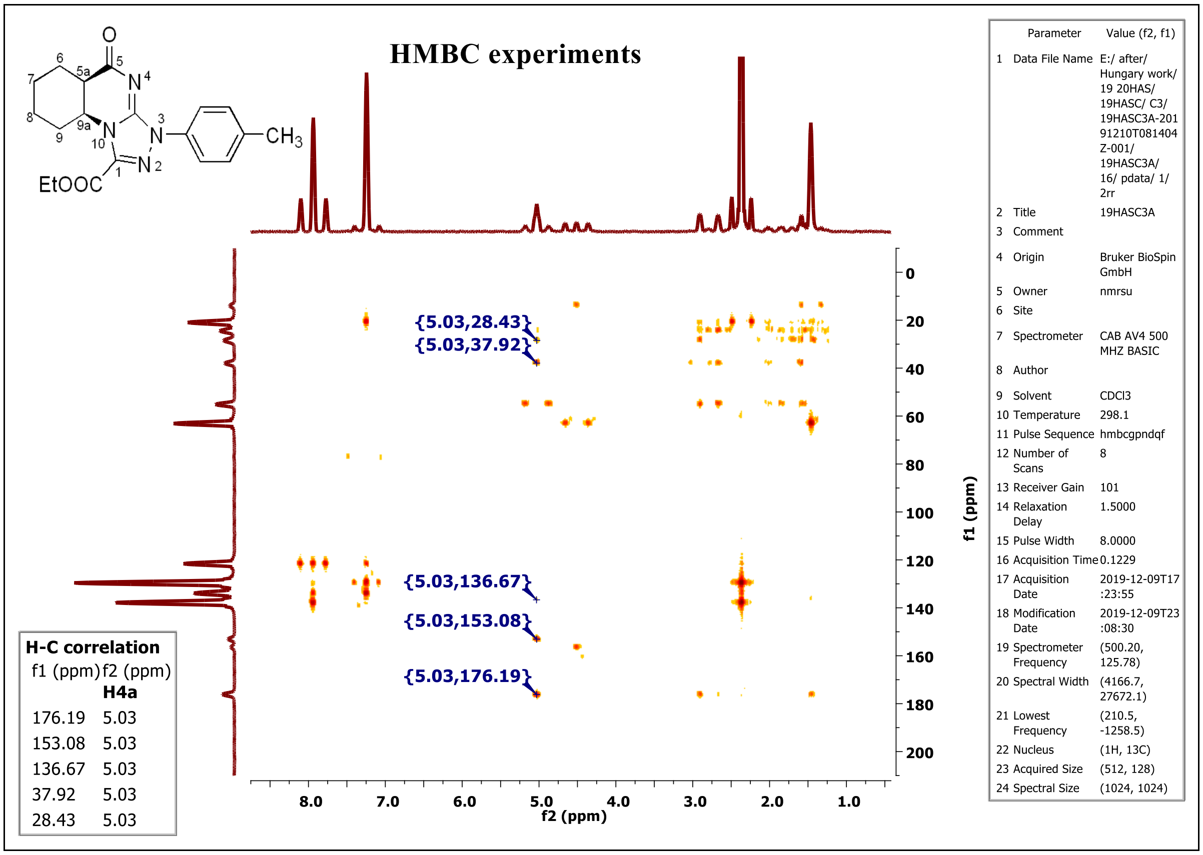
HMBC experiments
EtOOC
CH3
{5.03,28.43}
{5.03,37.92}
{5.03,136.67}
{5.03,153.08}
{5.03,176.19}
H-C correlation
f1 (ppm) f2 (ppm)
H4a
176.19 5.03
153.08 5.03
136.67 5.03
37.92 5.03
28.43 5.03
f2 (ppm)
f1 (ppm)
Parameter Value (f2, f1)
1 Data File Name E:/ after/ Hungary work/ 19 20HAS/ 19HASC/ C3/ 19HASC3A-20191210T081404Z-001/ 19HASC3A/ 16/ pdata/ 1/ 2rr
2 Title 19HASC3A
3 Comment
4 Origin Bruker BioSpin GmbH
5 Owner nmrsu
6 Site
7 Spectrometer CAB AV4 500 MHZ BASIC
8 Author
9 Solvent CDCl3
10 Temperature 298.1
11 Pulse Sequence hmbcgpndqf
12 Number of Scans 8
13 Receiver Gain 101
14 Relaxation Delay 1.5000
15 Pulse Width 8.0000
16 Acquisition Time 0.1229
17 Acquisition Date 2019-12-09T17:23:55
18 Modification Date 2019-12-09T23:08:30
19 Spectrometer Frequency (500.20, 125.78)
20 Spectral Width (4166.7, 27672.1)
21 Lowest Frequency (210.5, -1258.5)
22 Nucleus (1H, 13C)
23 Acquired Size (512, 128)
24 Spectral Size (1024, 1024)

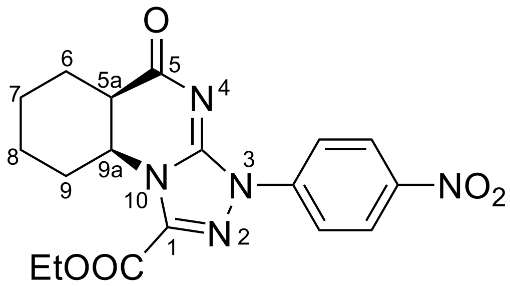

(5a*R,9a*S**)-Ethyl 5-oxo-3-(4-nitrophenyl)-3,5,5a,6,7,8,9,9a-octahydro-[1,2,4]triazolo[4,3-*a*] quinazoline-1-carboxylate (4c)**

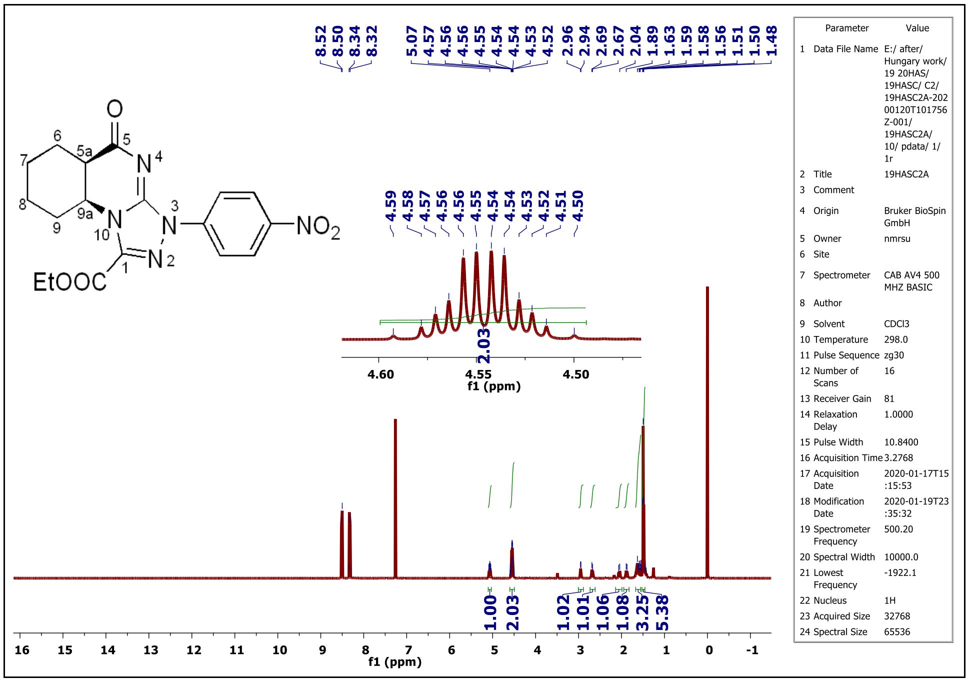

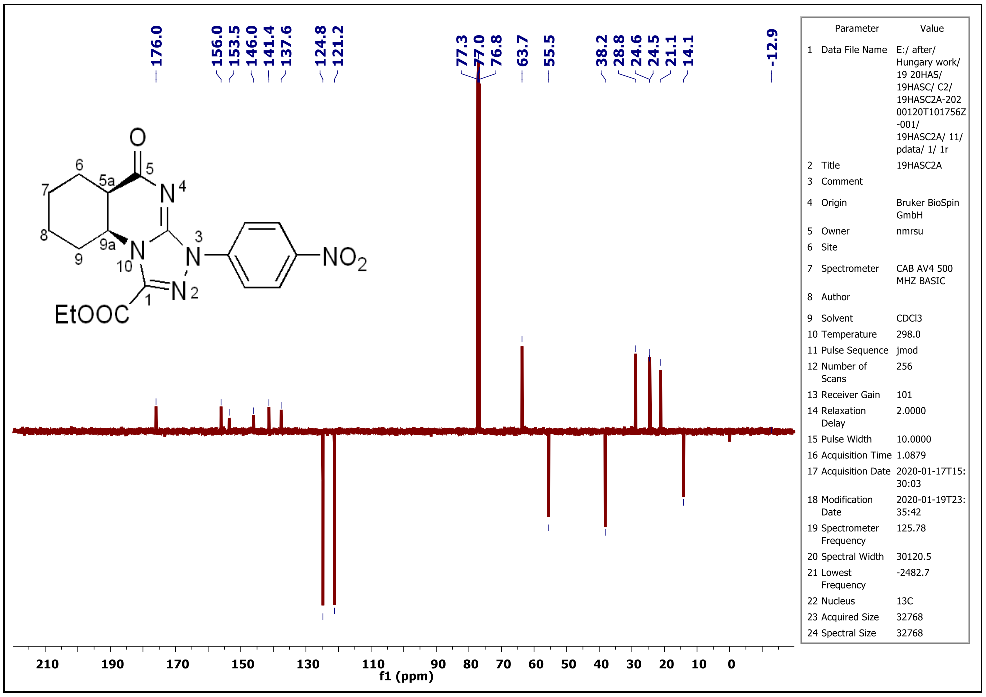

COSY experiments

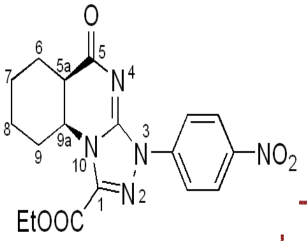

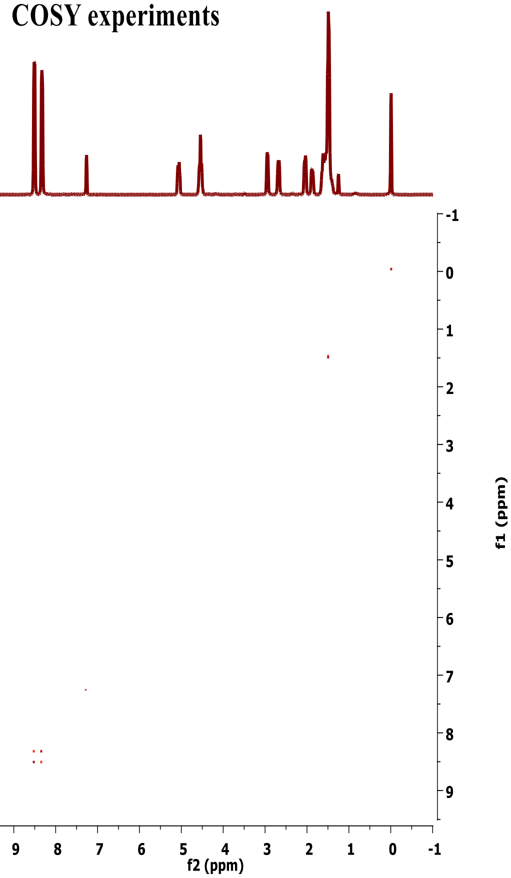

H-H Correlation	
f1 (ppm)	f2 (ppm)
1.47	2.67
1.49	2.68
1.49	4.54
2.94	5.06
8.32	8.51

	Parameter	Value (f2, f1)
1	Data File Name	E:/ after/ Hungary work/ 19 20HAS/ 19HASC/ C2/ 19HASC2A-2020 0120T101756Z-001/ 19HASC2A/ 12/ pdata/ 1/ 2rr
2	Title	19HASC2A
3	Comment	
4	Origin	Bruker BioSpin GmbH
5	Owner	nmrsu
6	Site	
7	Spectrometer	CAB AV4 500 MHZ BASIC
8	Author	
9	Solvent	CDCl3
10	Temperature	298.0
11	Pulse Sequence	cosygpppqf
12	Number of Scans	1
13	Receiver Gain	101
14	Relaxation Delay	1.9611
15	Pulse Width	10.8400
16	Acquisition Time	0.1946
17	Acquisition Date	2020-01-17T15:35:51
18	Modification Date	2020-01-19T23:35:50
19	Spectrometer Frequency	(500.20, 500.20)
20	Spectral Width	(5263.2, 5263.2)
21	Lowest Frequency	(-504.5, -504.5)
22	Nucleus	(1H, 1H)
23	Acquired Size	(1024, 128)
24	Spectral Size	(1024, 1024)

NOESY experiments

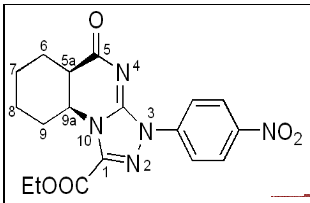

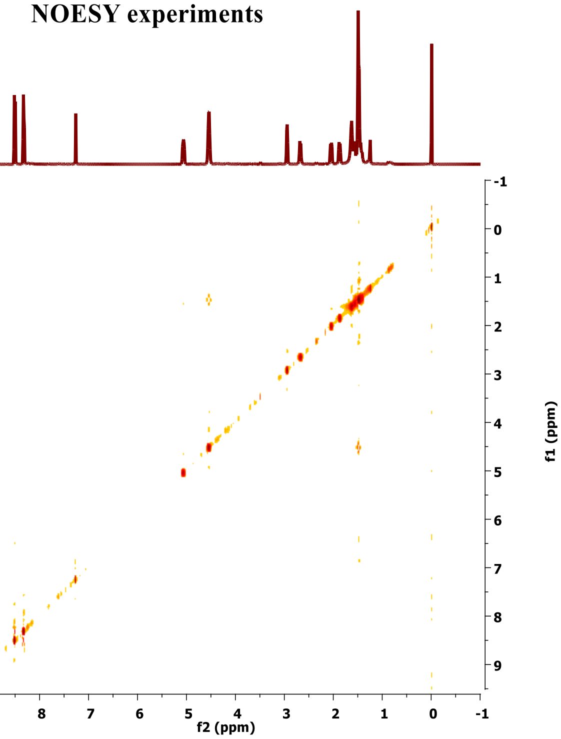

H-H Correlation	
f1 (ppm)	f2 (ppm)
1.41	4.54
1.49	4.58
1.49	4.50
1.57	4.54
1.59	5.06

	Parameter	Value (f2, f1)
1	Data File Name	E:/ after/ Hungary work/ 19 20HAS/ 19HASC/ C2/ 19HASC2A-202 00120T101756 Z-001/ 19HASC2A/ 13/ pdata/ 1/ 2rr
2	Title	19HASC2A
3	Comment	
4	Origin	Bruker BioSpin GmbH
5	Owner	nmrsu
6	Site	
7	Spectrometer	CAB AV4 500 MHZ BASIC
8	Author	
9	Solvent	CDCl3
10	Temperature	298.0
11	Pulse Sequence	noesygpphpp
12	Number of Scans	4
13	Receiver Gain	101
14	Relaxation Delay	2.0102
15	Pulse Width	10.8400
16	Acquisition Time	0.1946
17	Acquisition Date	2020-01-17T16:21:21
18	Modification Date	2020-01-19T23:35:58
19	Spectrometer Frequency	(500.20, 500.20)
20	Spectral Width	(5263.2, 5263.2)
21	Lowest Frequency	(-504.5, -504.5)
22	Nucleus	(1H, 1H)
23	Acquired Size	(1024, 256)
24	Spectral Size	(1024, 1024)

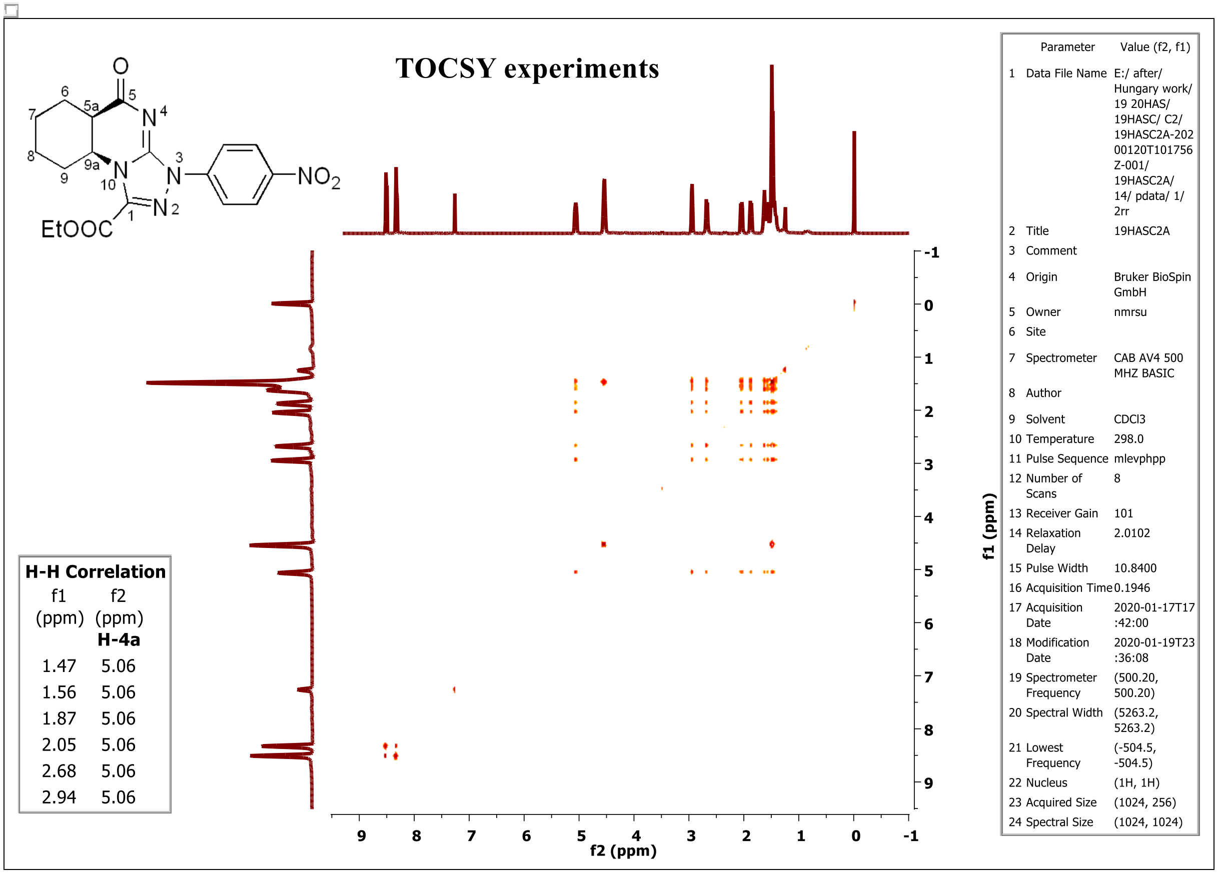
TOCSY experiments
H-H Correlation
f1 (ppm) f2 (ppm)
H-4a
1.47 5.06
1.56 5.06
1.87 5.06
2.05 5.06
2.68 5.06
2.94 5.06
EtOOC
NO_2
f2 (ppm)
f1 (ppm)
Parameter Value (f2, f1)
1 Data File Name E:/ after/ Hungary work/ 19 20HAS/ 19HASC/ C2/ 19HASC2A-20200120T101756Z-001/ 19HASC2A/ 14/ pdata/ 1/ 2rr
2 Title 19HASC2A
3 Comment
4 Origin Bruker BioSpin GmbH
5 Owner nmrsu
6 Site
7 Spectrometer CAB AV4 500 MHZ BASIC
8 Author
9 Solvent CDCl3
10 Temperature 298.0
11 Pulse Sequence mlevphpp
12 Number of Scans 8
13 Receiver Gain 101
14 Relaxation Delay 2.0102
15 Pulse Width 10.8400
16 Acquisition Time 0.1946
17 Acquisition Date 2020-01-17T17:42:00
18 Modification Date 2020-01-19T23:36:08
19 Spectrometer Frequency (500.20, 500.20)
20 Spectral Width (5263.2, 5263.2)
21 Lowest Frequency (-504.5, -504.5)
22 Nucleus (1H, 1H)
23 Acquired Size (1024, 256)
24 Spectral Size (1024, 1024)

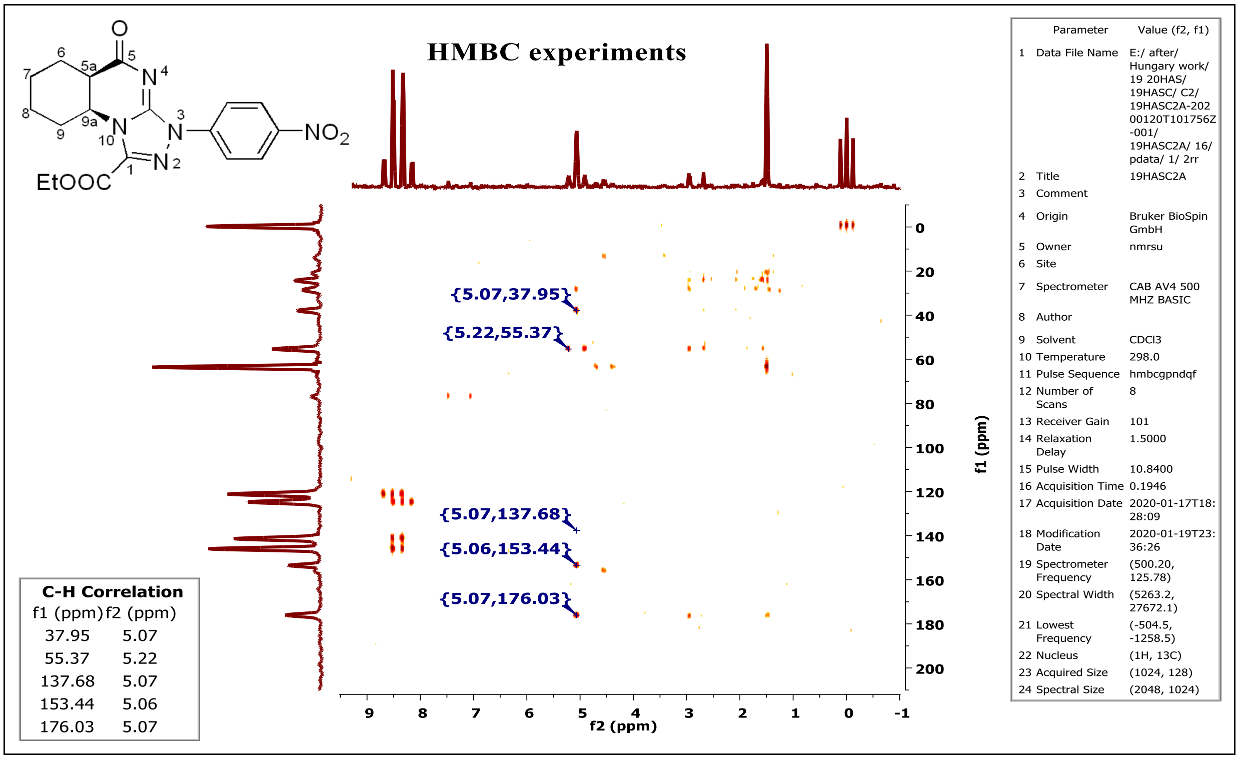
HMBC experiments
{5.07,37.95}
{5.22,55.37}
{5.07,137.68}
{5.06,153.44}
{5.07,176.03}
C-H Correlation
f1 (ppm) f2 (ppm)
37.95 5.07
55.37 5.22
137.68 5.07
153.44 5.06
176.03 5.07
EtOOC
NO_2
f2 (ppm)
f1 (ppm)
Parameter Value (f2, f1)
1 Data File Name E:/ after/ Hungary work/ 19 20HAS/ 19HASC/ C2/ 19HASC2A-20200120T101756Z-001/ 19HASC2A/ 16/ pdata/ 1/ 2rr
2 Title 19HASC2A
3 Comment
4 Origin Bruker BioSpin GmbH
5 Owner nmrsu
6 Site
7 Spectrometer CAB AV4 500 MHZ BASIC
8 Author
9 Solvent CDCl3
10 Temperature 298.0
11 Pulse Sequence hmbcgpndqf
12 Number of Scans 8
13 Receiver Gain 101
14 Relaxation Delay 1.5000
15 Pulse Width 10.8400
16 Acquisition Time 0.1946
17 Acquisition Date 2020-01-17T18:28:09
18 Modification Date 2020-01-19T23:36:26
19 Spectrometer Frequency (500.20, 125.78)
20 Spectral Width (5263.2, 27672.1)
21 Lowest Frequency (-504.5, -1258.5)
22 Nucleus (1H, 13C)
23 Acquired Size (1024, 128)
24 Spectral Size (2048, 1024)

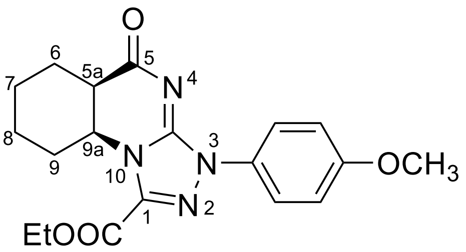

(5a*R,9a*S**)-Ethyl 5-oxo-3-(4-methoxyphenyl)-3,5,5a,6,7,8,9,9a-octahydro-[1,2,4]triazolo[4,3-*a*]quinazoline-1-carboxylate (4d)**

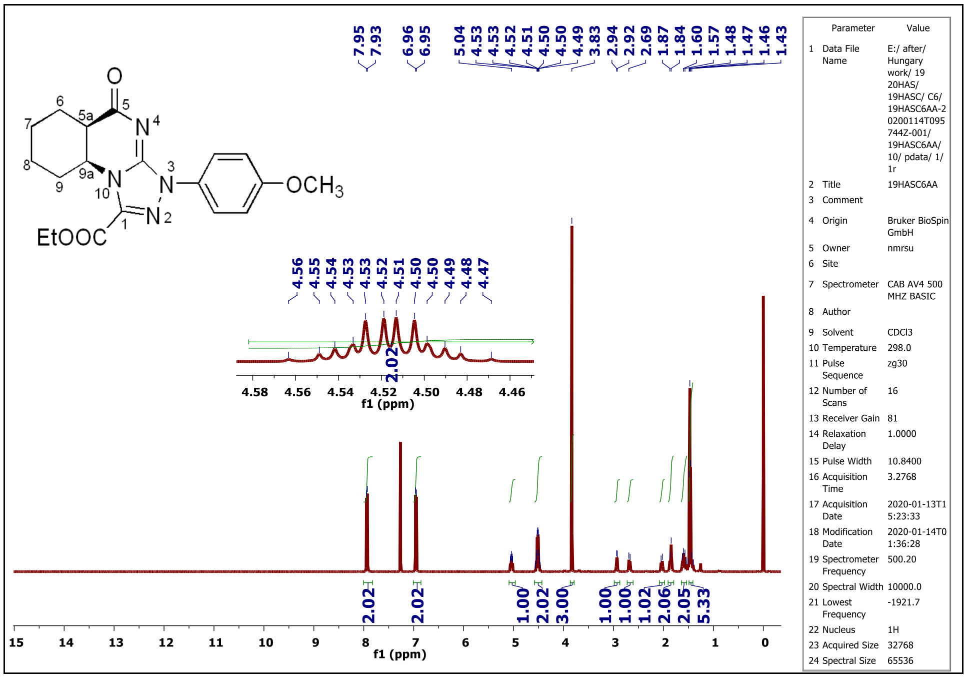

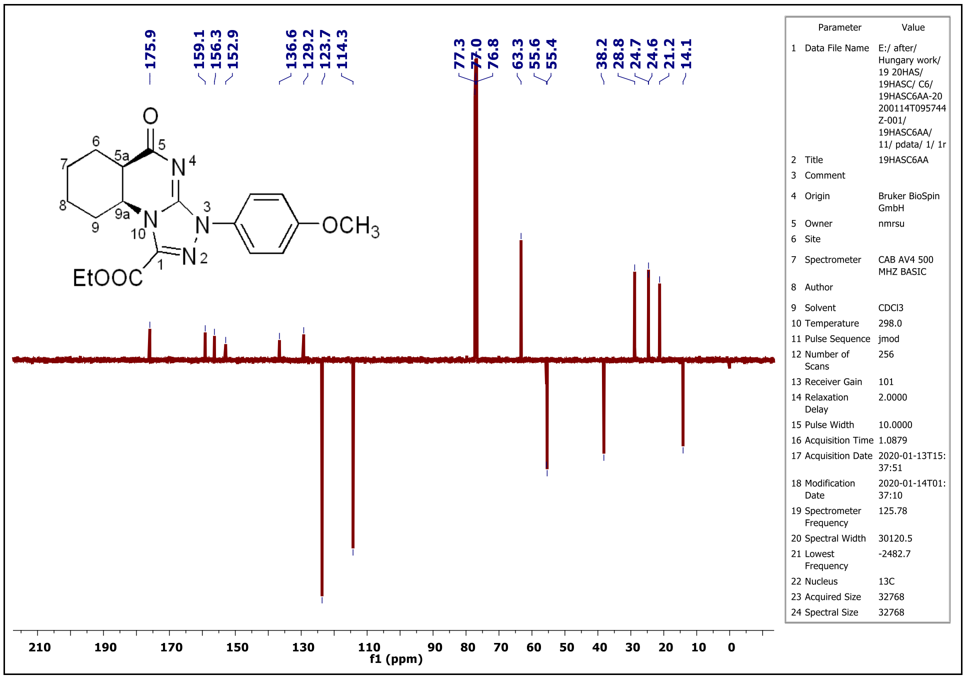

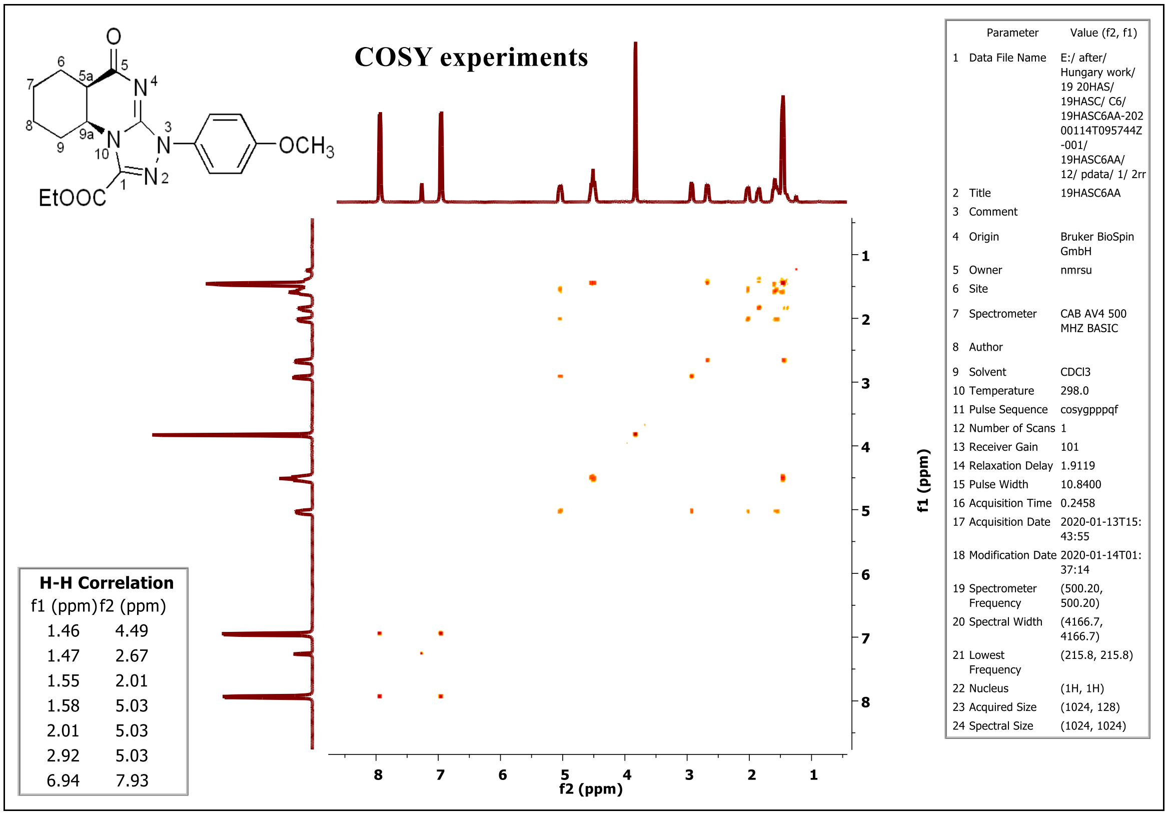

H-H Correlation

f1 (ppm)	f2 (ppm)
1.46	4.49
1.47	2.67
1.55	2.01
1.58	5.03
2.01	5.03
2.92	5.03
6.94	7.93

	Parameter	Value (f2, f1)
1	Data File Name	E:/ after/ Hungary work/ 19 20HAS/ 19HASC/ C6/ 19HASC6AA-20200114T095744Z-001/ 19HASC6AA/ 12/ pdata/ 1/ 2rr
2	Title	19HASC6AA
3	Comment	
4	Origin	Bruker BioSpin GmbH
5	Owner	nmrsu
6	Site	
7	Spectrometer	CAB AV4 500 MHZ BASIC
8	Author	
9	Solvent	CDCl3
10	Temperature	298.0
11	Pulse Sequence	cosygpppqf
12	Number of Scans	1
13	Receiver Gain	101
14	Relaxation Delay	1.9119
15	Pulse Width	10.8400
16	Acquisition Time	0.2458
17	Acquisition Date	2020-01-13T15:43:55
18	Modification Date	2020-01-14T01:37:14
19	Spectrometer Frequency	(500.20, 500.20)
20	Spectral Width	(4166.7, 4166.7)
21	Lowest Frequency	(215.8, 215.8)
22	Nucleus	(1H, 1H)
23	Acquired Size	(1024, 128)
24	Spectral Size	(1024, 1024)

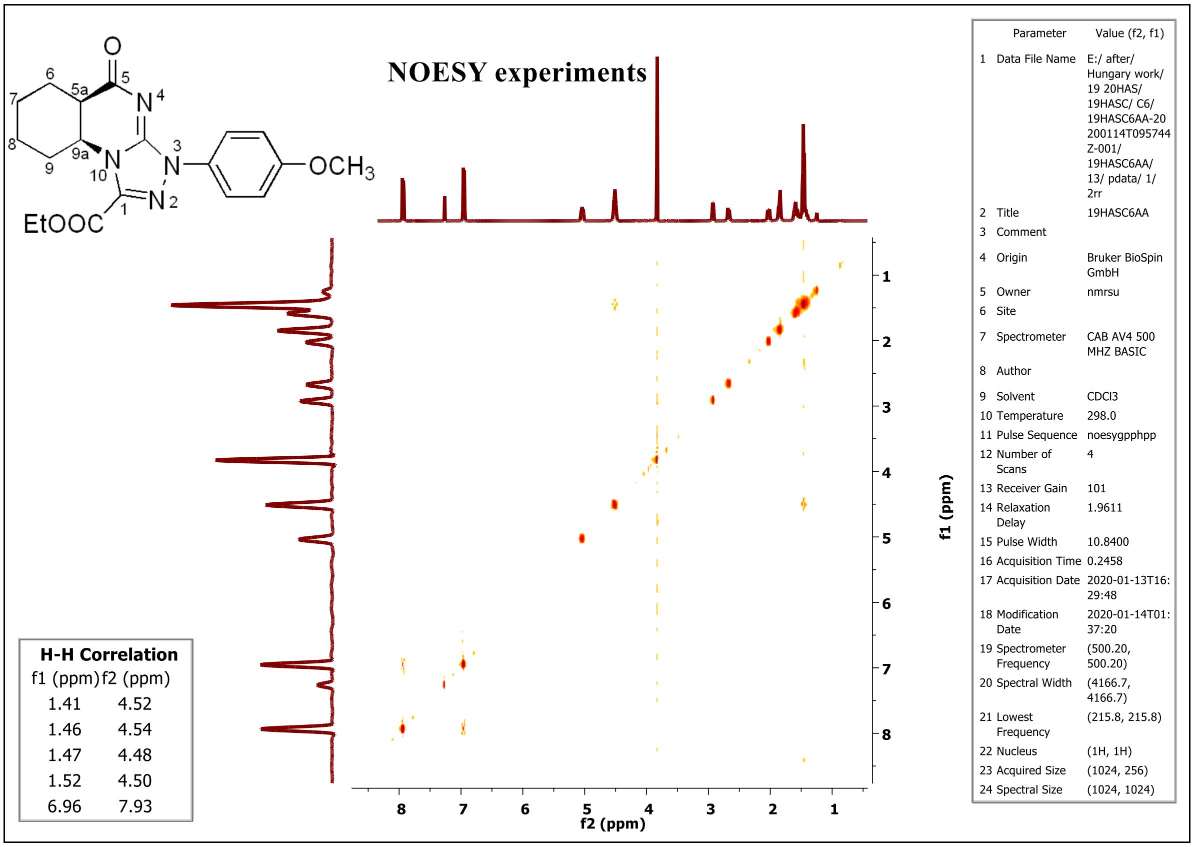

H-H Correlation

f1 (ppm)	f2 (ppm)
1.41	4.52
1.46	4.54
1.47	4.48
1.52	4.50
6.96	7.93

	Parameter	Value (f2, f1)
1	Data File Name	E:/ after/ Hungary work/ 19 20HAS/ 19HASC/ C6/ 19HASC6AA-20200114T095744Z-001/ 19HASC6AA/ 13/ pdata/ 1/ 2rr
2	Title	19HASC6AA
3	Comment	
4	Origin	Bruker BioSpin GmbH
5	Owner	nmrsu
6	Site	
7	Spectrometer	CAB AV4 500 MHZ BASIC
8	Author	
9	Solvent	CDCl3
10	Temperature	298.0
11	Pulse Sequence	noesygpphpp
12	Number of Scans	4
13	Receiver Gain	101
14	Relaxation Delay	1.9611
15	Pulse Width	10.8400
16	Acquisition Time	0.2458
17	Acquisition Date	2020-01-13T16:29:48
18	Modification Date	2020-01-14T01:37:20
19	Spectrometer Frequency	(500.20, 500.20)
20	Spectral Width	(4166.7, 4166.7)
21	Lowest Frequency	(215.8, 215.8)
22	Nucleus	(1H, 1H)
23	Acquired Size	(1024, 256)
24	Spectral Size	(1024, 1024)

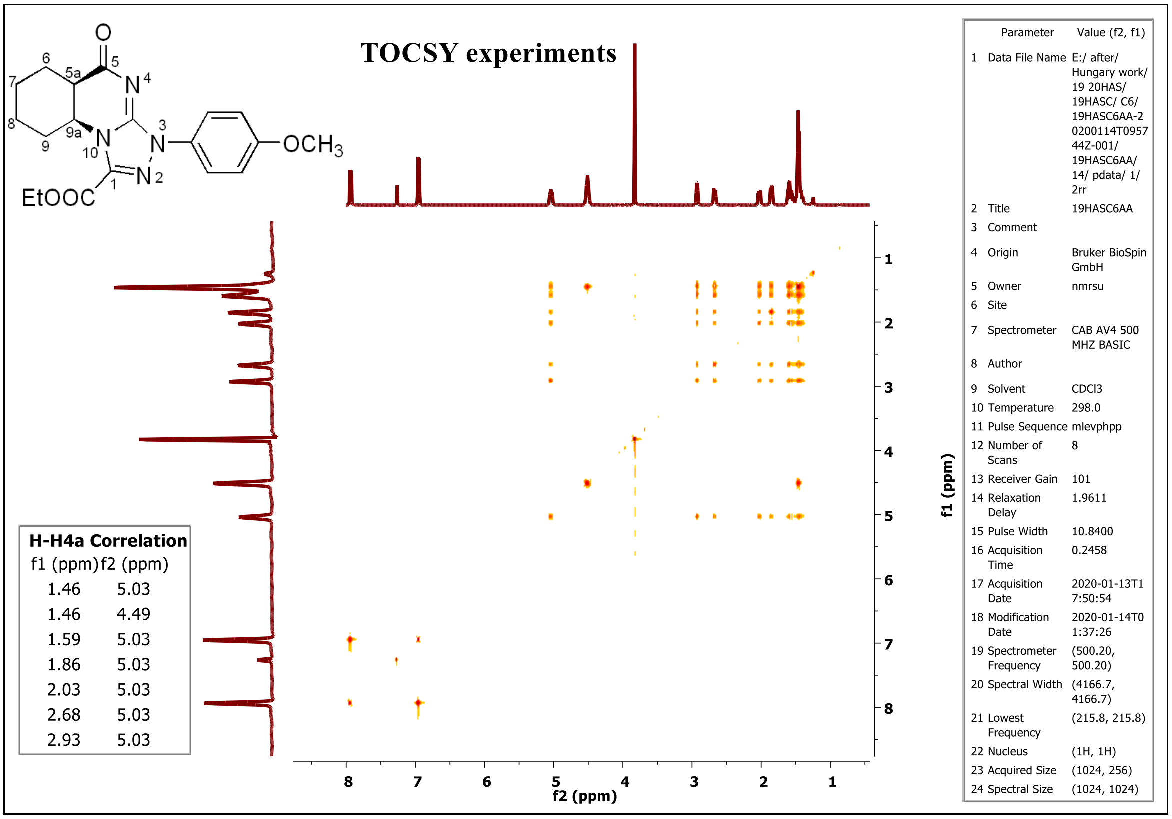
TOCSY experiments
EtOOC
OCH3
H-H4a Correlation
f1 (ppm) f2 (ppm)
1.46 5.03
1.46 4.49
1.59 5.03
1.86 5.03
2.03 5.03
2.68 5.03
2.93 5.03
f2 (ppm)
f1 (ppm)
Parameter Value (f2, f1)
1 Data File Name E:/ after/ Hungary work/ 19 20HAS/ 19HASC/ C6/ 19HASC6AA-2 0200114T0957 44Z-001/ 19HASC6AA/ 14/ pdata/ 1/ 2rr
2 Title 19HASC6AA
3 Comment
4 Origin Bruker BioSpin GmbH
5 Owner nmrsu
6 Site
7 Spectrometer CAB AV4 500 MHZ BASIC
8 Author
9 Solvent CDCl3
10 Temperature 298.0
11 Pulse Sequence mlevphpp
12 Number of Scans 8
13 Receiver Gain 101
14 Relaxation Delay 1.9611
15 Pulse Width 10.8400
16 Acquisition Time 0.2458
17 Acquisition Date 2020-01-13T17:50:54
18 Modification Date 2020-01-14T01:37:26
19 Spectrometer Frequency (500.20, 500.20)
20 Spectral Width (4166.7, 4166.7)
21 Lowest Frequency (215.8, 215.8)
22 Nucleus (1H, 1H)
23 Acquired Size (1024, 256)
24 Spectral Size (1024, 1024)

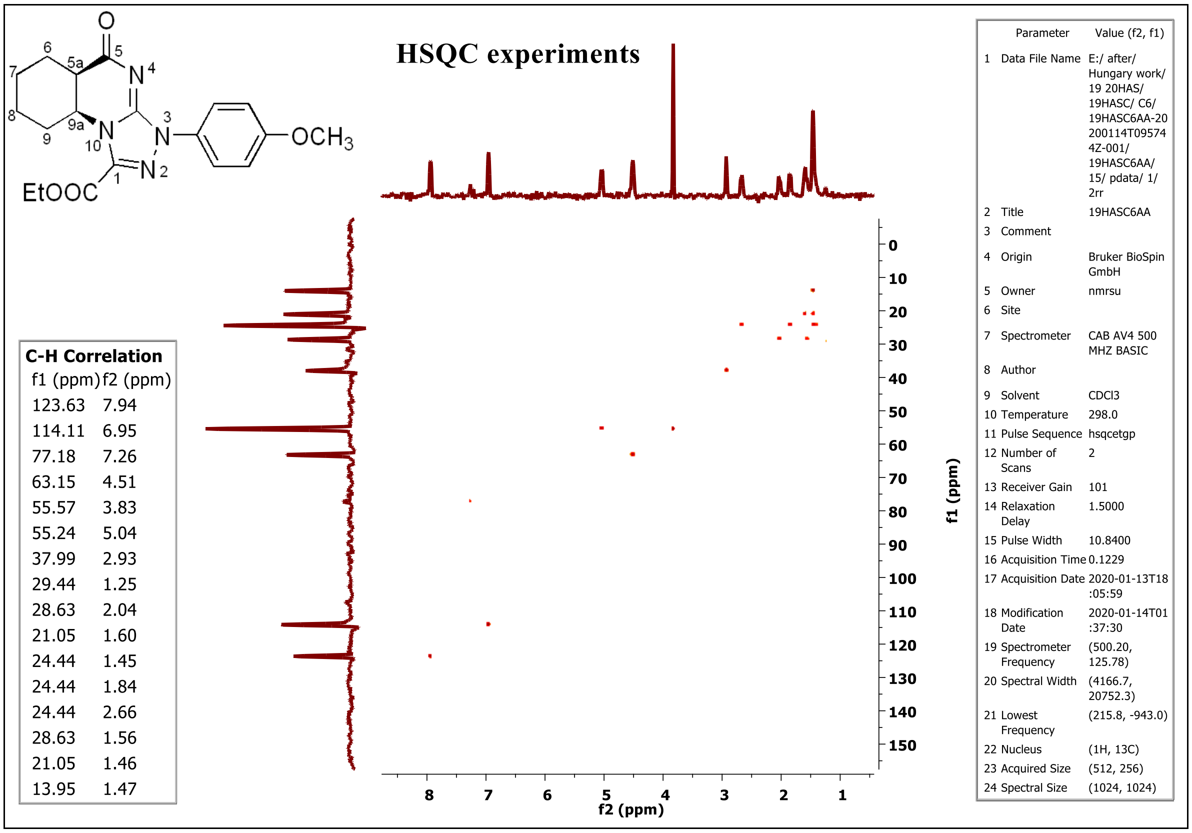
HSQC experiments
EtOOC
OCH3
C-H Correlation
f1 (ppm) f2 (ppm)
123.63 7.94
114.11 6.95
77.18 7.26
63.15 4.51
55.57 3.83
55.24 5.04
37.99 2.93
29.44 1.25
28.63 2.04
21.05 1.60
24.44 1.45
24.44 1.84
24.44 2.66
28.63 1.56
21.05 1.46
13.95 1.47
f2 (ppm)
f1 (ppm)
Parameter Value (f2, f1)
1 Data File Name E:/ after/ Hungary work/ 19 20HAS/ 19HASC/ C6/ 19HASC6AA-20 200114T09574 4Z-001/ 19HASC6AA/ 15/ pdata/ 1/ 2rr
2 Title 19HASC6AA
3 Comment
4 Origin Bruker BioSpin GmbH
5 Owner nmrsu
6 Site
7 Spectrometer CAB AV4 500 MHZ BASIC
8 Author
9 Solvent CDCl3
10 Temperature 298.0
11 Pulse Sequence hsqcetgp
12 Number of Scans 2
13 Receiver Gain 101
14 Relaxation Delay 1.5000
15 Pulse Width 10.8400
16 Acquisition Time 0.1229
17 Acquisition Date 2020-01-13T18:05:59
18 Modification Date 2020-01-14T01:37:30
19 Spectrometer Frequency (500.20, 125.78)
20 Spectral Width (4166.7, 20752.3)
21 Lowest Frequency (215.8, -943.0)
22 Nucleus (1H, 13C)
23 Acquired Size (512, 256)
24 Spectral Size (1024, 1024)

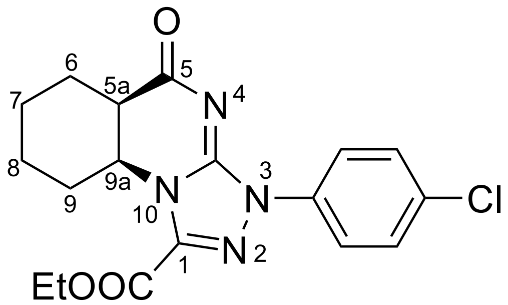

(5a*R,9a*S**)-Ethyl 5-oxo-3-(4-chlorophenyl)-3,5,5a,6,7,8,9,9a-octahydro-[1,2,4]triazolo[4,3-*a*] quinazoline-1-carboxylate (4e)**

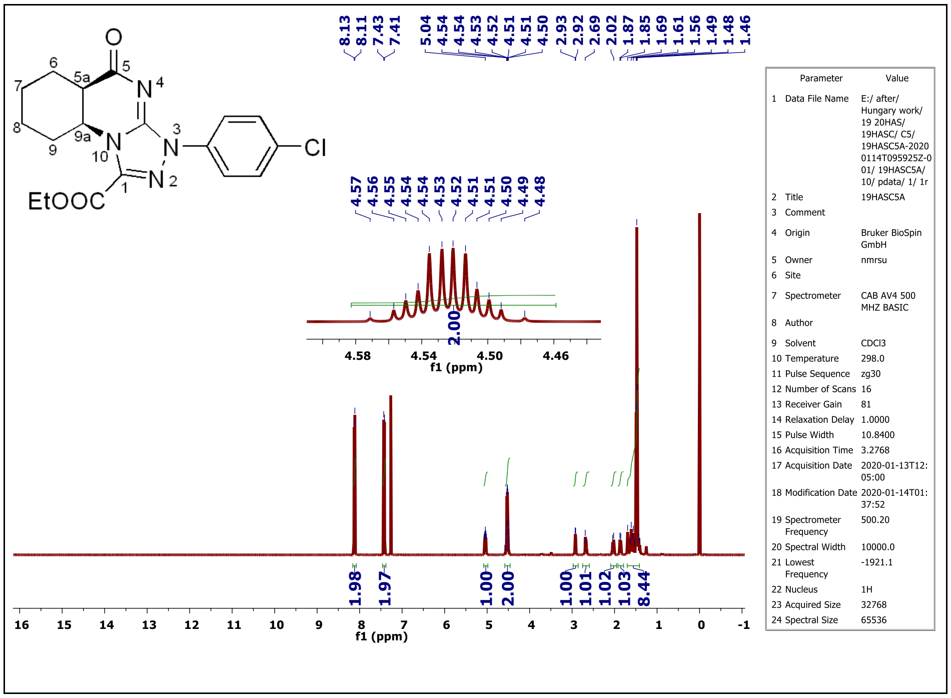

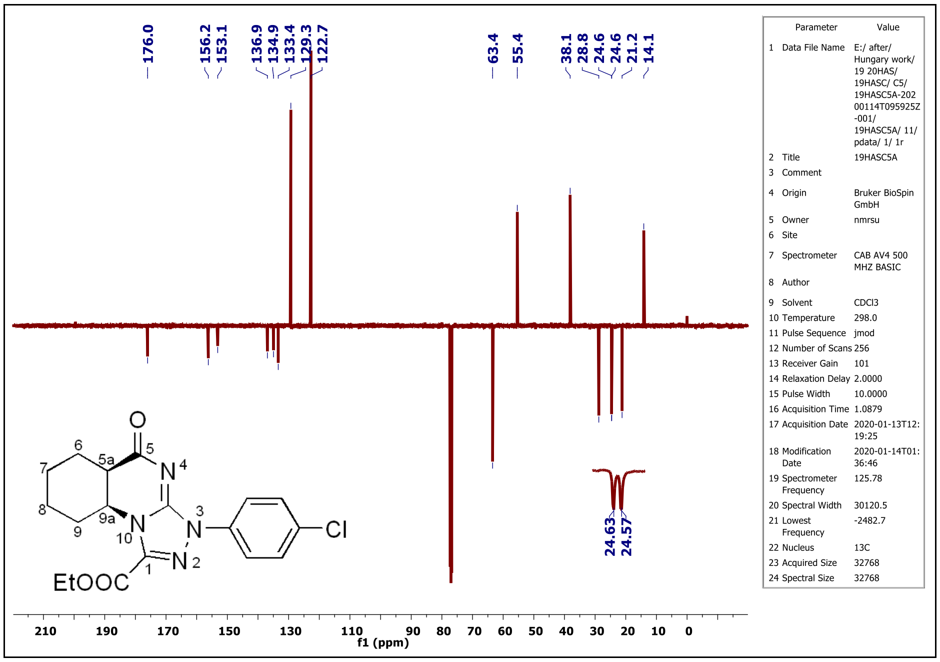

COSY experiments

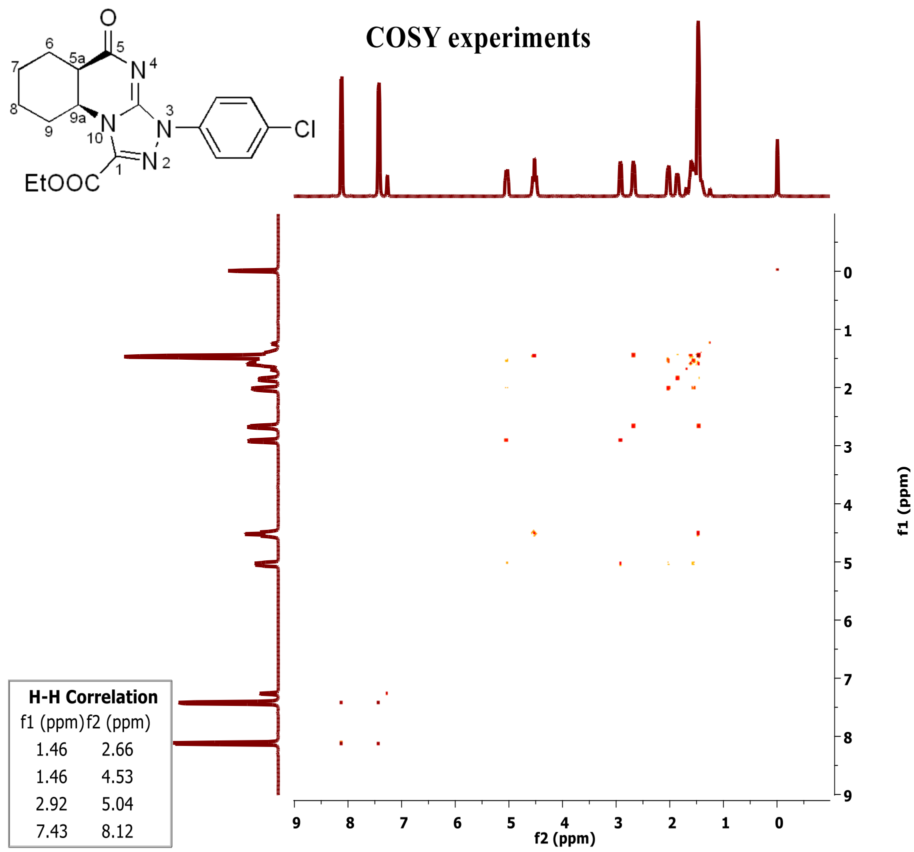

H-H Correlation	
f1 (ppm)	f2 (ppm)
1.46	2.66
1.46	4.53
2.92	5.04
7.43	8.12

	Parameter	Value (f2, f1)
1	Data File Name	E:/ after/ Hungary work/ 19 20HAS/ 19HASC/ C5/ 19HASC5A-20 200114T09592 5Z-001/ 19HASC5A/ 12/ pdata/ 1/ 2rr
2	Title	19HASC5A
3	Comment	
4	Origin	Bruker BioSpin GmbH
5	Owner	nmrsu
6	Site	
7	Spectrometer	CAB AV4 500 MHZ BASIC
8	Author	
9	Solvent	CDCl3
10	Temperature	298.0
11	Pulse Sequence	cosygpppqf
12	Number of Scans	1
13	Receiver Gain	101
14	Relaxation Delay	1.9549
15	Pulse Width	10.8400
16	Acquisition Time	0.2048
17	Acquisition Date	2020-01-13T12:25:15
18	Modification Date	2020-01-14T01:36:52
19	Spectrometer Frequency	(500.20, 500.20)
20	Spectral Width	(5000.0, 5000.0)
21	Lowest Frequency	(-492.8, -492.8)
22	Nucleus	(1H, 1H)
23	Acquired Size	(1024, 128)
24	Spectral Size	(1024, 1024)

NOESY experiments

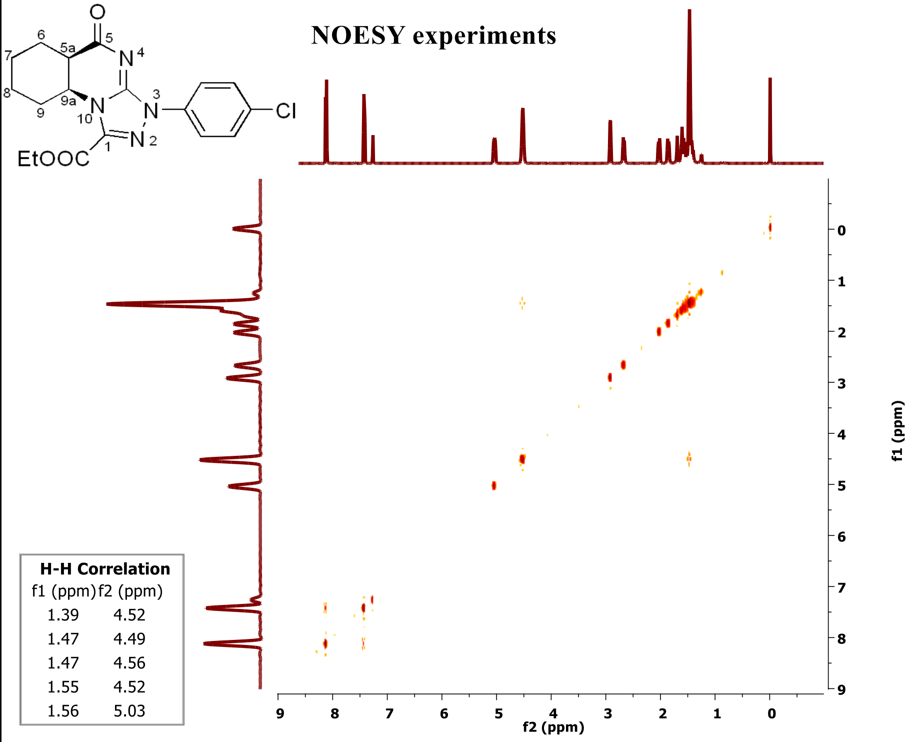

H-H Correlation	
f1 (ppm)	f2 (ppm)
1.39	4.52
1.47	4.49
1.47	4.56
1.55	4.52
1.56	5.03

	Parameter	Value (f2, f1)
1	Data File Name	E:/ after/ Hungary work/ 19 20HAS/ 19HASC/ C5/ 19HASC5A-20 200114T0959 25Z-001/ 19HASC5A/ 13/ pdata/ 1/ 2rr
2	Title	19HASC5A
3	Comment	
4	Origin	Bruker BioSpin GmbH
5	Owner	nmrsu
6	Site	
7	Spectrometer	CAB AV4 500 MHZ BASIC
8	Author	
9	Solvent	CDCl3
10	Temperature	298.0
11	Pulse Sequence	noesygpphpp
12	Number of Scans	4
13	Receiver Gain	101
14	Relaxation Delay	2.0041
15	Pulse Width	10.8400
16	Acquisition Time	0.2048
17	Acquisition Date	2020-01-13T13:10:51
18	Modification Date	2020-01-14T01:36:58
19	Spectrometer Frequency	(500.20, 500.20)
20	Spectral Width	(5000.0, 5000.0)
21	Lowest Frequency	(-492.8, -492.8)
22	Nucleus	(1H, 1H)
23	Acquired Size	(1024, 256)
24	Spectral Size	(1024, 1024)

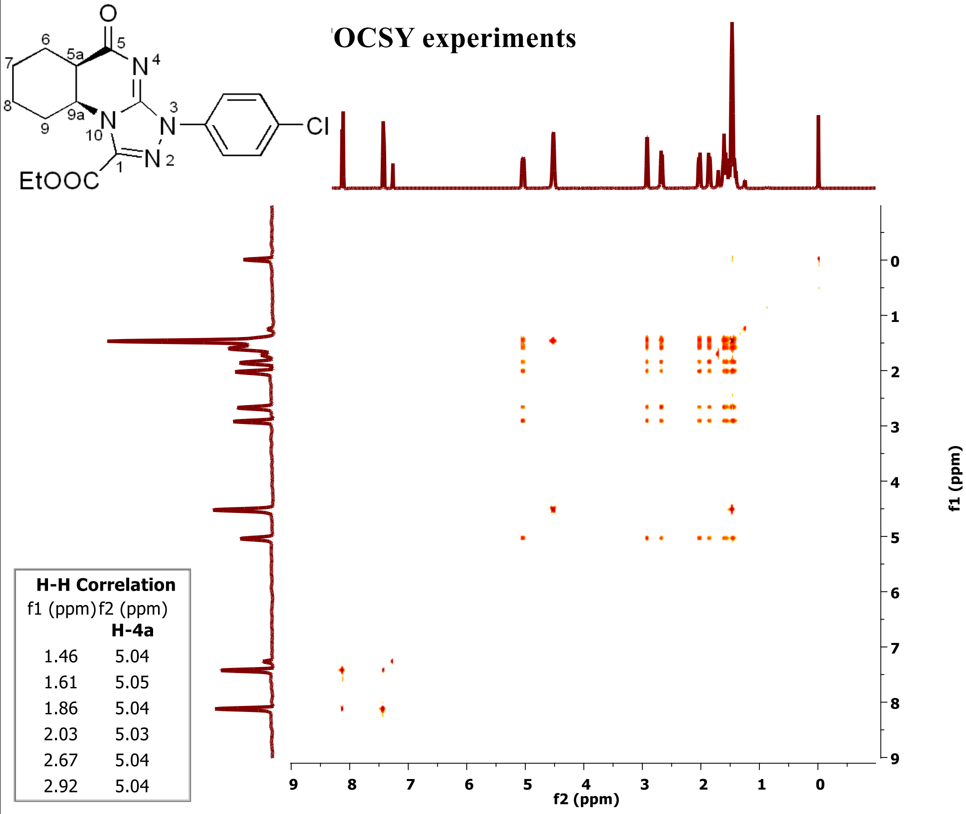

H-H Correlation

f1 (ppm)	f2 (ppm)
	H-4a
1.46	5.04
1.61	5.05
1.86	5.04
2.03	5.03
2.67	5.04
2.92	5.04

	Parameter	Value (f2, f1)
1	Data File Name	E:/ after/ Hungary work/ 19 20HAS/ 19HASC/ C5/ 19HASC5A-202 00114T095925 Z-001/ 19HASC5A/ 14/ pdata/ 1/ 2rr
2	Title	19HASC5A
3	Comment	
4	Origin	Bruker BioSpin GmbH
5	Owner	nmrsu
6	Site	
7	Spectrometer	CAB AV4 500 MHZ BASIC
8	Author	
9	Solvent	CDCl3
10	Temperature	298.0
11	Pulse Sequence	mlevphpp
12	Number of Scans	8
13	Receiver Gain	101
14	Relaxation Delay	2.0041
15	Pulse Width	10.8400
16	Acquisition Time	0.2048
17	Acquisition Date	2020-01-13T14:31:41
18	Modification Date	2020-01-14T01:37:02
19	Spectrometer Frequency	(500.20, 500.20)
20	Spectral Width	(5000.0, 5000.0)
21	Lowest Frequency	(-492.8, -492.8)
22	Nucleus	(1H, 1H)
23	Acquired Size	(1024, 256)
24	Spectral Size	(1024, 1024)

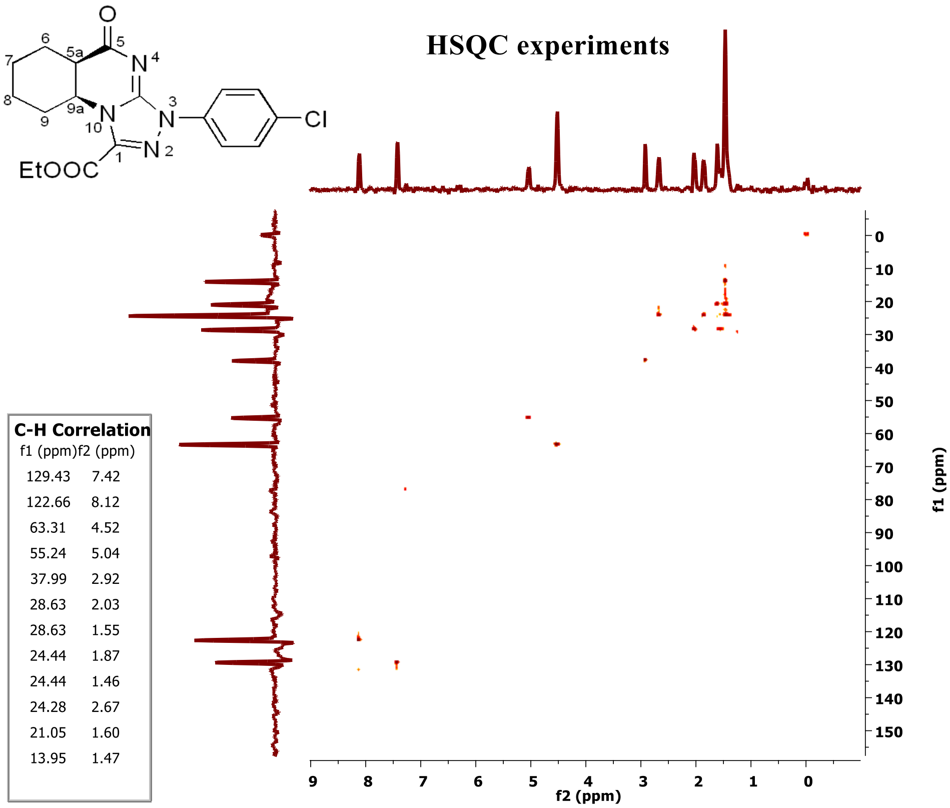

C-H Correlation

f1 (ppm)	f2 (ppm)
129.43	7.42
122.66	8.12
63.31	4.52
55.24	5.04
37.99	2.92
28.63	2.03
28.63	1.55
24.44	1.87
24.44	1.46
24.28	2.67
21.05	1.60
13.95	1.47

	Parameter	Value (f2, f1)
1	Data File Name	E:/ after/ Hungary work/ 19 20HAS/ 19HASC/ C5/ 19HASC5A-202 00114T095925Z -001/ 19HASC5A/ 15/ pdata/ 1/ 2rr
2	Title	19HASC5A
3	Comment	
4	Origin	Bruker BioSpin GmbH
5	Owner	nmrsu
6	Site	
7	Spectrometer	CAB AV4 500 MHZ BASIC
8	Author	
9	Solvent	CDCl3
10	Temperature	298.0
11	Pulse Sequence	hsqcetgp
12	Number of Scans	2
13	Receiver Gain	101
14	Relaxation Delay	1.5000
15	Pulse Width	10.8400
16	Acquisition Time	0.1024
17	Acquisition Date	2020-01-13T14:46:49
18	Modification Date	2020-01-14T01:38:06
19	Spectrometer Frequency	(500.20, 125.78)
20	Spectral Width	(5000.0, 20752.3)
21	Lowest Frequency	(-492.8, -943.0)
22	Nucleus	(1H, 13C)
23	Acquired Size	(512, 256)
24	Spectral Size	(1024, 1024)

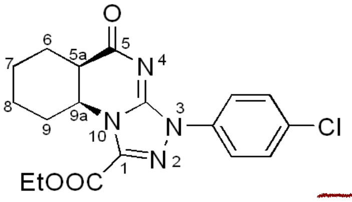

HMBC experiments

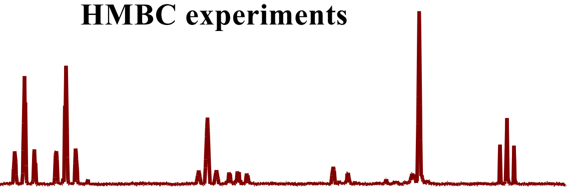

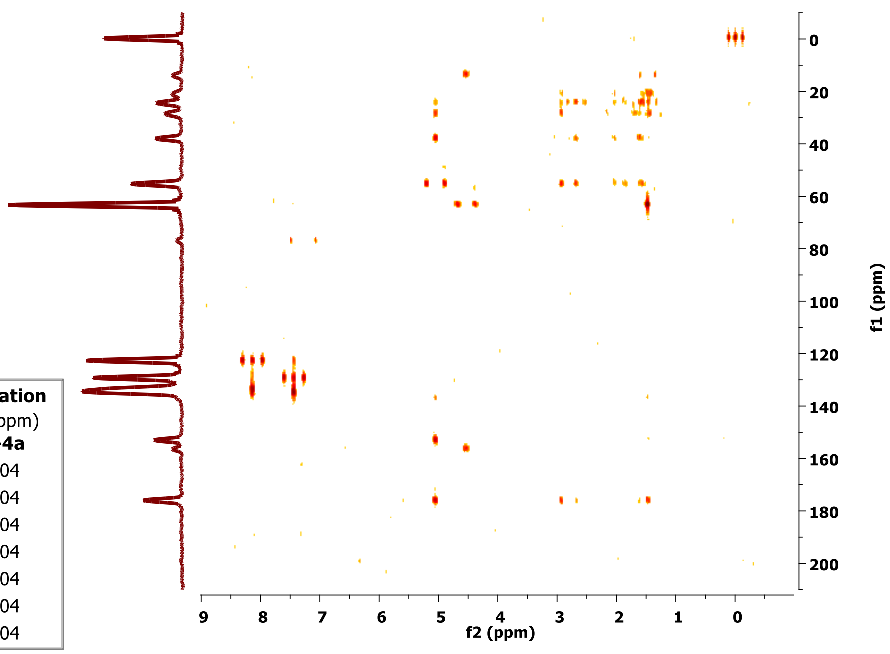

C-H Correlation

f1 (ppm)	f2 (ppm)
	**H-4a**
176.08	5.04
152.86	5.04
137.00	5.04
55.76	5.04
37.97	5.04
28.56	5.04
24.44	5.04

	Parameter	Value (f2, f1)
1	Data File Name	E:/ after/ Hungary work/ 19 20HAS/ 19HASC/ C5/ 19HASC5A-20200114T095925Z-001/ 19HASC5A/ 16/ pdata/ 1/ 2rr
2	Title	19HASC5A
3	Comment	
4	Origin	Bruker BioSpin GmbH
5	Owner	nmrsu
6	Site	
7	Spectrometer	CAB AV4 500 MHZ BASIC
8	Author	
9	Solvent	CDCl3
10	Temperature	298.0
11	Pulse Sequence	hmbcgpndqf
12	Number of Scans	8
13	Receiver Gain	101
14	Relaxation Delay	1.5000
15	Pulse Width	10.8400
16	Acquisition Time	0.2048
17	Acquisition Date	2020-01-13T15:18:31
18	Modification Date	2020-01-14T01:38:10
19	Spectrometer Frequency	(500.20, 125.78)
20	Spectral Width	(5000.0, 27672.1)
21	Lowest Frequency	(-492.8, -1258.5)
22	Nucleus	(1H, 13C)
23	Acquired Size	(1024, 128)
24	Spectral Size	(2048, 1024)

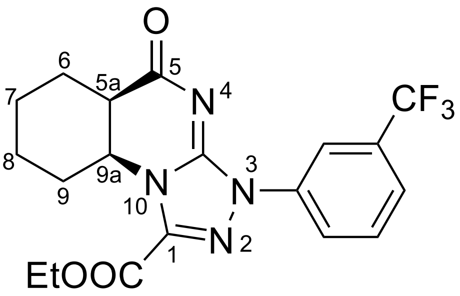

(5a*R,9a*S**)-Ethyl 5-oxo-3-(4-(trifluoromethyl)phenyl)-3,5,5a,6,7,8,9,9a-octahydro-[1,2,4]triazolo[4,3-*a*]quinazoline-1-carboxylate (4f)**

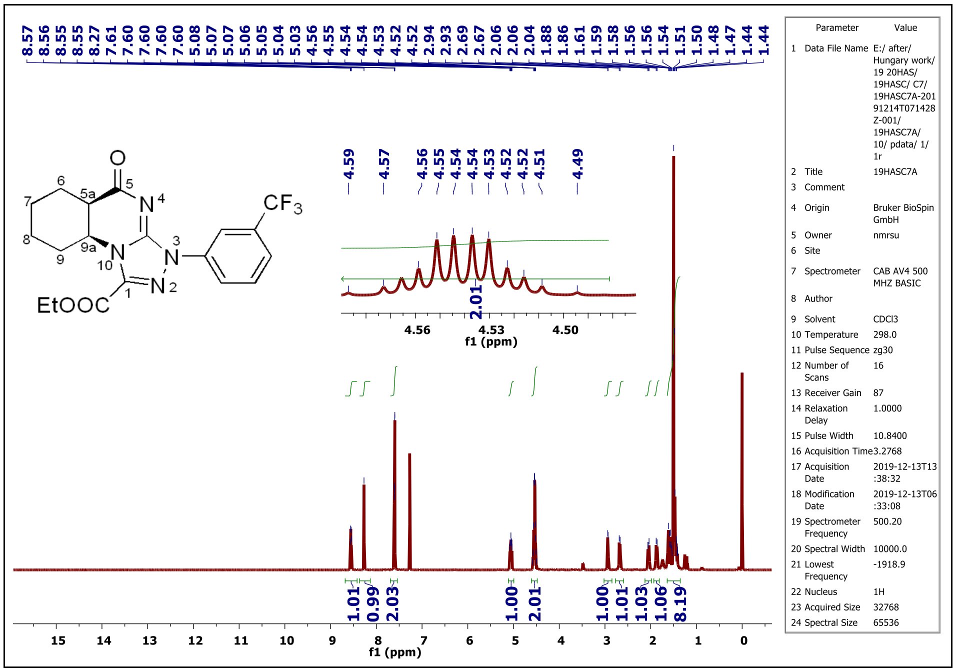

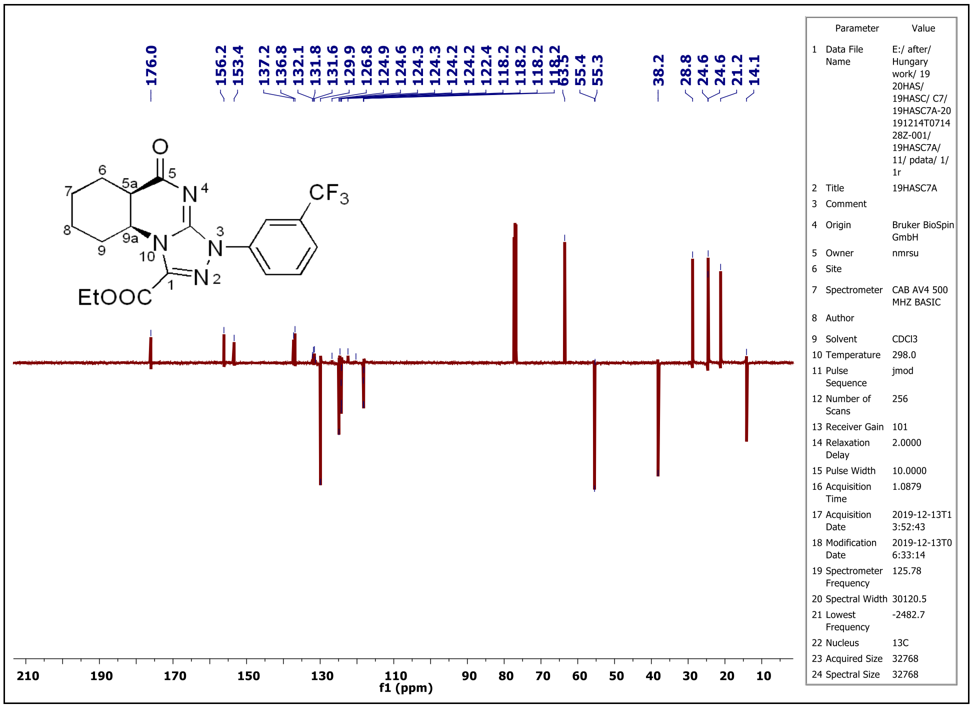

COSY experiments

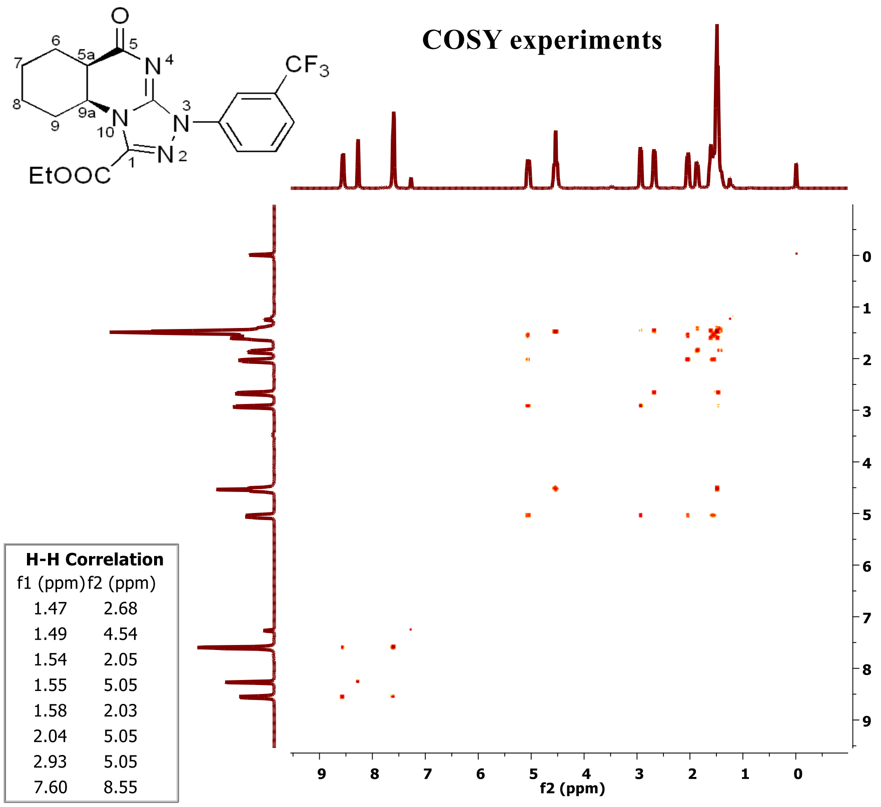

H-H Correlation

f1 (ppm)	f2 (ppm)
1.47	2.68
1.49	4.54
1.54	2.05
1.55	5.05
1.58	2.03
2.04	5.05
2.93	5.05
7.60	8.55

	Parameter	Value (f2, f1)
1	Data File Name	E:/ after/ Hungary work/ 19 20HAS/ 19HASC/ C7/ 19HASC7A-20191214T071428Z-001/ 19HASC7A/ 12/ pdata/ 1/ 2rr
2	Title	19HASC7A
3	Comment	
4	Origin	Bruker BioSpin GmbH
5	Owner	nmrsu
6	Site	
7	Spectrometer	CAB AV4 500 MHZ BASIC
8	Author	
9	Solvent	CDCl3
10	Temperature	298.0
11	Pulse Sequence	cosygpppqf
12	Number of Scans	1
13	Receiver Gain	101
14	Relaxation Delay	1.9631
15	Pulse Width	10.8400
16	Acquisition Time	0.1946
17	Acquisition Date	2019-12-13T13:58:31
18	Modification Date	2019-12-13T06:33:22
19	Spectrometer Frequency	(500.20, 500.20)
20	Spectral Width	(5263.2, 5263.2)
21	Lowest Frequency	(-490.4, -490.4)
22	Nucleus	(1H, 1H)
23	Acquired Size	(1024, 128)
24	Spectral Size	(1024, 1024)

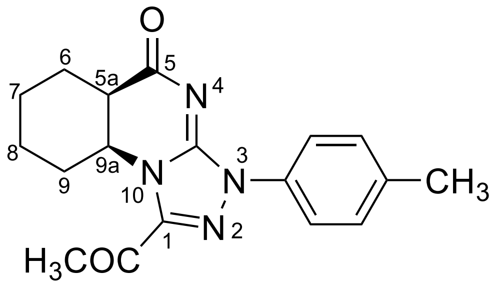

(5a*R,9a*S**)-1-Acetyl-3-(*p*-tolyl)-5a,6,7,8,9,9a-hexahydro-[1,2,4]triazolo[4,3-*a*]quinazoline-5(3*H*)-one (4g)**

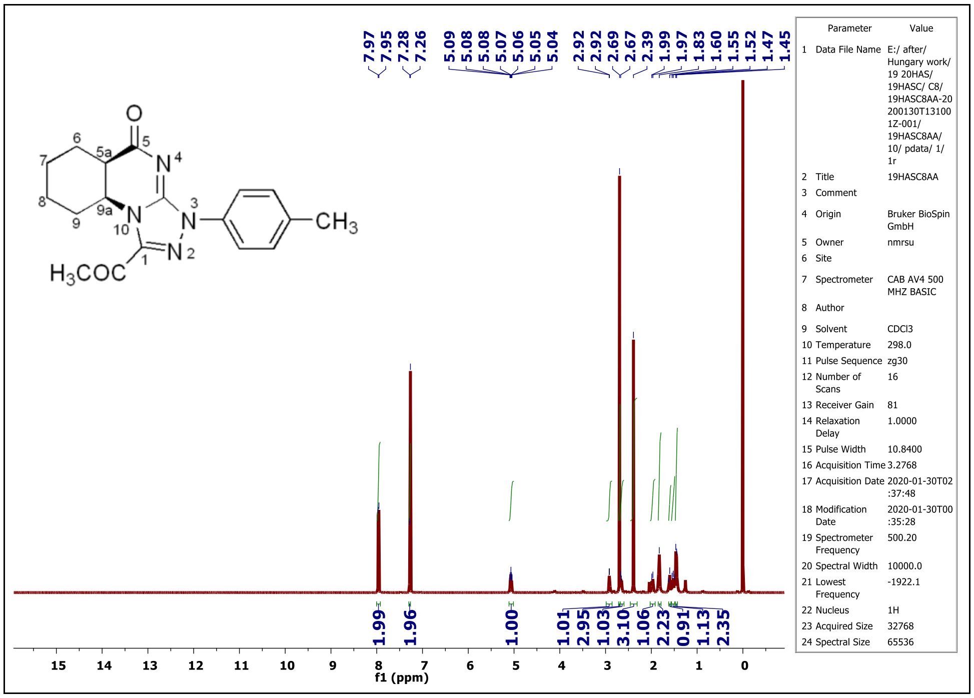

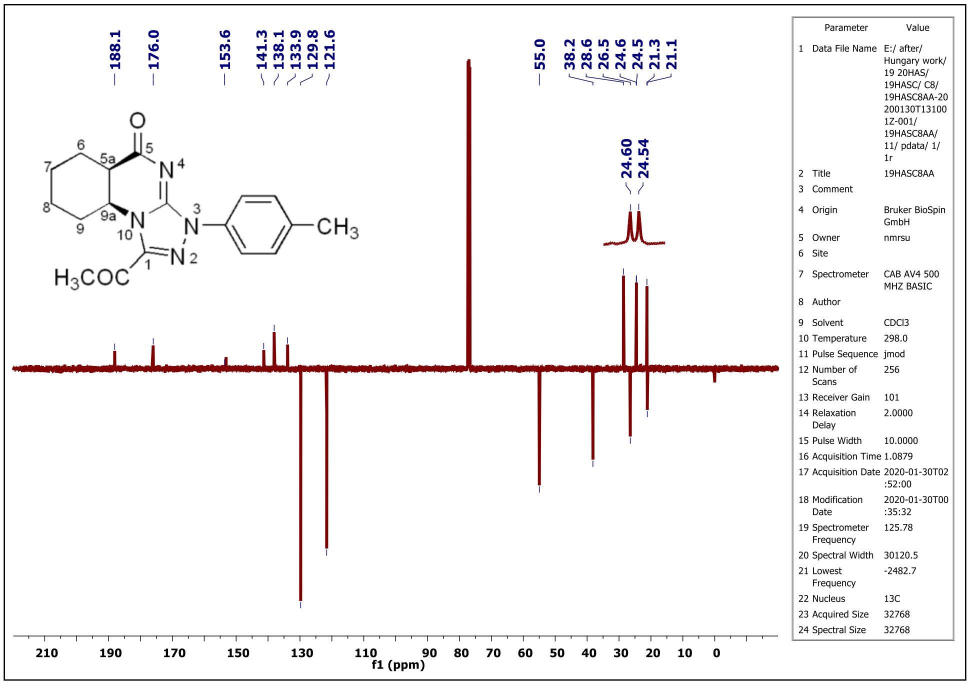

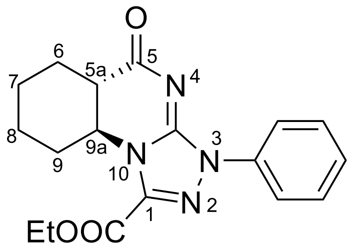

(5a*R,9a*R**)-Ethyl 5-oxo 3-phenyl-3,5,5a,6,7,8,9,9a-octahydro-[1,2,4]triazolo[4,3-*a*]quinazoline-1-carboxylate (5a)**

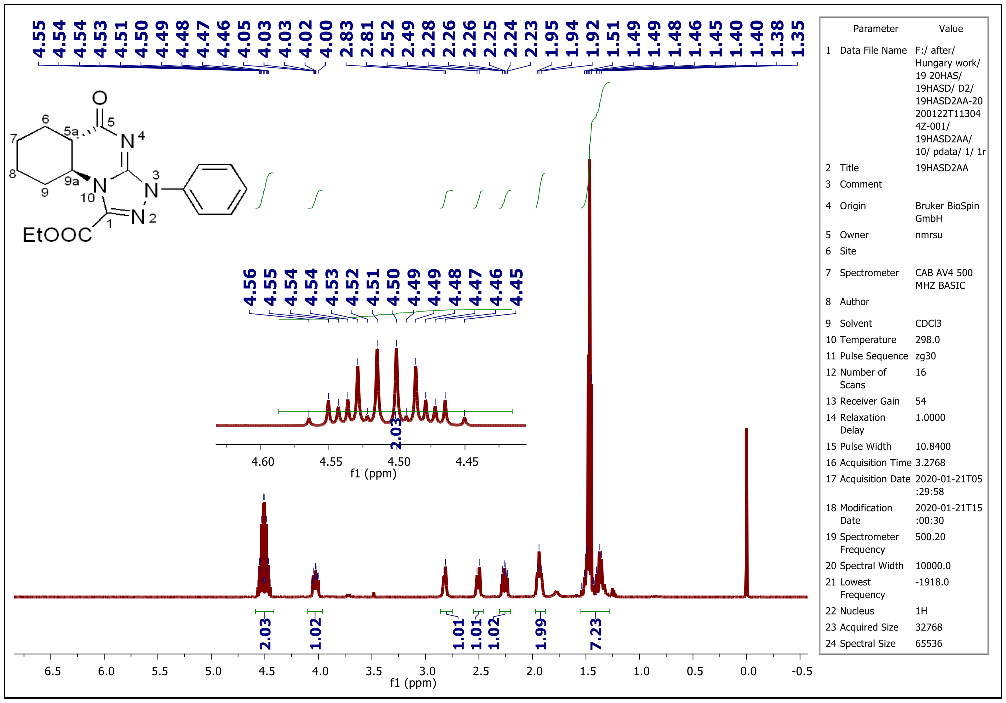

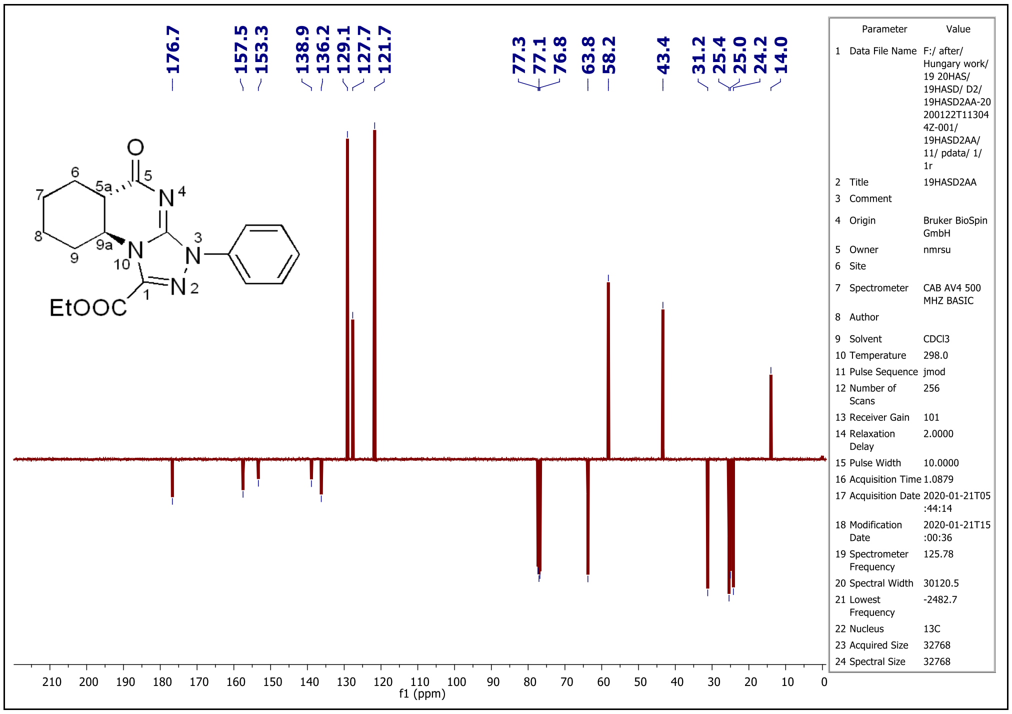

COSY experiments

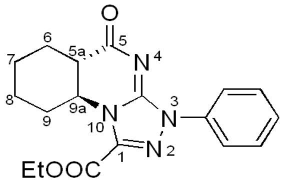

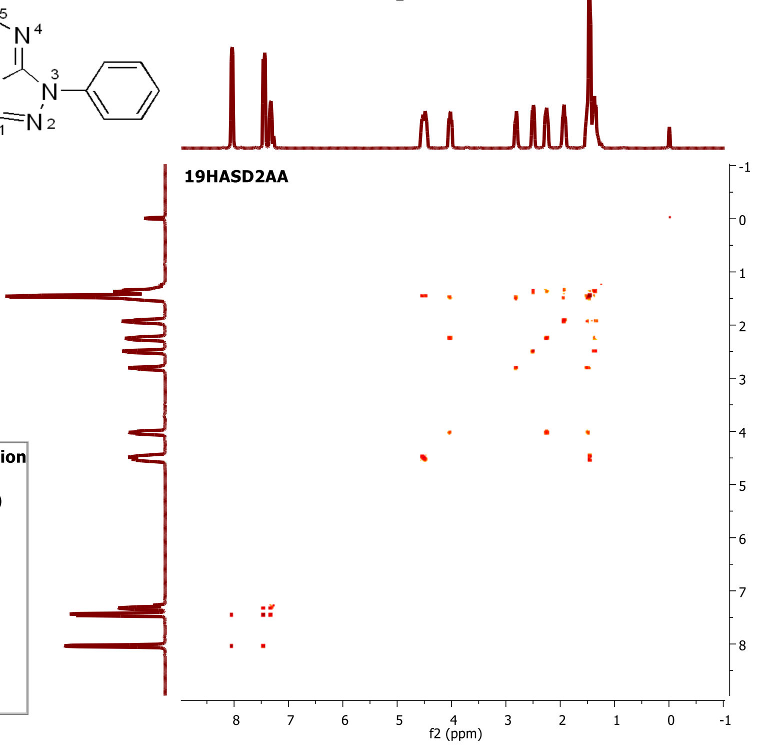

H-H Correlation

f1 (ppm)	f2 (ppm)
1.39	2.51
1.44	4.46
1.45	4.55
1.47	4.02
1.50	2.81
2.26	4.02
7.32	7.45
7.43	8.04

	Parameter	Value (f2, f1)
1	Data File Name	F:/ after/ Hungary work/ 19 20HAS/ 19HASD/ D2/ 19HASD2AA-20200122T113044Z-001/ 19HASD2AA/ 12/ pdata/ 1/ 2rr
2	Title	19HASD2AA
3	Comment	
4	Origin	Bruker BioSpin GmbH
5	Owner	nmrsu
6	Site	
7	Spectrometer	CAB AV4 500 MHZ BASIC
8	Author	
9	Solvent	CDCl3
10	Temperature	298.0
11	Pulse Sequence	cosygpppqf
12	Number of Scans	1
13	Receiver Gain	101
14	Relaxation Delay	1.9529
15	Pulse Width	10.8400
16	Acquisition Time	0.2048
17	Acquisition Date	2020-01-21T05:50:04
18	Modification Date	2020-01-21T15:00:42
19	Spectrometer Frequency	(500.20, 500.20)
20	Spectral Width	(5000.0, 5000.0)
21	Lowest Frequency	(-512.9, -512.9)
22	Nucleus	(1H, 1H)
23	Acquired Size	(1024, 128)
24	Spectral Size	(1024, 1024)

TOCSY experiments

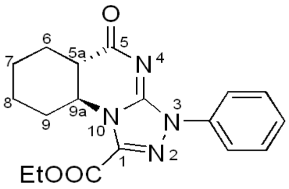

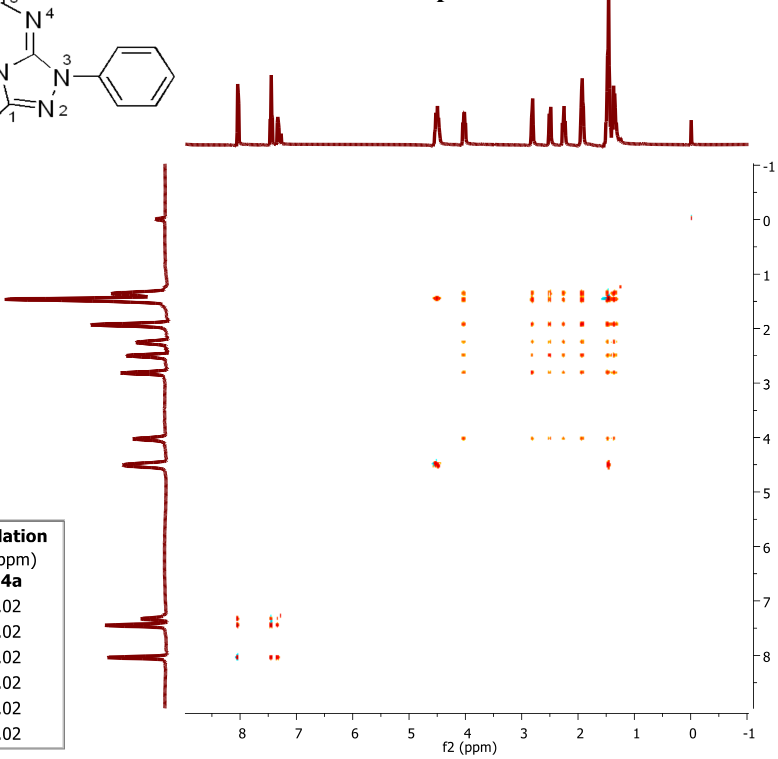

H-H Correlation

f1 (ppm)	f2 (ppm) **H4a**
2.82	4.02
2.50	4.02
2.25	4.02
1.93	4.02
1.48	4.02
1.36	4.02

	Parameter	Value (f2, f1)
1	Data File Name	F:/ after/ Hungary work/ 19 20HAS/ 19HASD/ D2/ 19HASD2AA-20200122T113044Z-001/ 19HASD2AA/ 14/ pdata/ 1/ 2rr
2	Title	19HASD2AA
3	Comment	
4	Origin	Bruker BioSpin GmbH
5	Owner	nmrsu
6	Site	
7	Spectrometer	CAB AV4 500 MHZ BASIC
8	Author	
9	Solvent	CDCl3
10	Temperature	298.0
11	Pulse Sequence	mlevphpp
12	Number of Scans	8
13	Receiver Gain	101
14	Relaxation Delay	2.0020
15	Pulse Width	10.8400
16	Acquisition Time	0.2048
17	Acquisition Date	2020-01-21T07:56:24
18	Modification Date	2020-01-21T15:00:58
19	Spectrometer Frequency	(500.20, 500.20)
20	Spectral Width	(5000.0, 5000.0)
21	Lowest Frequency	(-512.9, -512.9)
22	Nucleus	(1H, 1H)
23	Acquired Size	(1024, 256)
24	Spectral Size	(1024, 1024)

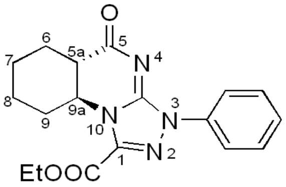

HSQC experiments

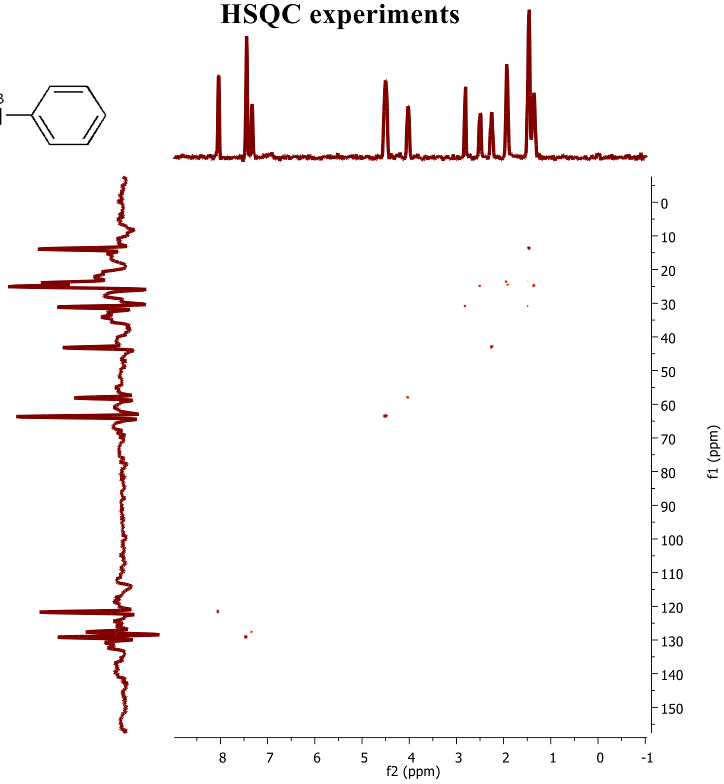

C-H correlation

f1 (ppm)	f2 (ppm)
129.11	7.45
127.66	7.33
121.69	8.04
63.63	4.50
58.15	4.02
43.15	2.25
31.21	1.48
31.05	2.81
25.08	2.49
25.08	1.36
24.76	1.91
23.95	1.95
13.95	1.46

	Parameter	Value (f2, f1)
1	Data File Name	F:/ after/ Hungary work/ 19 20HAS/ 19HASD/ D2/ 19HASD2AA-202 00122T113044Z-001/ 19HASD2AA/ 15/ pdata/ 1/ 2rr
2	Title	19HASD2AA
3	Comment	
4	Origin	Bruker BioSpin GmbH
5	Owner	nmrsu
6	Site	
7	Spectrometer	CAB AV4 500 MHZ BASIC
8	Author	
9	Solvent	CDCl3
10	Temperature	298.0
11	Pulse Sequence	hsqcetgp
12	Number of Scans	2
13	Receiver Gain	101
14	Relaxation Delay	1.5000
15	Pulse Width	10.8400
16	Acquisition Time	0.1024
17	Acquisition Date	2020-01-21T08:11:39
18	Modification Date	2020-01-21T15:01:06
19	Spectrometer Frequency	(500.20, 125.78)
20	Spectral Width	(5000.0, 20752.3)
21	Lowest Frequency	(-512.9, -943.0)
22	Nucleus	(1H, 13C)
23	Acquired Size	(512, 256)
24	Spectral Size	(1024, 1024)

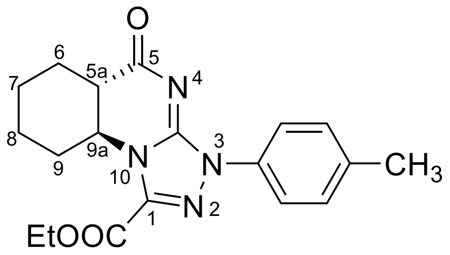

(5a*R,9a*R**)-Ethyl 5-oxo 3-(*p*-tolyl)-3,5,5a,6,7,8,9,9a-octahydro-[1,2,4]triazolo[4,3-*a*]quinazoline-1-carboxylate (5b)**

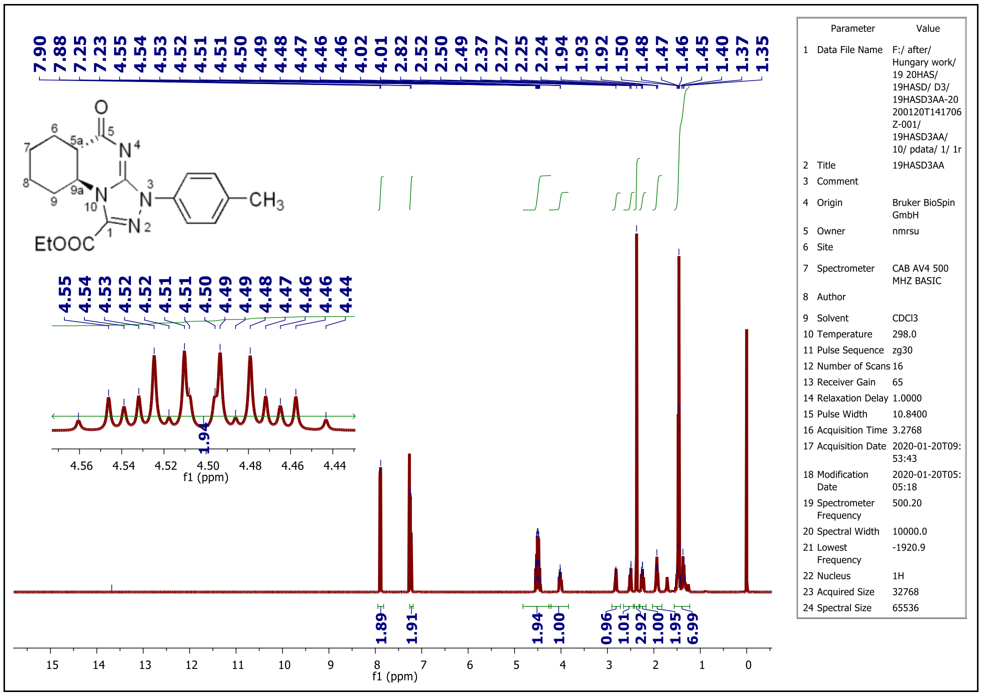

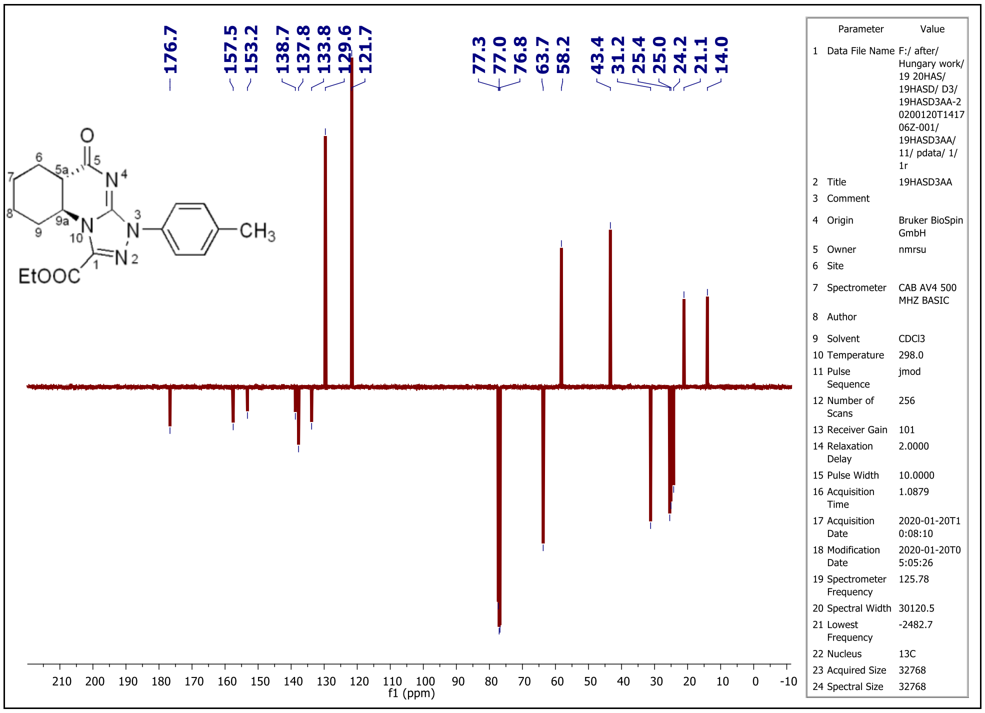

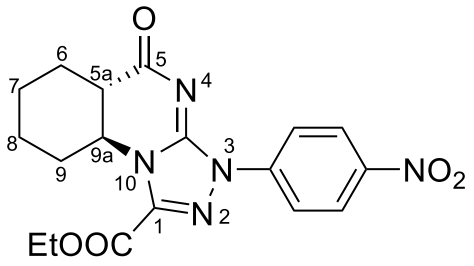

(5a*R,9a*R**)-Ethyl 5-oxo -3-(4-nitrophenyl)-3,5,5a,6,7,8,9,9a-octahydro-[1,2,4]triazolo[4,3-a]quinazoline-1-carboxylate (5c)**

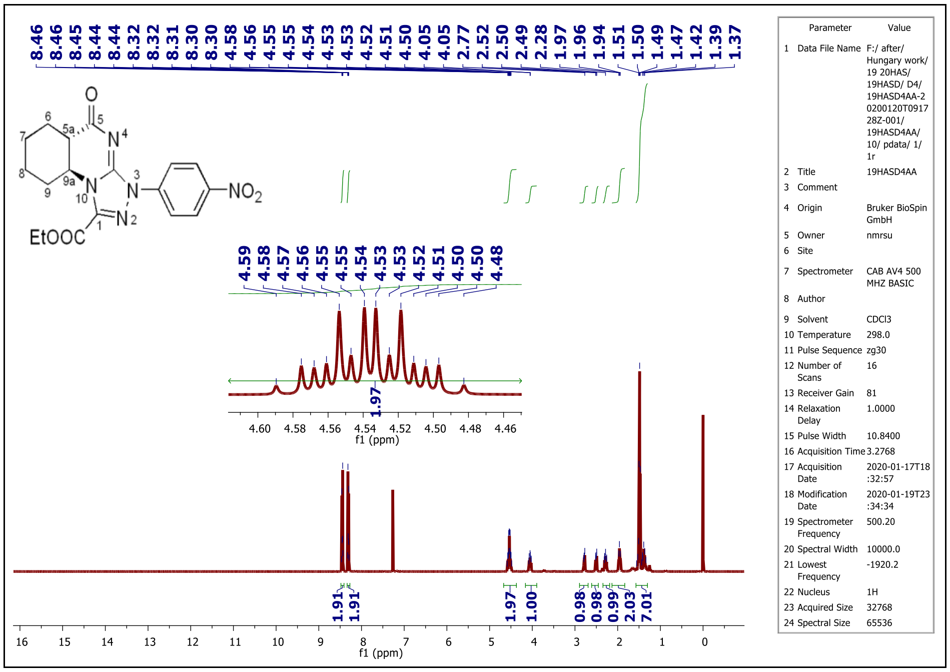

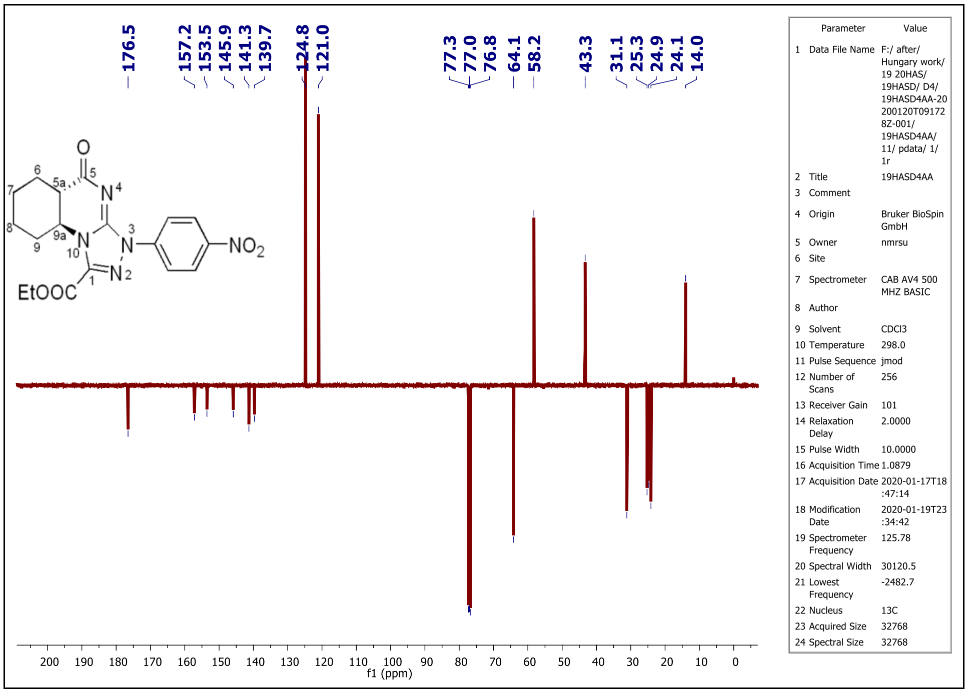

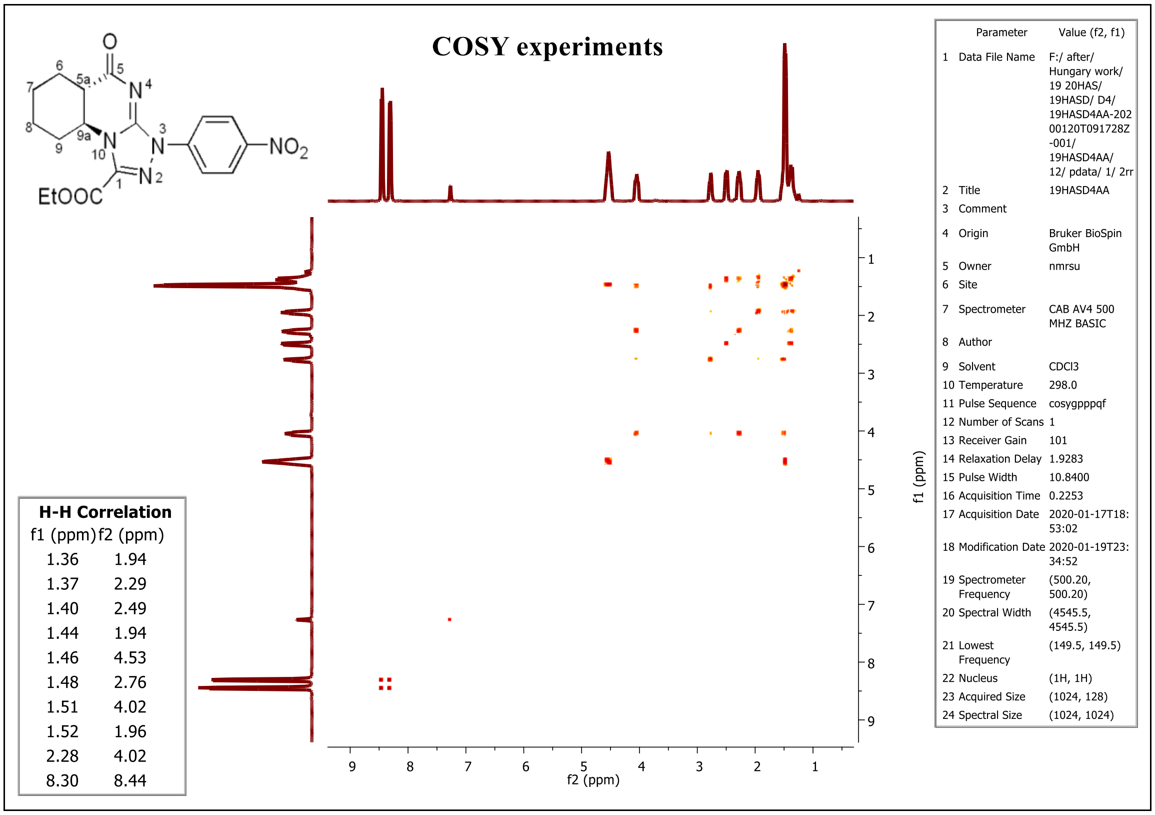

H-H Correlation

f1 (ppm)	f2 (ppm)
1.36	1.94
1.37	2.29
1.40	2.49
1.44	1.94
1.46	4.53
1.48	2.76
1.51	4.02
1.52	1.96
2.28	4.02
8.30	8.44

	Parameter	Value (f2, f1)
1	Data File Name	F:/ after/ Hungary work/ 19 20HAS/ 19HASD/ D4/ 19HASD4AA-20200120T091728Z-001/ 19HASD4AA/ 12/ pdata/ 1/ 2rr
2	Title	19HASD4AA
3	Comment	
4	Origin	Bruker BioSpin GmbH
5	Owner	nmrsu
6	Site	
7	Spectrometer	CAB AV4 500 MHZ BASIC
8	Author	
9	Solvent	CDCl3
10	Temperature	298.0
11	Pulse Sequence	cosygpppqf
12	Number of Scans	1
13	Receiver Gain	101
14	Relaxation Delay	1.9283
15	Pulse Width	10.8400
16	Acquisition Time	0.2253
17	Acquisition Date	2020-01-17T18:53:02
18	Modification Date	2020-01-19T23:34:52
19	Spectrometer Frequency	(500.20, 500.20)
20	Spectral Width	(4545.5, 4545.5)
21	Lowest Frequency	(149.5, 149.5)
22	Nucleus	(1H, 1H)
23	Acquired Size	(1024, 128)
24	Spectral Size	(1024, 1024)

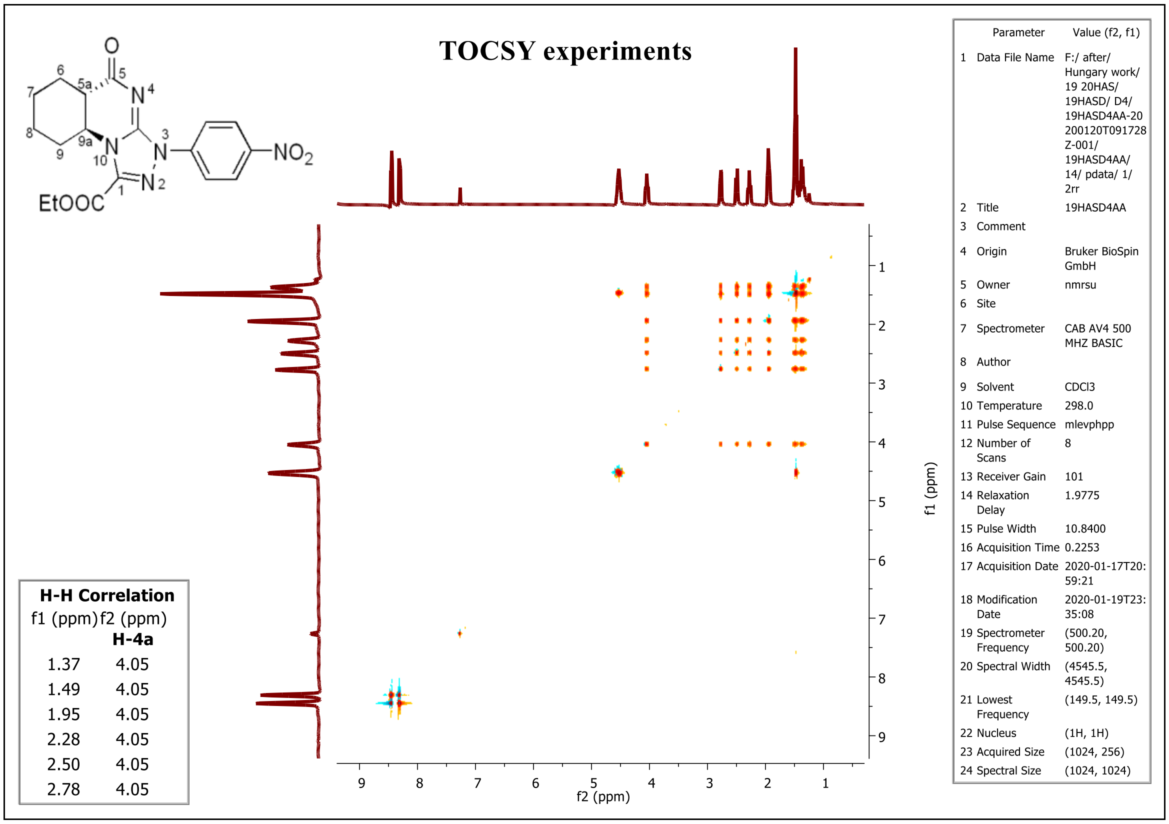

H-H Correlation

f1 (ppm)	f2 (ppm)
	H-4a
1.37	4.05
1.49	4.05
1.95	4.05
2.28	4.05
2.50	4.05
2.78	4.05

	Parameter	Value (f2, f1)
1	Data File Name	F:/ after/ Hungary work/ 19 20HAS/ 19HASD/ D4/ 19HASD4AA-20200120T091728Z-001/ 19HASD4AA/ 14/ pdata/ 1/ 2rr
2	Title	19HASD4AA
3	Comment	
4	Origin	Bruker BioSpin GmbH
5	Owner	nmrsu
6	Site	
7	Spectrometer	CAB AV4 500 MHZ BASIC
8	Author	
9	Solvent	CDCl3
10	Temperature	298.0
11	Pulse Sequence	mlevphpp
12	Number of Scans	8
13	Receiver Gain	101
14	Relaxation Delay	1.9775
15	Pulse Width	10.8400
16	Acquisition Time	0.2253
17	Acquisition Date	2020-01-17T20:59:21
18	Modification Date	2020-01-19T23:35:08
19	Spectrometer Frequency	(500.20, 500.20)
20	Spectral Width	(4545.5, 4545.5)
21	Lowest Frequency	(149.5, 149.5)
22	Nucleus	(1H, 1H)
23	Acquired Size	(1024, 256)
24	Spectral Size	(1024, 1024)

HSQC experiments

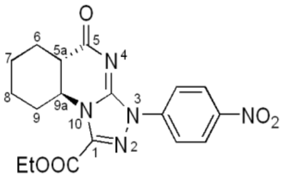

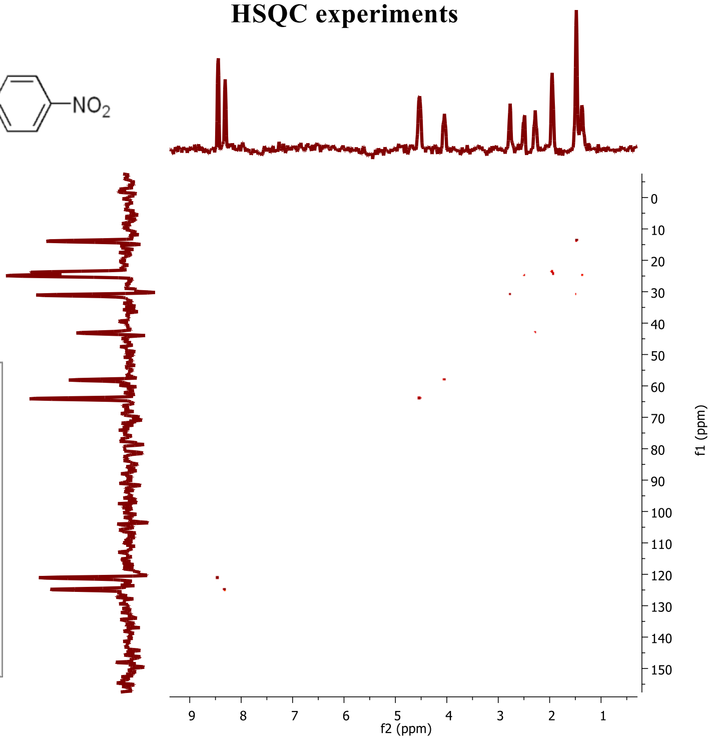

C-H Correlation

f1 (ppm)	f2 (ppm)
13.95	1.48
23.95	1.97
24.92	1.37
25.08	2.48
31.05	2.77
31.05	1.50
43.15	2.28
58.15	4.05
64.11	4.54
121.05	8.44
124.76	8.31

	Parameter	Value (f2, f1)
1	Data File Name	F:/ after/ Hungary work/ 19 20HAS/ 19HASD/ D4/ 19HASD4AA-20200120T091728Z-001/ 19HASD4AA/ 15/ pdata/ 1/ 2rr
2	Title	19HASD4AA
3	Comment	
4	Origin	Bruker BioSpin GmbH
5	Owner	nmrsu
6	Site	
7	Spectrometer	CAB AV4 500 MHZ BASIC
8	Author	
9	Solvent	CDCl3
10	Temperature	298.0
11	Pulse Sequence	hsqcetgp
12	Number of Scans	2
13	Receiver Gain	101
14	Relaxation Delay	1.5000
15	Pulse Width	10.8400
16	Acquisition Time	0.1126
17	Acquisition Date	2020-01-17T21:14:22
18	Modification Date	2020-01-19T23:35:16
19	Spectrometer Frequency	(500.20, 125.78)
20	Spectral Width	(4545.5, 20752.3)
21	Lowest Frequency	(149.5, -943.0)
22	Nucleus	(1H, 13C)
23	Acquired Size	(512, 256)
24	Spectral Size	(1024, 1024)

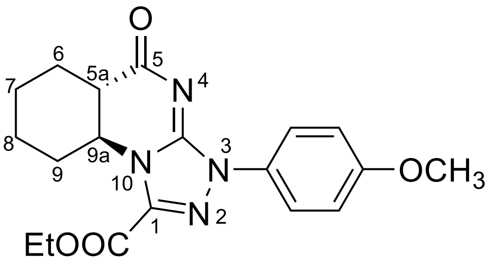

(5a*R,9a*R**)-Ethyl 5-oxo-3-(4-methoxyphenyl)-3,5,5a,6,7,8,9,9a-octahydro-[1,2,4]triazolo[4,3-*a*]quinazoline-1-carboxylate (5d)**

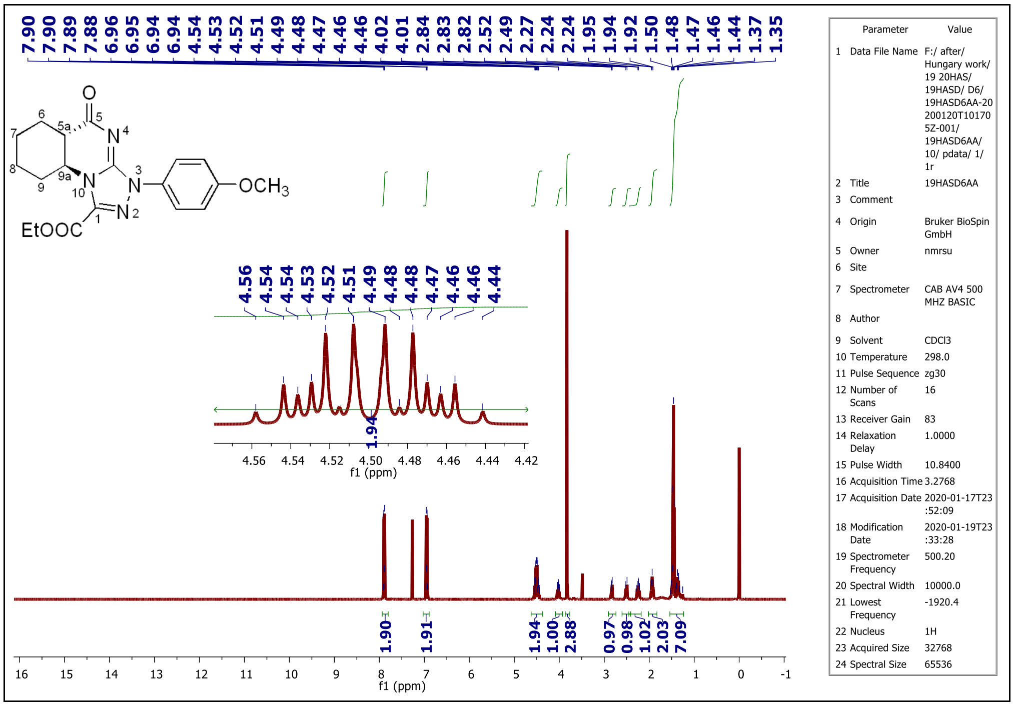

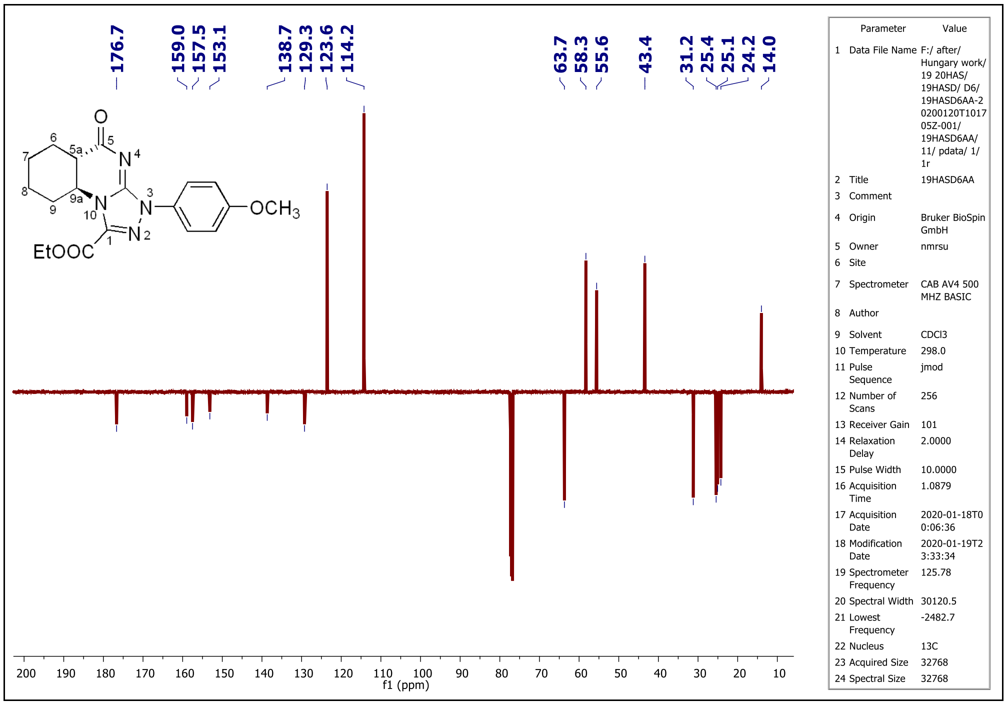

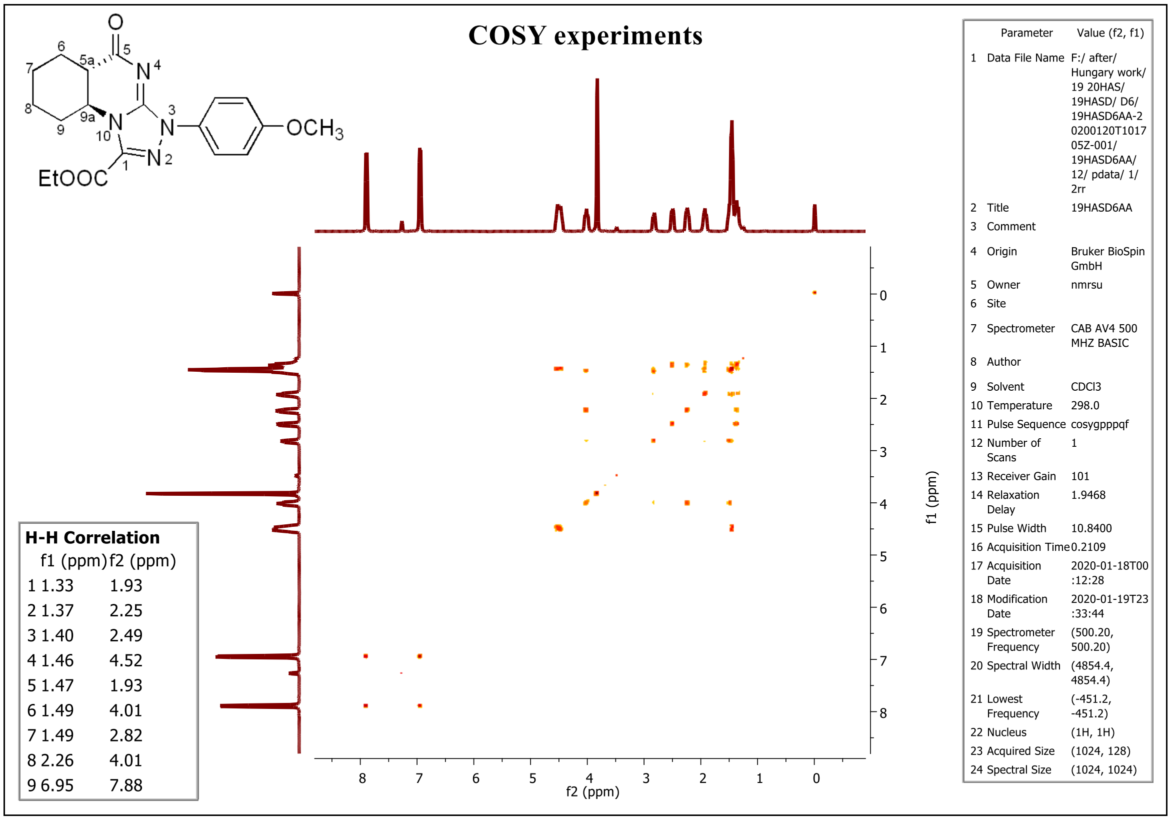
COSY experiments
EtOOC
OCH3
Parameter | Value (f2, f1)
1 Data File Name F:/ after/ Hungary work/ 19 20HAS/ 19HASD/ D6/ 19HASD6AA-20200120T101705Z-001/ 19HASD6AA/ 12/ pdata/ 1/ 2rr
2 Title 19HASD6AA
3 Comment
4 Origin Bruker BioSpin GmbH
5 Owner nmrsu
6 Site
7 Spectrometer CAB AV4 500 MHZ BASIC
8 Author
9 Solvent CDCl3
10 Temperature 298.0
11 Pulse Sequence cosygpppqf
12 Number of Scans 1
13 Receiver Gain 101
14 Relaxation Delay 1.9468
15 Pulse Width 10.8400
16 Acquisition Time 0.2109
17 Acquisition Date 2020-01-18T00:12:28
18 Modification Date 2020-01-19T23:33:44
19 Spectrometer Frequency (500.20, 500.20)
20 Spectral Width (4854.4, 4854.4)
21 Lowest Frequency (-451.2, -451.2)
22 Nucleus (1H, 1H)
23 Acquired Size (1024, 128)
24 Spectral Size (1024, 1024)
H-H Correlation
f1 (ppm) f2 (ppm)
1 1.33 1.93
2 1.37 2.25
3 1.40 2.49
4 1.46 4.52
5 1.47 1.93
6 1.49 4.01
7 1.49 2.82
8 2.26 4.01
9 6.95 7.88
f2 (ppm)
f1 (ppm)

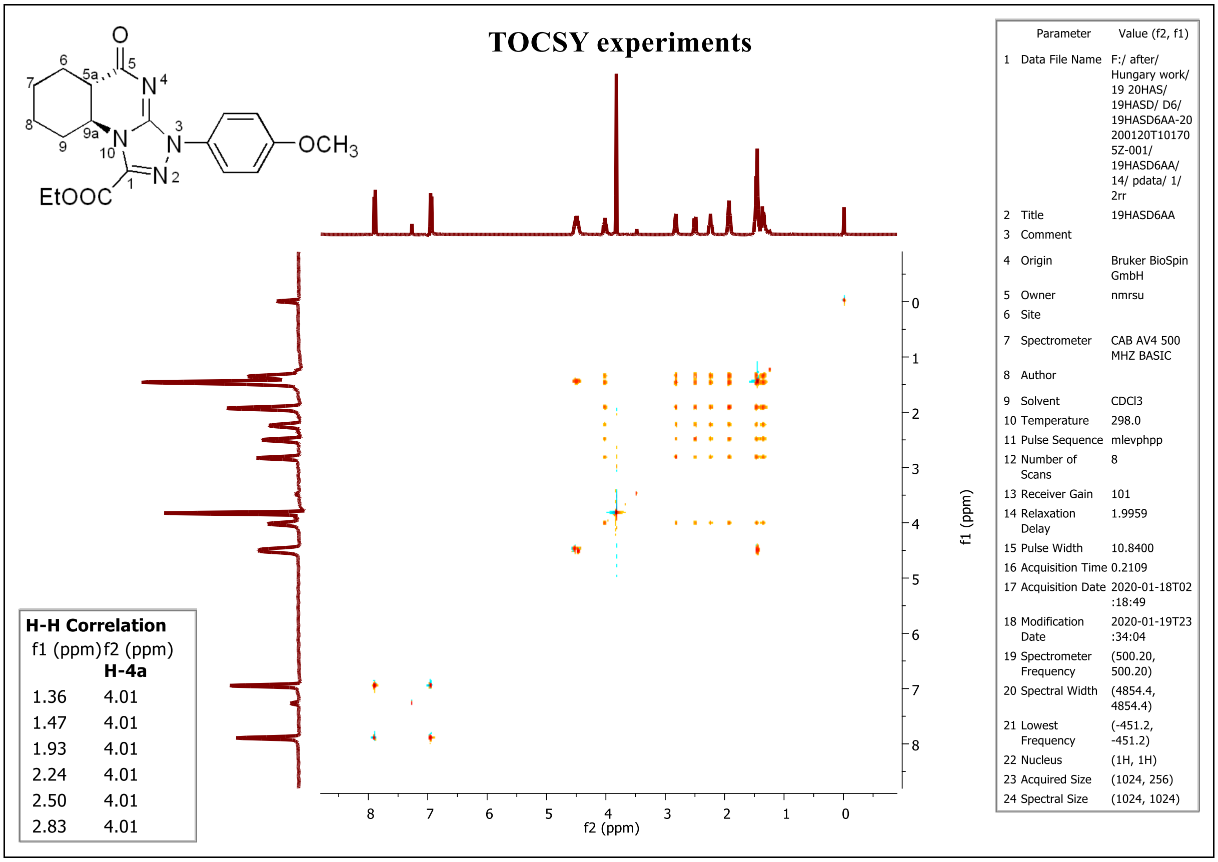
TOCSY experiments
EtOOC
OCH3
Parameter | Value (f2, f1)
1 Data File Name F:/ after/ Hungary work/ 19 20HAS/ 19HASD/ D6/ 19HASD6AA-20200120T101705Z-001/ 19HASD6AA/ 14/ pdata/ 1/ 2rr
2 Title 19HASD6AA
3 Comment
4 Origin Bruker BioSpin GmbH
5 Owner nmrsu
6 Site
7 Spectrometer CAB AV4 500 MHZ BASIC
8 Author
9 Solvent CDCl3
10 Temperature 298.0
11 Pulse Sequence mlevphpp
12 Number of Scans 8
13 Receiver Gain 101
14 Relaxation Delay 1.9959
15 Pulse Width 10.8400
16 Acquisition Time 0.2109
17 Acquisition Date 2020-01-18T02:18:49
18 Modification Date 2020-01-19T23:34:04
19 Spectrometer Frequency (500.20, 500.20)
20 Spectral Width (4854.4, 4854.4)
21 Lowest Frequency (-451.2, -451.2)
22 Nucleus (1H, 1H)
23 Acquired Size (1024, 256)
24 Spectral Size (1024, 1024)
H-H Correlation
f1 (ppm) f2 (ppm)
H-4a
1.36 4.01
1.47 4.01
1.93 4.01
2.24 4.01
2.50 4.01
2.83 4.01
f2 (ppm)
f1 (ppm)

HSQC experiments

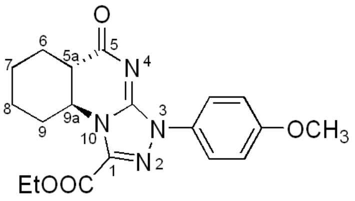

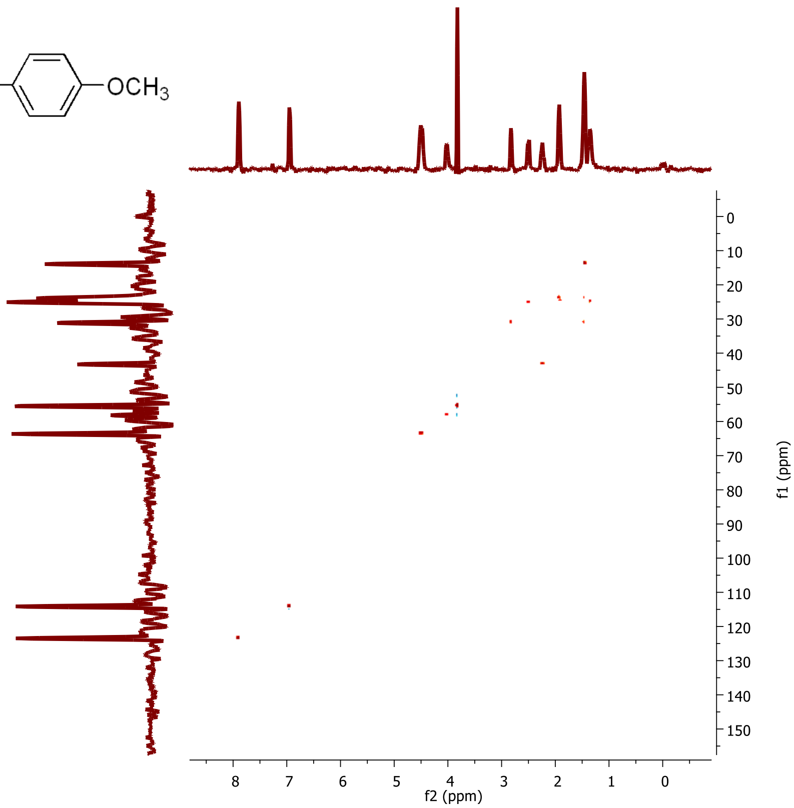

C-H Correlation

f1 (ppm)	f2 (ppm)
13.95	1.45
23.95	1.94
25.08	1.36
25.24	2.49
31.05	2.83
43.31	2.24
55.40	3.82
58.15	4.01
63.63	4.49
114.11	6.94
123.47	7.89

	Parameter	Value (f2, f1)
1	Data File Name	F:/ after/ Hungary work/ 19 20HAS/ 19HASD/ D6/ 19HASD6AA-20200120T101705Z-001/ 19HASD6AA/ 15/ pdata/ 1/ 2rr
2	Title	19HASD6AA
3	Comment	
4	Origin	Bruker BioSpin GmbH
5	Owner	nmrsu
6	Site	
7	Spectrometer	CAB AV4 500 MHZ BASIC
8	Author	
9	Solvent	CDCl3
10	Temperature	298.0
11	Pulse Sequence	hsqcetgp
12	Number of Scans	2
13	Receiver Gain	101
14	Relaxation Delay	1.5000
15	Pulse Width	10.8400
16	Acquisition Time	0.1055
17	Acquisition Date	2020-01-18T02:33:50
18	Modification Date	2020-01-19T23:34:10
19	Spectrometer Frequency	(500.20, 125.78)
20	Spectral Width	(4854.4, 20752.3)
21	Lowest Frequency	(-451.2, -943.0)
22	Nucleus	(1H, 13C)
23	Acquired Size	(512, 256)
24	Spectral Size	(1024, 1024)

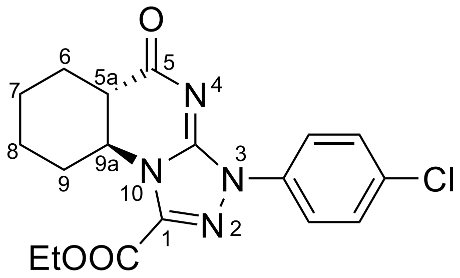

(5a*R,9a*R**)-Ethyl 5-oxo-3-(4-chlorophenyl)-3,5,5a,6,7,8,9,9a-octahydro-[1,2,4]triazolo[4,3-a] quinazoline-1- carboxylate (5e)**

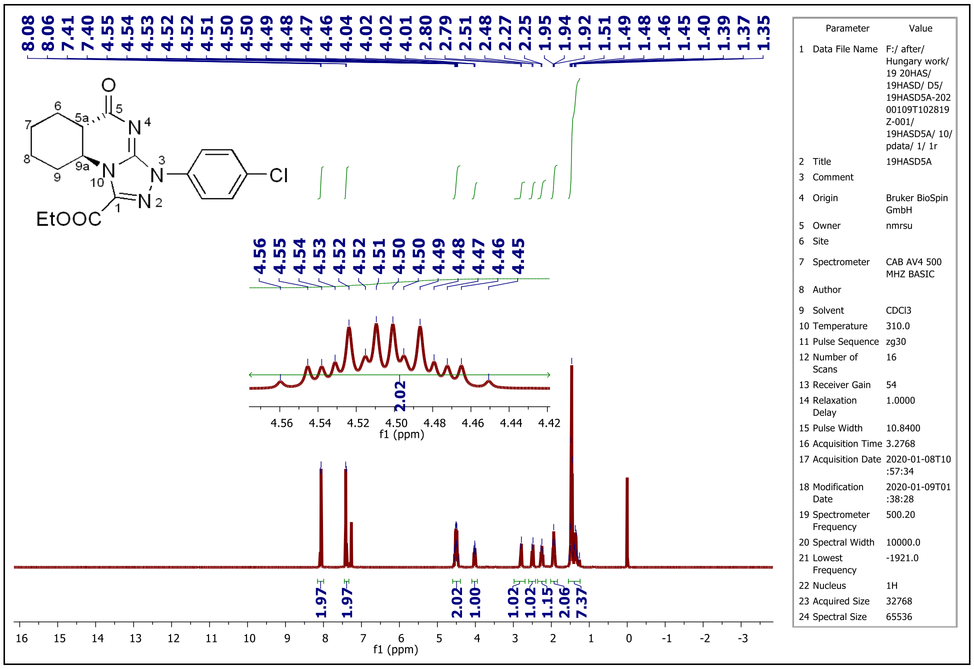

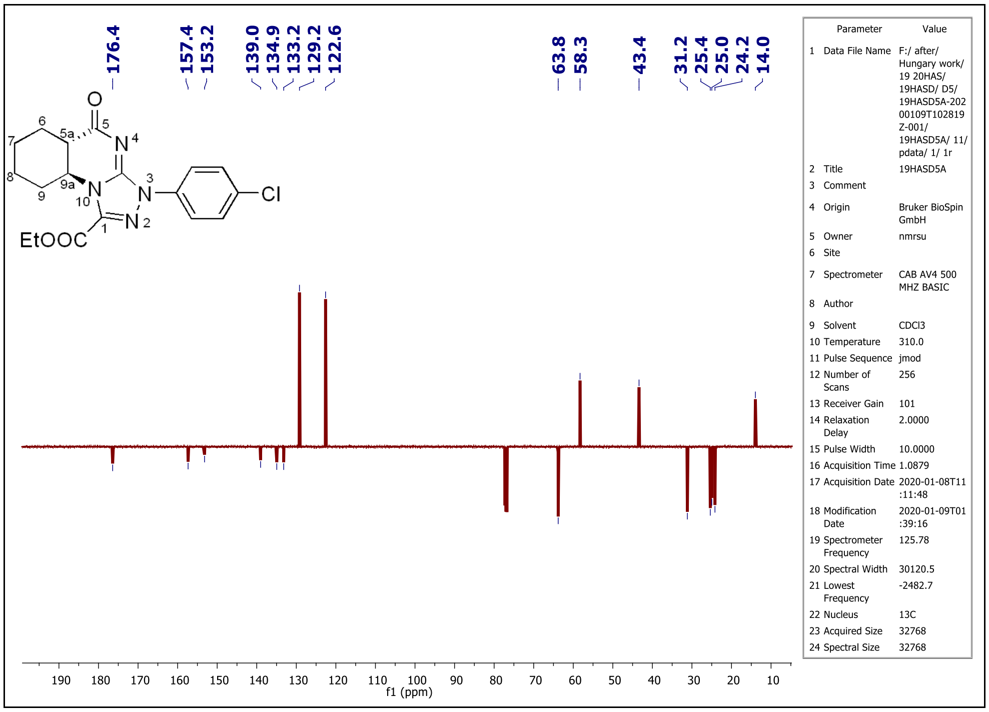

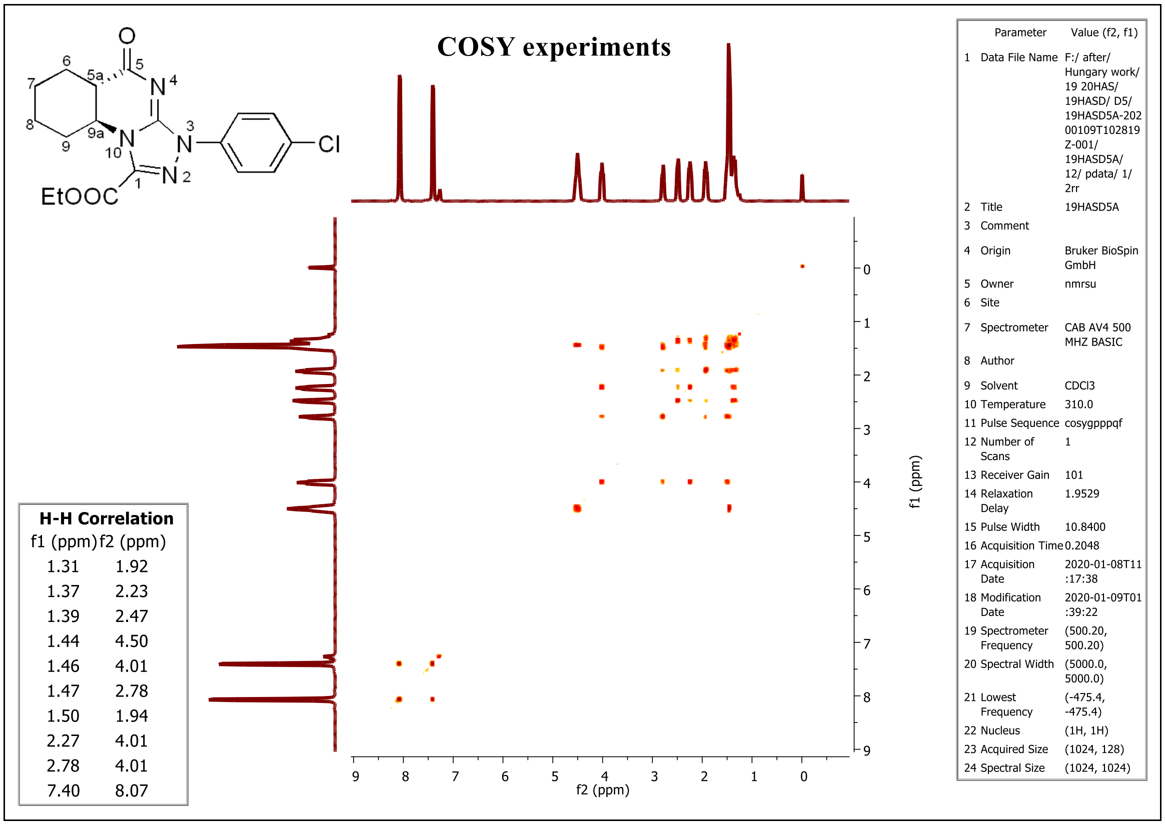

H-H Correlation

f1 (ppm)	f2 (ppm)
1.31	1.92
1.37	2.23
1.39	2.47
1.44	4.50
1.46	4.01
1.47	2.78
1.50	1.94
2.27	4.01
2.78	4.01
7.40	8.07

	Parameter	Value (f2, f1)
1	Data File Name	F:/ after/ Hungary work/ 19 20HAS/ 19HASD/ D5/ 19HASD5A-20200109T102819Z-001/ 19HASD5A/ 12/ pdata/ 1/ 2rr
2	Title	19HASD5A
3	Comment	
4	Origin	Bruker BioSpin GmbH
5	Owner	nmrsu
6	Site	
7	Spectrometer	CAB AV4 500 MHZ BASIC
8	Author	
9	Solvent	CDCl3
10	Temperature	310.0
11	Pulse Sequence	cosygpppqf
12	Number of Scans	1
13	Receiver Gain	101
14	Relaxation Delay	1.9529
15	Pulse Width	10.8400
16	Acquisition Time	0.2048
17	Acquisition Date	2020-01-08T11:17:38
18	Modification Date	2020-01-09T01:39:22
19	Spectrometer Frequency	(500.20, 500.20)
20	Spectral Width	(5000.0, 5000.0)
21	Lowest Frequency	(-475.4, -475.4)
22	Nucleus	(1H, 1H)
23	Acquired Size	(1024, 128)
24	Spectral Size	(1024, 1024)

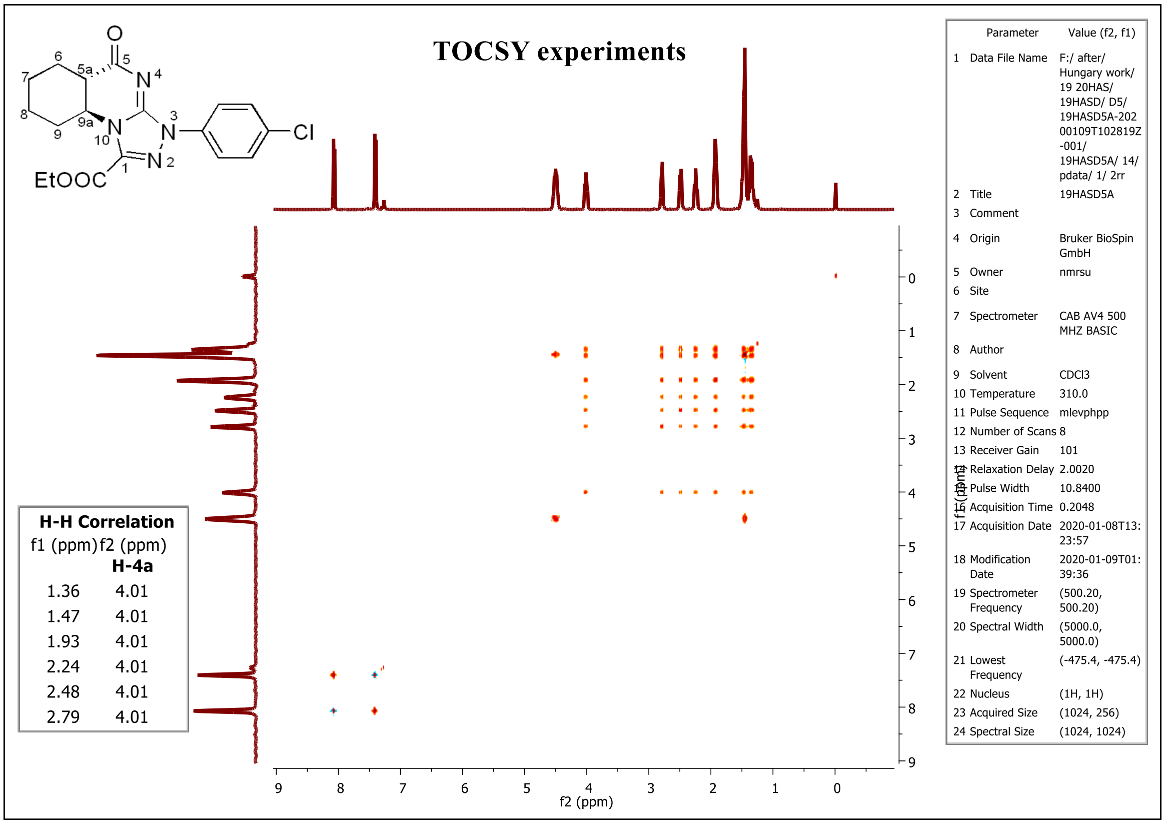

H-H Correlation

f1 (ppm)	f2 (ppm)
	**H-4a**
1.36	4.01
1.47	4.01
1.93	4.01
2.24	4.01
2.48	4.01
2.79	4.01

	Parameter	Value (f2, f1)
1	Data File Name	F:/ after/ Hungary work/ 19 20HAS/ 19HASD/ D5/ 19HASD5A-20200109T102819Z-001/ 19HASD5A/ 14/ pdata/ 1/ 2rr
2	Title	19HASD5A
3	Comment	
4	Origin	Bruker BioSpin GmbH
5	Owner	nmrsu
6	Site	
7	Spectrometer	CAB AV4 500 MHZ BASIC
8	Author	
9	Solvent	CDCl3
10	Temperature	310.0
11	Pulse Sequence	mlevphpp
12	Number of Scans	8
13	Receiver Gain	101
14	Relaxation Delay	2.0020
15	Pulse Width	10.8400
16	Acquisition Time	0.2048
17	Acquisition Date	2020-01-08T13:23:57
18	Modification Date	2020-01-09T01:39:36
19	Spectrometer Frequency	(500.20, 500.20)
20	Spectral Width	(5000.0, 5000.0)
21	Lowest Frequency	(-475.4, -475.4)
22	Nucleus	(1H, 1H)
23	Acquired Size	(1024, 256)
24	Spectral Size	(1024, 1024)

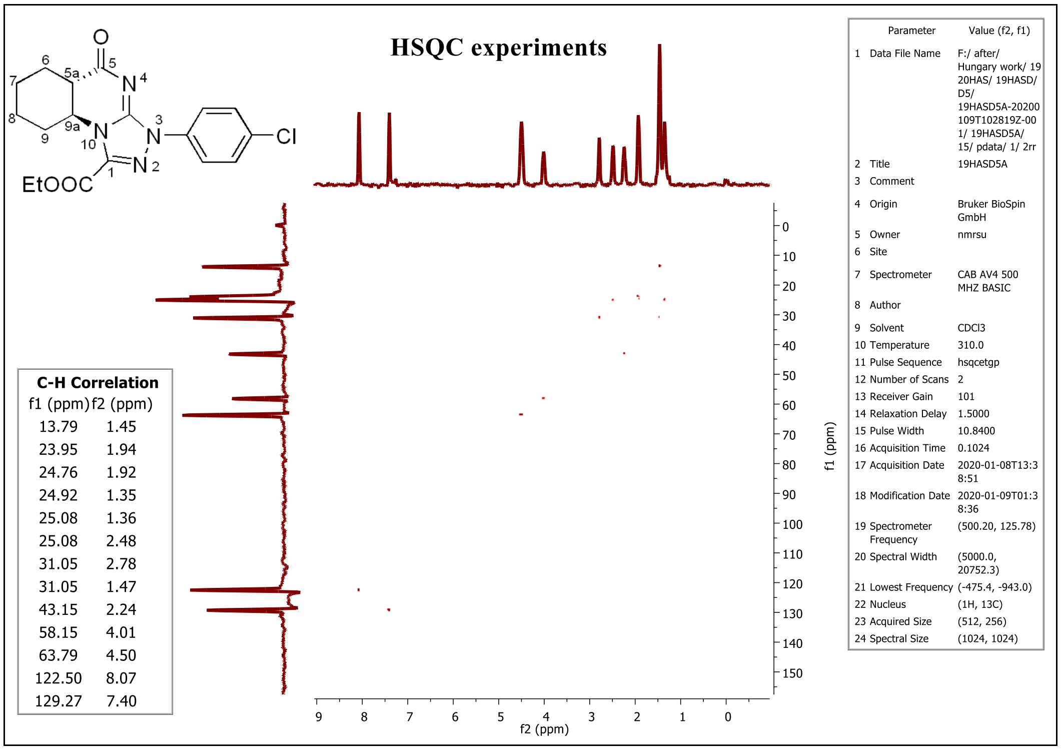

C-H Correlation

f1 (ppm)	f2 (ppm)
13.79	1.45
23.95	1.94
24.76	1.92
24.92	1.35
25.08	1.36
25.08	2.48
31.05	2.78
31.05	1.47
43.15	2.24
58.15	4.01
63.79	4.50
122.50	8.07
129.27	7.40

	Parameter	Value (f2, f1)
1	Data File Name	F:/ after/ Hungary work/ 1920HAS/ 19HASD/ D5/ 19HASD5A-20200109T102819Z-001/ 19HASD5A/ 15/ pdata/ 1/ 2rr
2	Title	19HASD5A
3	Comment	
4	Origin	Bruker BioSpin GmbH
5	Owner	nmrsu
6	Site	
7	Spectrometer	CAB AV4 500 MHZ BASIC
8	Author	
9	Solvent	CDCl3
10	Temperature	310.0
11	Pulse Sequence	hsqcetgp
12	Number of Scans	2
13	Receiver Gain	101
14	Relaxation Delay	1.5000
15	Pulse Width	10.8400
16	Acquisition Time	0.1024
17	Acquisition Date	2020-01-08T13:38:51
18	Modification Date	2020-01-09T01:38:36
19	Spectrometer Frequency	(500.20, 125.78)
20	Spectral Width	(5000.0, 20752.3)
21	Lowest Frequency	(-475.4, -943.0)
22	Nucleus	(1H, 13C)
23	Acquired Size	(512, 256)
24	Spectral Size	(1024, 1024)

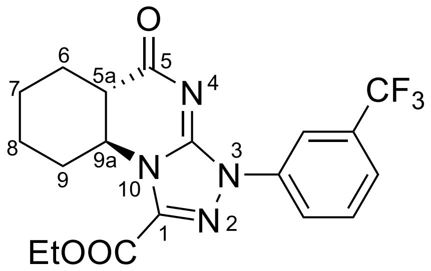

(5a*R,9a*R**)-Ethyl 5-oxo-3-(4-(trifluoromethyl)phenyl)-3,5,5a,6,7,8,9,9a-octahydro-[1,2,4]triazolo[4,3-*a*]quinazoline-1-carboxylate (5f)**

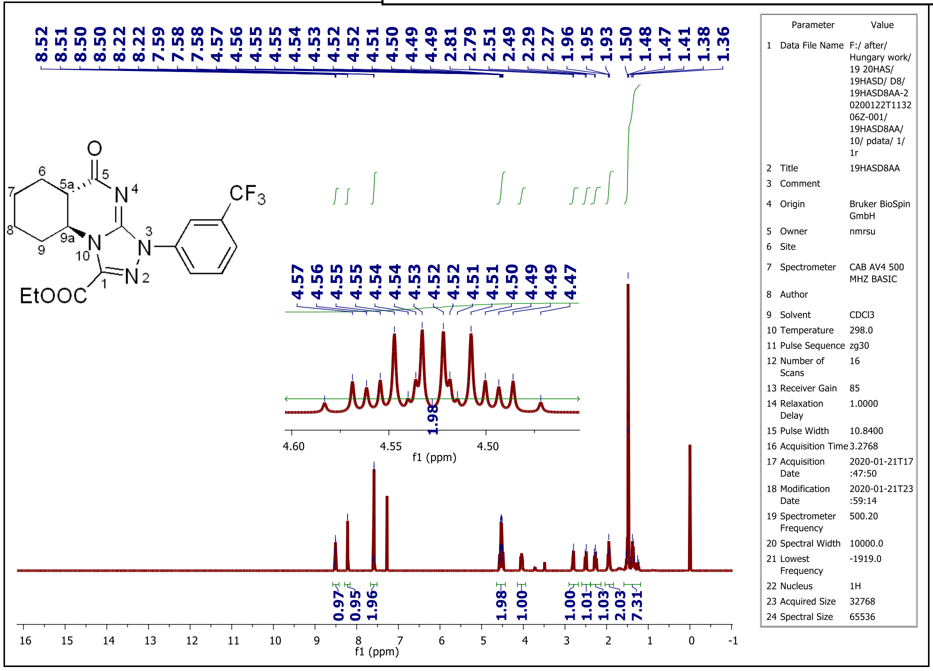

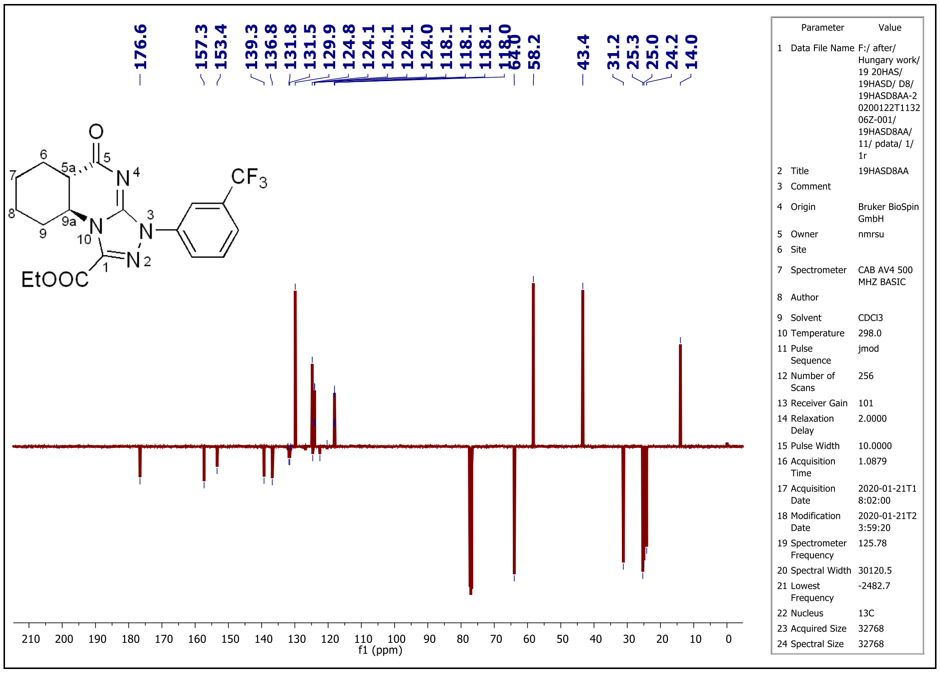

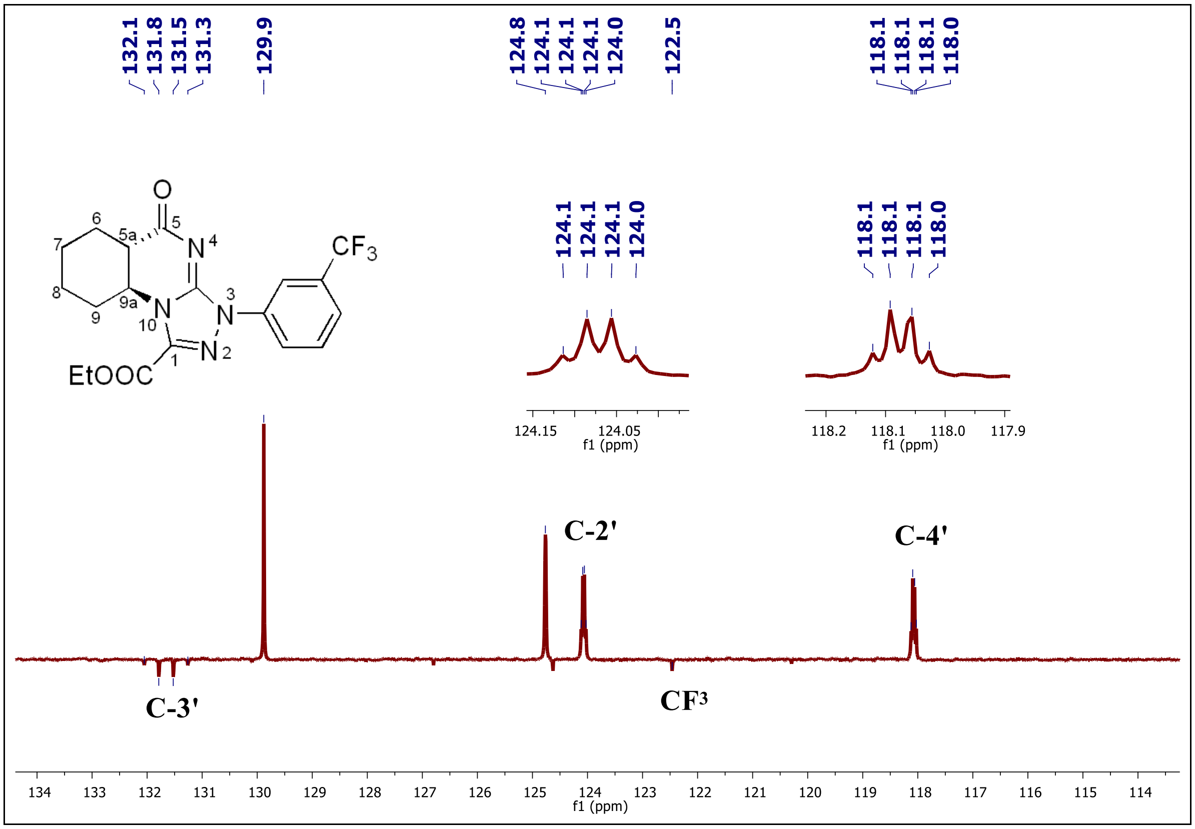

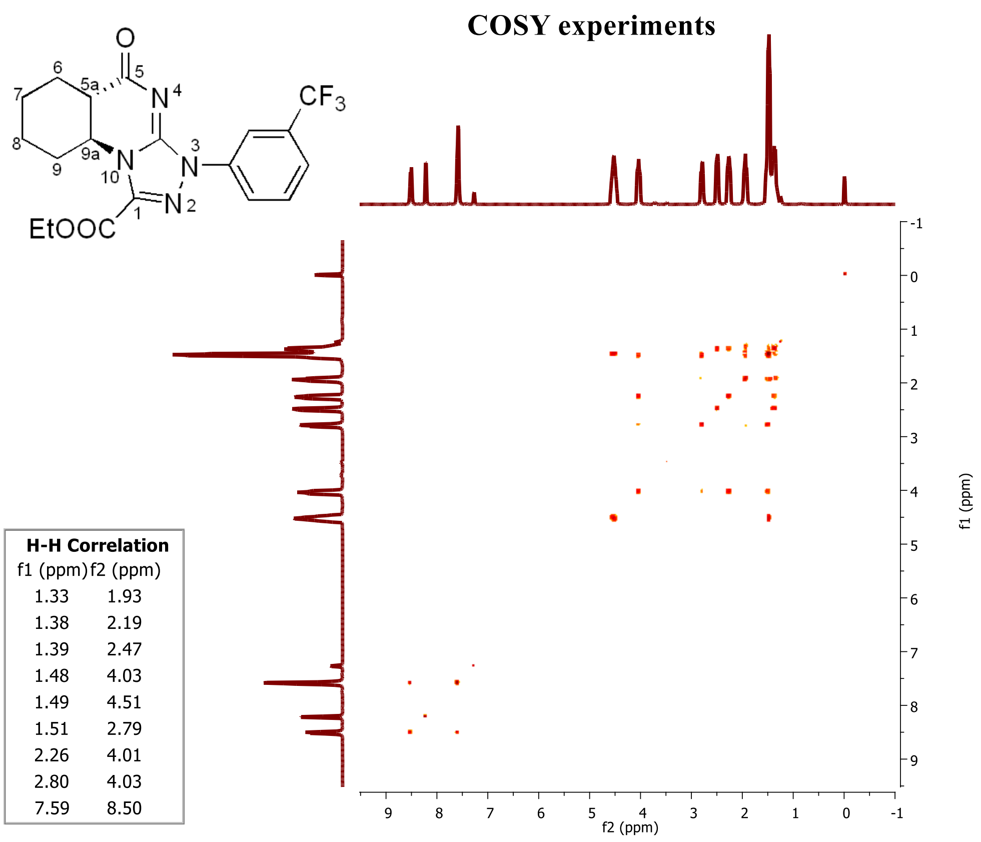

H-H Correlation	
f1 (ppm)	f2 (ppm)
1.33	1.93
1.38	2.19
1.39	2.47
1.48	4.03
1.49	4.51
1.51	2.79
2.26	4.01
2.80	4.03
7.59	8.50

	Parameter	Value (f2, f1)
1	Data File Name	F:/ after/ Hungary work/ 19 20HAS/ 19HASD/ D8/ 19HASD8AA-20 200122T11320 6Z-001/ 19HASD8AA/ 12/ pdata/ 1/ 2rr
2	Title	19HASD8AA
3	Comment	
4	Origin	Bruker BioSpin GmbH
5	Owner	nmrsu
6	Site	
7	Spectrometer	CAB AV4 500 MHZ BASIC
8	Author	
9	Solvent	CDCl3
10	Temperature	298.0
11	Pulse Sequence	cosygpppqf
12	Number of Scans	1
13	Receiver Gain	101
14	Relaxation Delay	1.9611
15	Pulse Width	10.8400
16	Acquisition Time	0.1946
17	Acquisition Date	2020-01-21T18:07:49
18	Modification Date	2020-01-21T23:59:28
19	Spectrometer Frequency	(500.20, 500.20)
20	Spectral Width	(5263.2, 5263.2)
21	Lowest Frequency	(-502.2, -502.2)
22	Nucleus	(1H, 1H)
23	Acquired Size	(1024, 128)
24	Spectral Size	(1024, 1024)

TOCSY experiments

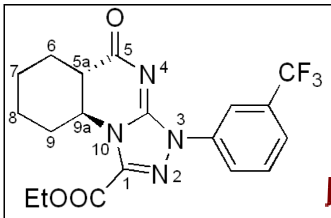

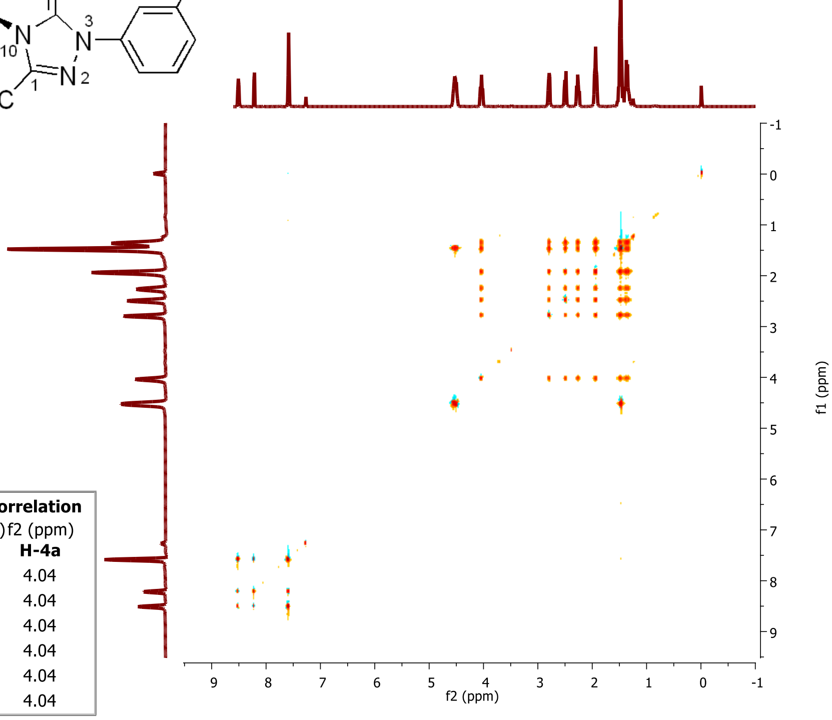

H-H Correlation

f1 (ppm)	f2 (ppm)
	**H-4a**
1.36	4.04
1.48	4.04
1.94	4.04
2.27	4.04
2.49	4.04
2.80	4.04

	Parameter	Value (f2, f1)
1	Data File Name	F:/ after/ Hungary work/ 19 20HAS/ 19HASD/ D8/ 19HASD8AA-20200122T113206Z-001/ 19HASD8AA/ 14/ pdata/ 1/ 2rr
2	Title	19HASD8AA
3	Comment	
4	Origin	Bruker BioSpin GmbH
5	Owner	nmrsu
6	Site	
7	Spectrometer	CAB AV4 500 MHZ BASIC
8	Author	
9	Solvent	CDCl3
10	Temperature	298.0
11	Pulse Sequence	mlevphpp
12	Number of Scans	8
13	Receiver Gain	101
14	Relaxation Delay	2.0102
15	Pulse Width	10.8400
16	Acquisition Time	0.1946
17	Acquisition Date	2020-01-21T20:14:02
18	Modification Date	2020-01-21T23:59:44
19	Spectrometer Frequency	(500.20, 500.20)
20	Spectral Width	(5263.2, 5263.2)
21	Lowest Frequency	(-502.2, -502.2)
22	Nucleus	(1H, 1H)
23	Acquired Size	(1024, 256)
24	Spectral Size	(1024, 1024)

HSQC experiments

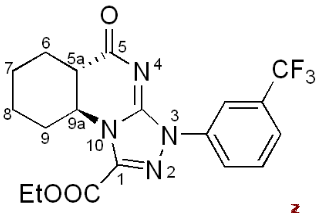

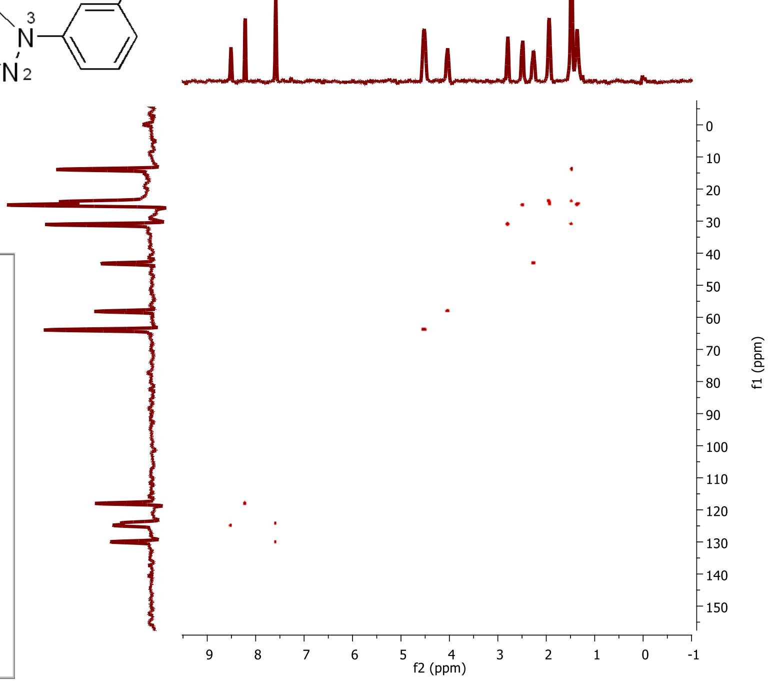

C-H Correlation

f1 (ppm)	f2 (ppm)
13.95	1.47
23.95	1.96
23.95	1.49
24.76	1.93
24.92	1.36
25.08	2.48
31.05	2.79
31.05	1.49
43.15	2.27
58.15	4.04
63.95	4.52
117.98	8.21
124.11	7.58
124.76	8.51
129.92	7.58

	Parameter	Value (f2, f1)
1	Data File Name	F:/ after/ Hungary work/ 19 20HAS/ 19HASD/ D8/ 19HASD8AA-20200122T113206Z-001/ 19HASD8AA/ 15/ pdata/ 1/ 2rr
2	Title	19HASD8AA
3	Comment	
4	Origin	Bruker BioSpin GmbH
5	Owner	nmrsu
6	Site	
7	Spectrometer	CAB AV4 500 MHZ BASIC
8	Author	
9	Solvent	CDCl3
10	Temperature	298.0
11	Pulse Sequence	hsqcetgp
12	Number of Scans	2
13	Receiver Gain	101
14	Relaxation Delay	1.5000
15	Pulse Width	10.8400
16	Acquisition Time	0.0973
17	Acquisition Date	2020-01-21T20:28:53
18	Modification Date	2020-01-21T23:59:50
19	Spectrometer Frequency	(500.20, 125.78)
20	Spectral Width	(5263.2, 20752.3)
21	Lowest Frequency	(-502.2, -943.0)
22	Nucleus	(1H, 13C)
23	Acquired Size	(512, 256)
24	Spectral Size	(1024, 1024)

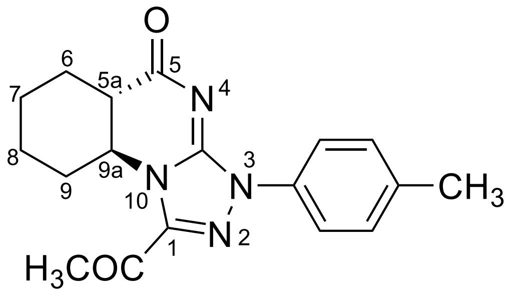

(5a*R,9a*R**)-1-Acetyl-3-(*p*-tolyl)-5a,6,7,8,9,9a-hexahydro[1,2,4]triazolo[4,3-*a*]quinazoline-5(3*H*)-one (5g)**

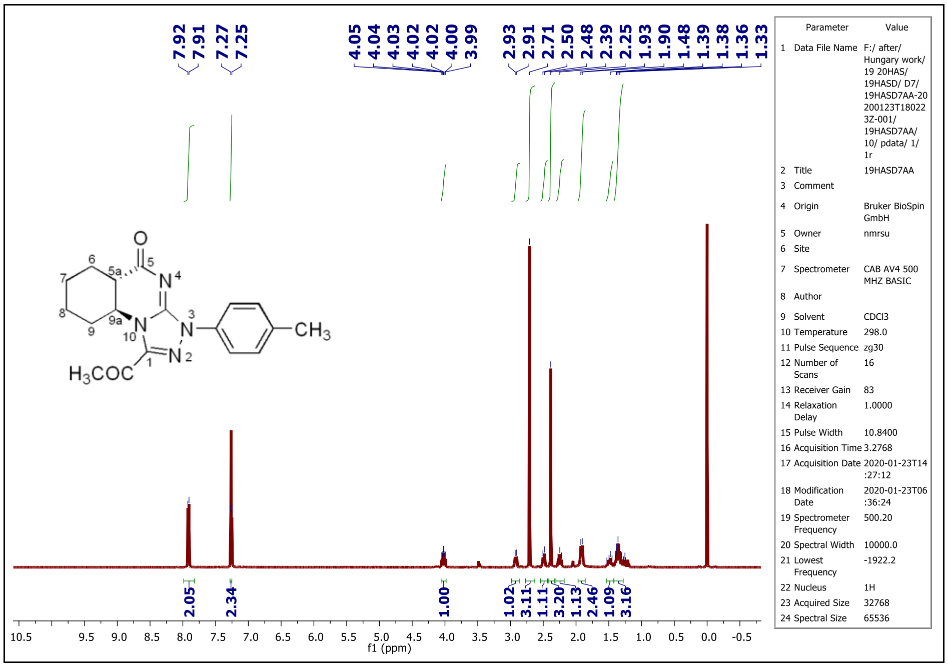

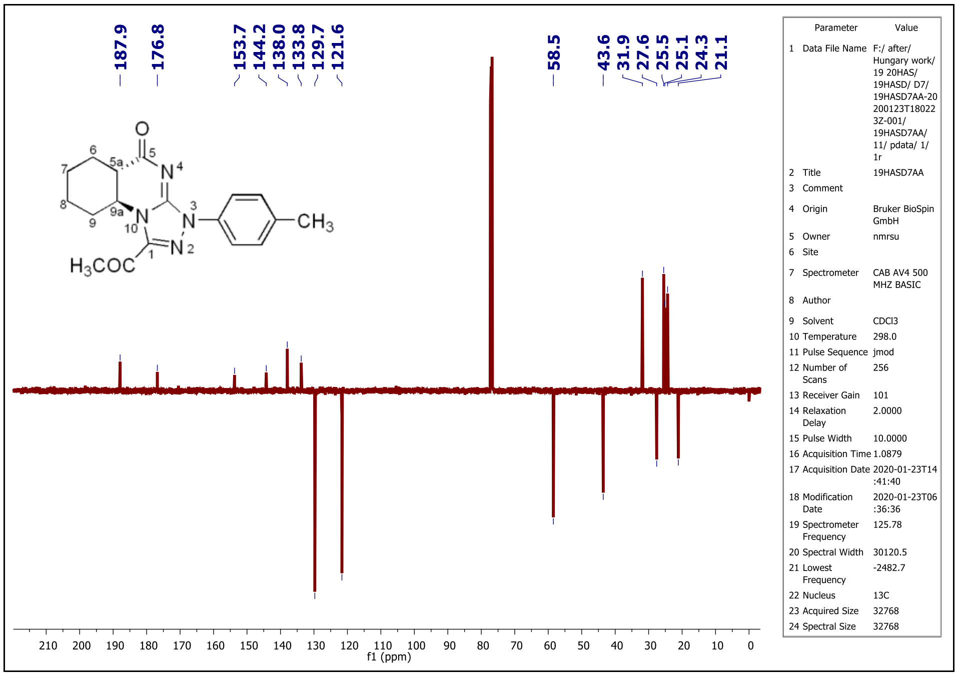

Crystallographic details of compound (5b)

Table S1.

	**5b**
empirical formula	$C_{19}H_{22}N_4O_3$
fw	354.40
temp (K)	120(2)
λ(Å)	1.54184
cryst syst	Monoclinic
space group	$P2_1/c$
a (Å)	10.5009(4)
b (Å)	21.6142(8)
c (Å)	8.0811(4)
β (deg)	112.461(5)
V (Å^3)	1695.02(14)
Z	4
ρ_{calc} (Mg/m^3)	1.389
μ(Mo Kα) (mm^{-1})	0.785
No. reflns.	11923
Unique reflns.	3462
GOOF (F^2)	1.033
R_{int}	0.0390
R1[a] ($I \geq 2\sigma$)	0.0402
wR2[b] ($I \geq 2\sigma$)	0.1000

[a] $R1 = \Sigma||F_o| - |F_c||/\Sigma|F_o|$. [b] $wR2 = [\Sigma[w(F_o^2 - F_c^2)^2]/ \Sigma[w(F_o^2)^2]]^{1/2}$.
